# Why models underestimate West African tropical forest primary productivity

**DOI:** 10.1038/s41467-024-53949-0

**Published:** 2024-11-06

**Authors:** Huanyuan Zhang-Zheng, Xiongjie Deng, Jesús Aguirre-Gutiérrez, Benjamin D. Stocker, Eleanor Thomson, Ruijie Ding, Stephen Adu-Bredu, Akwasi Duah-Gyamfi, Agne Gvozdevaite, Sam Moore, Imma Oliveras Menor, I. Colin Prentice, Yadvinder Malhi

**Affiliations:** 1 Environmental Change Institute, School of Geography and the Environment, University of Oxford, Oxford, United Kingdom; 2 Leverhulme Centre for Nature Recovery, University of Oxford, Oxford, UK; 3 Institute of Geography, University of Bern, Hallerstrasse 12, 3012 Bern, Switzerland; 4 Oeschger Centre for Climate Change Research, University of Bern, Falkenplatz 16, 3012 Bern, Switzerland; 5 Georgina Mace Centre for the Living Planet, Department of Life Sciences, Imperial College London, Silwood Park Campus, Buckhurst Road, Ascot, SL5 7PY UK; 6 Forestry Research Institute of Ghana, Council for Scientific and Industrial Research, Kumasi, Ghana; 7 Department of Natural Resources Management, CSIR College of Science and Technology, Kumasi, Ghana; 8 AMAP (Botanique et Modelisation de l’Architecture des Plantes et des Végétations), CIRAD, CNRS, INRA, IRD, Université de Montpellier, Montpellier, France

**Keywords:** Tropical ecology, Tropical ecology

## Abstract

Tropical forests dominate terrestrial photosynthesis, yet there are major contradictions in our understanding due to a lack of field studies, especially outside the tropical Americas. A recent field study indicated that West African forests have among the highest forests gross primary productivity (GPP) yet observed, contradicting models that rank them lower than Amazonian forests. Here, we show possible reasons for this data-model mismatch. We found that biometric GPP measurements are on average 56.3% higher than multiple global GPP products at the study sites. The underestimation of GPP largely disappears when a standard photosynthesis model is informed by local field-measured values of (a) fractional absorbed photosynthetic radiation (fAPAR), and (b) photosynthetic traits. Remote sensing products systematically underestimate fAPAR (33.9% on average at study sites) due to cloud contamination issues. The study highlights the potential widespread underestimation of tropical forests GPP and carbon cycling and hints at the ways forward for model and input data improvement.

## Abstract (web summary)

This study finds that vegetation models commonly underestimate the productivity of West African forests, owing to bias in fractional absorbed photosynthetic radiation (fAPAR) and leaf level photosynthetic capacities.

## Introduction

Carbon exchanges between terrestrial ecosystems, especially tropical forests, and the atmosphere are a major element of the global carbon cycle. As the world’s most productive terrestrial ecosystems^1^, tropical forests have been estimated to account for around 44% of global forest biomass and 43% of global gross primary production (GPP)^2,3^. Nonetheless, confidence in estimates of tropical forest productivity remains low^4,5^. There is still a large variation among models regarding the magnitude and spatial pattern of tropical GPP^6–8^. Multiple global-scale forest GPP studies have indicated that tropical forests have larger uncertainty compared to other biomes^9–12^. This is likely an inevitable consequence of the paucity of carbon cycling data in the tropics relative to temperate regions^13–16^. For instance, many previous studies found particularly large GPP data-model discrepancies for tropical forests between flux towers and models (including satellite GPP products)^16–19^, but investigation into the causes of discrepancy was not attempted due to the lack of in situ auxiliary measurements of plant traits and forest characteristics^20^. The large unresolved data-model discrepancy suggests fundamental challenges in our understanding of tropical forest productivity and its geography.

The uncertainty in estimates of tropical forest productivity is particularly large in West Africa^21^. The first field quantification of GPP in African forests (termed ‘biometric GPP’) reported possibly the highest GPP value recorded in intact forests, which however, are underestimated by about 60% in both the MODIS and FLUXCOM GPP products, two widely used global maps of tropical forest productivity^22^. Since GPP is the source of carbon and energy for terrestrial ecosystems in Dynamic global vegetation models (DGVMs)^23^, the data-model discrepancy in GPP propagates into NPP (Fig. S5) and possibly other downstream variables. It has not yet been explained, from modellers’ perspective, why this area has a GPP higher than simulations. As models are designed based on current ecological theory^24^, such a large discrepancy of multiple sites signals a lack of physiological understanding or poor parameterisation of forest physiology.

In this work, we set out to explain the high productivity of West African forests using photosynthesis models and investigate the reasons behind the data-model discrepancy, with a view to provide information to modellers working on assessing and mapping the productivity of forests globally. The investigation follows three objectives (Table 1). Objective (1): We first compare the biometric GPP to multiple models and satellite-based products, to quantify the data-model discrepancy. Objective (2): We investigate whether the field-observed high productivity is consistent with the leaf photosynthetic traits and other field measurements that were commonly simulated by models. This investigation is made possible by extensive field measurements of environmental variables, plant traits, and carbon fluxes at the study sites^25–28^. Objective (3): We attempt to account for the data-model discrepancy of MODIS and Pmodel (Table 2) and explore key responsible parameters in DGVMs. This is done by substituting model parameters with field measurements, according to several hypotheses listed below as to the cause of this discrepancy.

**Table 1:** Study objectives, hypotheses and testing datasets

Objectives	Data	Figure
1, quantify the data-model discrepancy	Pmodel_null, MODIS, Biometric, FLUXCOM, TRENDY	Fig. 2
2, consistency between leaf photosynthetic traits and biometric GPP	Compare Pmodel_PfL to Biometric	Fig. 3
3, Possible sources of data-model discrepancy (including 4 hypotheses)		
Hypothesis 1 LUE and photosynthetic capacity	Compare Pmodel_PfL to Pmodel_Pf; Compare Pmodel_null to MODIS	Fig. 3; Figure S6
Hypothesis 2 fAPAR	Compare Pmodel_Pf to Pmodel_P	Fig. 3
Hypothesis 3 Plant functional types	Compare TRENDY Forest-only GPP to whole-pixel GPP	Fig. S4
Hypothesis 4 Climate variables	Compare ERA5-Land, MERRA-2 (by GMAO), CRU-JRA to field measurements	Fig. S3

**Table 2:** Sources of gross primary production (GPP)

GPP	Main features	Group
Biometric	Measuring Each GPP component (e.g. leaf NPP, stem respiration) in the field.	Field works
TRENDY	Global dynamic vegetation models that used environmental variables (but not fAPAR) as inputs.	Model
Pmodel	a light use efficiency model, which was used to design multiple experiments to test hypotheses for Objective 3. The experiments include Pmodel_null, Pmodel_PfL, Pmodel_Pf, and Pmodel_P (see Fig. 3).	Model
MODIS	MODIS GPP is a light use efficiency model that calculates GPP using satellite observed fAPAR.	Observation based products
FLUXCOM	By upscaling carbon fluxes observed by eddy covariance towers.	Observation based products

Most vegetation models and global GPP products calculate GPP using key inputs including (1) light use efficiency (LUE), a key parameter of MODIS-GPP and Pmodel. LUE could not be directly measured but could be derived from field-measured photosynthetic capacity^29^ (Supplementary method). Pmodel^29^ and most DGVMs use photosynthetic capacity to calculate GPP^30^; (2) fraction of absorbed photosynthetically active radiation (fAPAR), a key parameter of MODIS-GPP and Pmodel. Models typically simulate LAI and then express fAPAR as a function of LAI and canopy extinction coefficient; (3) plant functional types (PFTs) classification; (4) climate variables, including temperature, relative humidity and photosynthetic photon flux density (PPFD), often calculated from incoming shortwave radiation. For models these are retrieved from global climate data products.

Therefore, for objective (3), we hypothesise that the large data-model discrepancy for GPP could stem from inaccuracies in one or more of these key input variables: (Hypothesis 1) incorrect LUE, or photosynthetic capacity; (Hypothesis 2) incorrect fAPAR; or (Hypothesis 3) inappropriate assignment of plants functional types; or (Hypothesis 4) climate variables. Multiple experiments are designed to test each hypothesis (Table 1, see Methods for detailed experiment settings).

Overall, we identify key areas for model improvement and also provide a trait-based explanation for the high GPP observed in West Africa.

## Results

### Intercomparison of GPP estimates

**Fig. 1:**
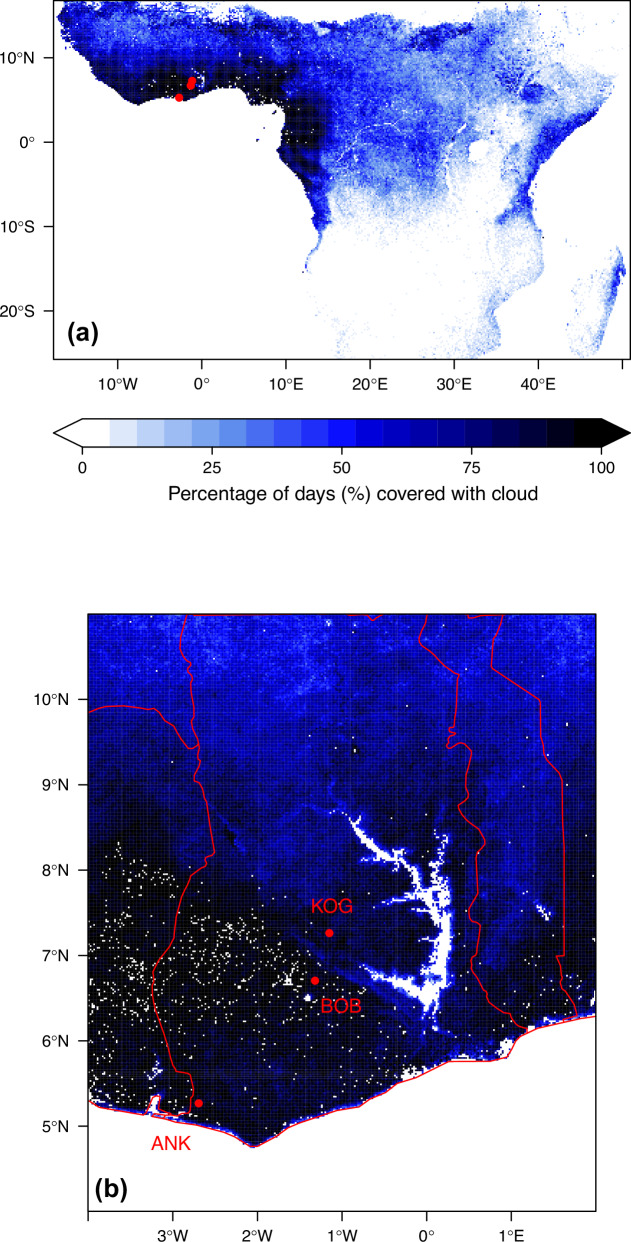
Map of the three study sites in West Africa. Panel (**a**) displays a regional view of Africa, and panel (**b**) zooms in on Ghana. Blue scales show the percentage of days contaminated by clouds during the cloudy season (August and September), which is the percentage of MODIS data marked as ’01 significant clouds present’ and ’10 mixed clouds present). Each red dot denotes a site. Each site contains multiple one-hectare plots.

**Fig. 2:**
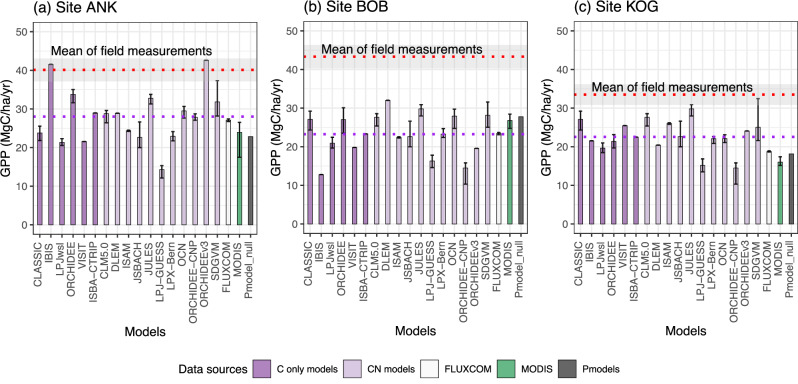
Intercomparison of gross primary productivity (GPP, MgC/ha/year) from various independent sources. The figure contains study sites (**a**) Ankasa, (**b**) Bobiri and (**c**) Kogaye. The red dotted lines denote in situ biometric GPP, as a mean of multiple one-hectare plots (Table S3); measurements are taken spanning 2011 to 2016^22,25^. The grey areas denote measurement uncertainty, not standard error. The uncertainty is calculated through error propagation. For GPP models and products, we calculate mean annual GPP from 2011 to 2016. Bars denote Carbon-only models (dark purple), Carbon-Nitrogen coupled models (light purple), FLUXCOM (white) and MODIS (green). The error bars are maximum/minimum mean annual GPP during the study period. The purple dotted line denotes all TRENDY models (purple bar) average. For TRENDY models that reported GPP per plant functional types, we display forests-only GPP (the potential GPP if the grid cell is full of forests). Otherwise, we display whole gridcell GPP. See Fig. S4 for a comparison between forest-only GPP and whole gridcell GPP. Source data are provided as a Source Data file.

Each study site represents a major forest type of West African forests (Fig. 1). For all study sites, considerable discrepancies in GPP are found among DGVMs, FLUXCOM, MODIS, Pmodel, and biometric measurements, with a pattern that is consistent from site to site (Fig. 2). At each site, biometric measured GPP exceeds methods GPP and global GPP products. Overall, the DGVMs’ average GPP is higher than FLUXCOM, which in turn is higher than MODIS GPP. The discrepancy between biometric and MODIS GPP is about 20 MgC/ha/year regardless of sites. The largest data-model discrepancy between DGVMs and biometric GPP is seen at BOB (22.8 versus 43.3 MgC/ha/year). At BOB, the DGVM average, FLUXCOM, and MODIS report almost equal GPP – but all less than the measured GPP. There are substantial disagreements between different DGVMs; for example, at ANK, the DGVMs simulated GPP ranged from 14.3 to 42.6 (biometric GPP at 40.1) MgC/ha/year.

### The match between trait-based GPP and biometric GPP

**Fig. 3:**
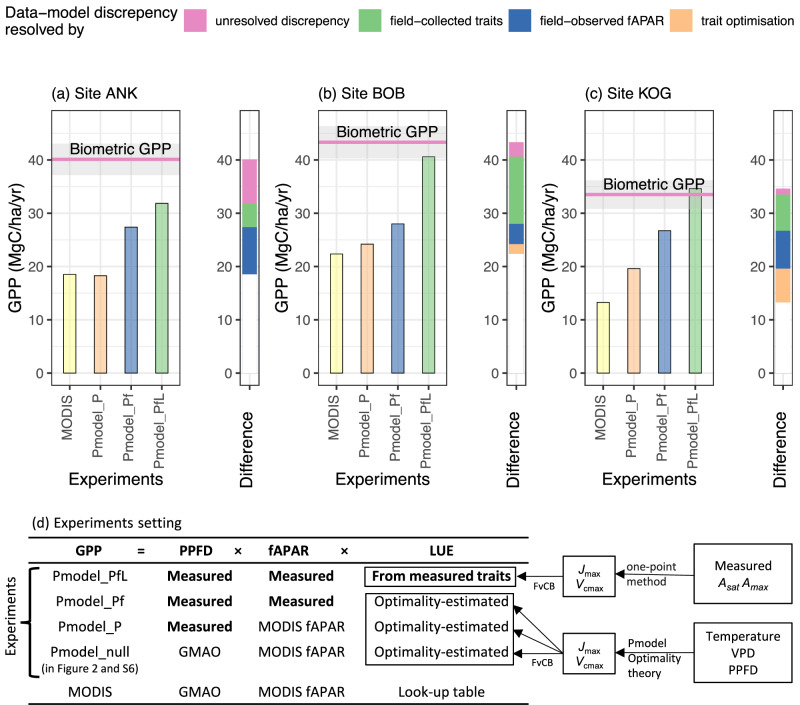
Partitioning of GPP data-model discrepancy, by comparing different experiments. Results are shown for study sites (**a**) Ankasa, (**b**) Bobiri and (**c**) Kogaye. Experiments design is shown in (**d**). The left panel displays field-based biometric GPP (pink line with grey zone showing uncertainty) and multiple GPP experiments (bars). Pmodel_PfL, Pmodel_Pf and Pmodel_P are GPP experiments which are all simulated using Pmodel but with different inputs. Both Pmodel and MODIS GPP were calculated from Eq. (1), enabling direct comparison. The right panel, a ‘Diff’ bar, illustrates the difference in GPP between experiments, which represents the sources of GPP data-model discrepancy. For example, the GPP difference between Pmodel_Pf and Pmodel_P experiments is caused solely by the difference in input variables - fAPAR. Therefore, the difference between them (blue) is GPP data-model discrepancy resolved by fAPAR. The difference (orange) between Pmodel_P and MODIS is GPP data-model discrepancy resolved by optimality-estimated LUE and PPFD (but mostly LUE, Fig. S6). The difference (green) between Pmodel_PfL and Pmodel_Pf is GPP data-model discrepancy caused by the difference between measured LUE and optimality-based LUE. MODIS fAPAR and GPP are from MOD15A2H and MOD17A2H excluding data marked as ‘01-Significant clouds were present’. Source data are provided as a Source Data file.

Using field observed PPFD, fAPAR, and photosynthetic traits as input, Pmodel could estimate a GPP (i.e., experiment Pmodel_PfL) slightly lower but still within the uncertainty range of field biometric GPP at BOB and KOG sites. At the ANK site, Pmodel_PfL greatly reduces the data-model discrepancy but remains lower than biometric GPP (Fig. 3). Nonetheless, the slight mismatch at ANK does not undermine further investigation at ANK because there are more dominant factors contributing to the data-model discrepancy, explained below.

### Photosynthetic trait and fAPAR

It was hypothesised that GPP data-model discrepancy could stem from inaccuracies in (Hypothesis 1) LUE, or photosynthetic capacity; (Hypothesis 2) fAPAR. Photosynthetic capacity estimated by the Pmodel is lower, although close to field measured photosynthetic capacity (Fig. S2) implying underestimated LUE. As a consequence, Pmodel_Pf is lower than Pmodel_PfL consistently at all sites (Fig. 3).

**Fig. 4:**
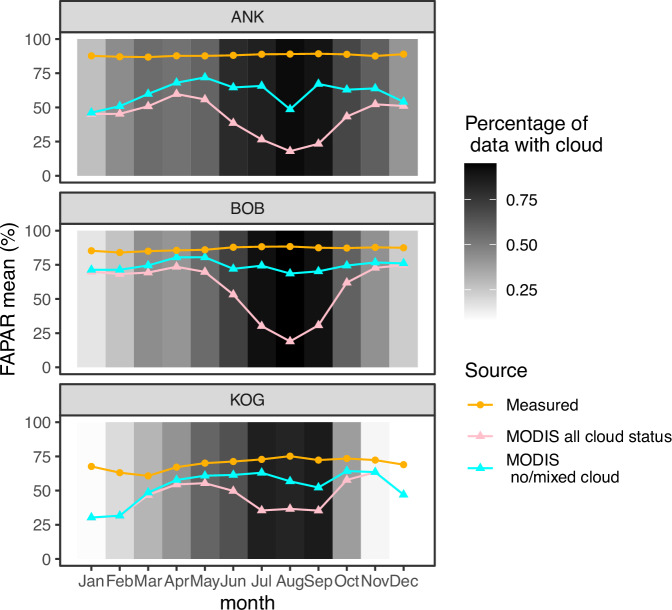
The seasonal variation of the fraction of absorbed photosynthetically active radiation (fAPAR). The figure presented data retrieved from MODIS MOD15A2Hv006 (triangle) and in situ measured using hemispherical photography (dot, orange line), at the three study sites (ANK, BOB and KOG), as a monthly mean across 2011 to 2016. The figure shows all MODIS data regardless of the quality flag (Pink line); MODIS fAPAR with cloud contamination control by selecting data marked as 00-no cloud and 10-mixed cloud presents (Blue line). Selecting 00-no cloud only will result in too few data to be presented in the wet season. Grey scale shows the percentage of records contaminated by clouds. Source data are provided as a Source Data file.

GPP is substantially underestimated when in situ fAPAR is replaced with MODIS fAPAR, resulting in a considerable difference between Pmodel_Pf GPP and Pmodel_P at any site. Especially at ANK, fAPAR is found to be the largest contributor to GPP data-model discrepancy. A further investigation into MODIS fAPAR (Fig. 4) shows that during the rainy season, MODIS fAPAR decreases along with increasing cloud cover (up to 90% of pixels), whereas field fAPAR remains fairly constant during the year. Filtering fAPAR values according to cloudiness or gap-filling of bad data points would remove most of the values from the rainy season, leading to an underestimated mean annual fAPAR. The above issue affects a major proportion of the African tropical forest, and likely many areas of the tropics (Fig. 1).

By comparing to Pmodel_null, we showed that GMAO PPFD lead to overestimation of GPP at site ANK and BOB, but leads to slight underestimation of GPP at site KOG (Fig. S6). This is associated with the low resolution of GMAO PPFD. Using a higher resolution PPFD product (e.g. ERA5, Fig. S3) could alleviate this issue. Thus, we conclude that PPFD could not explain the underestimation of GPP at the study sites. At the ANK site, MODIS and Pmodel_P GPP are almost identical (Fig. 3a), but at BOB and KOG, MODIS is lower than Pmodel_P GPP. This portion of data-model discrepancy could be resolved by using the optimality-based LUE other than MODIS LUE (from the look-up table) (Fig. S6). This portion of data-model discrepancy could not be resolved by PPFD as explained above. Although MODIS LUE is not publicly available, the comparison between Pmodel_null and MODIS suggests that optimality theory tends to estimate an LUE higher than MODIS LUE. In short, Hypotheses 1 and 2 are accepted.

### Scaling and plant functional type

Hypothesis 3 suggests that the GPP data-model discrepancy originated from inappropriate assignments of plant functional types. At BOB the entire forest fragment is smaller than a single grid cell in a DGVM but is hundreds of times larger than a MODIS grid cell (scales illustrated in Fig. S1). MODIS GPP estimates that the surrounding area (a composite of savanna and cocoa farms) has only half the productivity of the forests. Therefore, the mean of the whole map (19.6 MgC/ha/year) appears to be smaller than that of the forests (23 MgC/ha/year). This is not the case for TRENDY, which simulated a comparable value between forests-only and whole grid cell GPP (Fig. S4) (i.e. similar simulated GPP between forests and C4 grass). There are no field studies to our knowledge comparing cocoa farm GPP to adjacent forests GPP.

For all the study sites, most TRENDY models correctly simulate the grid cell as a composite of ‘forest’ and ‘C4 grass’, although the fraction of forests varies considerably among models (Table S1). Given that the ‘forest-only’ GPP of TRENDY and MODIS are much lower than biometric GPP (Figs. 2; S4; S1) we conclude that the GPP data-model discrepancy between TRENDY and biometric GPP cannot be explained by scaling or PFT issues (rejecting hypothesis 3). Besides, please note that TRENDY S2 keeps the land cover state fixed to its pre-industrial state of 1700 and thus the proportion of C4 grass (Table S1) is temporarily invariant and could be smaller than a land cover dynamic simulation (e.g. MODIS replies on satellite imageries that incorporates real-time land cover change).

### Data-model consistency in climate variables

Hypothesis 4 suggests that the GPP data-model discrepancy originated from climate variables. The data-model differences we found for temperature, relative humidity, PPFD and c_i_/c_a_ were too small to explain the GPP data-model discrepancy (Fig. S3), thus rejecting hypothesis 4.

To sum up, the data-model discrepancy for GPP at the study sites mainly stems from: incorrect LUE, or photosynthetic capacity (Hypothesis 1 accepted); and incorrect fAPAR (Hypothesis 2 accepted); not inappropriate assignment of plants functional types (Hypothesis 3 rejected); nor climate variables (Hypothesis 4 rejected).

## Discussion

### Main sources of GPP data-model discrepancy

We not only demonstrate a significant data-model discrepancy in West African forest GPP but also reveal the main sources of this discrepancy. At the Ankasa wet rainforest site, we found that underestimation of fAPAR, likely because of cloud contamination, is the major contributor to GPP data-model discrepancy. At the Bobiri (semideciduous) and Kogyae (dry forest) sites, too low values of photosynthetic traits (leading to a bias in light use efficiency) are the primary source of GPP data-model discrepancy, which can be partly explained using optimality theory predictions. Additionally, fAPAR accounts for a large proportion of GPP data-model discrepancy at KOG but not at BOB. Through this analysis we are able to fully account for the data-model discrepancy at KOG and BOB, but leave part of the data-model discrepancy unresolved (20.5% of biometric GPP) at ANK, which may be caused by inaccurate traits measurements, photosynthesis model assumption (e.g. the big leaf assumption) or possible bias in field measurements of the biometric GPP (e.g. the upscaling of leaf and stem respiration; the estimation of roots exudation)^31^.

### Why do models underestimate GPP in West African tropical forests?

Focusing on three sites (comprising 14 one-hectare plots) in Ghana, we have utilised a recent in situ quantification of African forest GPP and conducted a systematic data-model comparison. Despite the uncertainty associated with biometric GPP measurements, it is more likely that FLUXCOM, MODIS, TRENDY models, and Pmodel_null underestimate West African forest productivity than that the biometric GPP measurements overestimate it. This is because (1) the biometric GPP is an average across multiple plots spanning several years. To our knowledge, there are no other biometric GPP measurements in the study region, but the high GPP is supported by another field study reporting very high forests biometric NPP^32^; (2) from a photosynthesis traits perspective, West African seasonally dry forests are characterised by a high CO_2_ assimilation rate or high photosynthetic capacity, in comparison to wet-evergreen tropical forests studied in other continents, predominately in Amazonia^27,33,34^; Pmodel could simulate a GPP close to biometric GPP if informed by field-measured traits (Fig. 3), which signals a broad consistency between the high GPP and observed photosynthetic traits. (3) the cause of the models’ underestimation can be well explained by errors in modelled LUE and satellite-derived fAPAR (Fig. 3). (4) There is only one flux tower for West African forests (site GH-ank at the Ankasa site), which reports three years of GPP varying substantially^19^. The tower reported 2011 annual GPP at 36.06 MgC/ha/year, but the tower only operated for half-year in 2012 and 2014 with estimated annual GPP at 22.02 and 26.1 respectively (FLUXNET2015^35^, variable GPP_NT_CUT_REF) (Fig. S8). In comparison, the biometric GPP is 40.11 and Pmodel_PfL is 31.85. Therefore, the ‘true’ GPP of site ANK appears to be more likely to situate around 36 MgC/ha/year with biometric GPP overestimating, and the ‘unresolved data-model discrepancy’ at site ANK (Fig. 3) is probably due to bias in the biometric GPP. Many tropical flux towers face logical or technical issues^36,37^, and challenging subcanopy CO_2_ storage estimation^38,39^. For example, the road leading to site ANK is seasonally inundated (Photos in Supplementary information) when the study site can only be accessed on foot. During FLUXCOM extrapolation, the machine learning algorithm would not receive information about BOB’s higher photosynthetic capacity and would simply predict BOB is less productive than ANK (opposite the observed GPP pattern) due to the lower precipitation received at BOB (Table S3).

Some degree of model underestimation of GPP may even be a pan-tropical feature. Intercomparison of GPP and NPP in previous studies^40,41^ has also revealed data-model discrepancies for Amazonian lowland forests. In this study, we found that the data-model discrepancy was due to fAPAR and photosynthetic capacity. The cloudiness issue, leading to low satellite-based fAPAR, has been described as a pan-tropical phenomenon for many remotely sensed products^42–45^. Moreover, cloud-free fAPAR (Fig. 4) also underestimate fAPAR thus there are issues beyond cloud contamination. Bias in MODIS fAPAR (or LAI) will inevitably cascade into models or analyses that use MODIS fAPAR as a predictor, including FLUXCOM. Large GPP discrepancy between global flux towers and MODIS are reported, which was found associated with fAPAR and LUE not cliamte variables^19^. Many tropical rainforests have high persistent cloud cover compared to other forests and therefore may be disproportionally affected by cloud contamination^43,45^.

The LUE (or photosynthetic capacity) discrepancy is strong and consistent with previous literature, since a previous study found higher *A*_sat_ and *A*_max_ values in West African species than in Amazonian ones^34^. Higher *A*_sat_ and *A*_max_ are found in BOB and KOG (drier sites), but not ANK (the wet evergreen forest on poor soils more similar to much of lowland Amazonia)^22,27^, making LUE the dominating source of GPP bias in BOB and KOG but less dominant in ANK. The high photosynthetic capacity in semi-arid forest or savanna is consistent with other field studies in West Africa^33^. *V*_cmax_ inferred from remotely sensed leaf chlorophyll data and *V*_cmax_ predicted by the P model both show exceptionally high values in West Africa and parts of India – substantially higher than in Amazonia or SE Asia^46,47^. These seasonal forests on more fertile soils may have photosynthesis rates optimised to high light, temperature and VPD (also see field study^48^). This substantial spatial variability of *V*_cmax_ has not been incorporated in most TRENDY models^30,49^ which could lead to underestimation of GPP at such regions. Traditionally, *V*_cmax_ is a PFT-specific constant. If the true *V*_cmax_ differs among sites due to nutrient availability, then models can only embody the dynamics with varying PFT fractions. Some models do simulate *V*_cmax_ as a function of leaf nitrogen, in which case they could, at least in theory, reproduce those patterns. Nonetheless, leaf nitrogen may be simulated also with PFT-specific parameters and the N cycle has its own set of challenges^30^. Thus, further modelling study is needed to carefully consider the high *V*_cmax_ at this region.

We note that *V*_cmax_ behind TRENDY simulation is not directly retrievable and thus we could not conduct direct data-model comparison for *V*_cmax_ but discuss and compare to the PFT-specific values recalculated following models’ equations^50^ (Table S2). As shown in Table S2, two TRENDY models (OCN and ORCHIDEE) indeed underestimate *V*_cmax_. IBIS substantially overestimates *V*_cmax_ which results in its high GPP at site ANK. However, JULES, CLASSIC, CLM5.0 and JSBACH have similar or higher *V*_cmax_ but still underestimate GPP, which implies that there are other factors contributing to the GPP underestimation. Moreover, the substantial inter-model disagreement shown in Table S2 is alarming, with *V*_cmax_ for evergreen forest vary from 18 to 163 μmol m^−2^ s^−1^ while field measurements at our study sites are around 30 μmol m^−2^ s^−1^^51^. Model parameterisations of *V*_cmax_ for C4 grass also vary substantially and appear to be lower or higher than forests depending on models, which may affect some models that have C4 grass as main plant functional types at the study sites, for example, CLM5.0. Further, although *V*_cmax_ model parameters may differ from measurements at the study sites, the values may still be appropriate at a larger scale, as in-situ *V*_cmax_ measurement values differ considerably among sites. The wide range of *V*_cmax_ actually reflects substantial variation in global traits databases^46^, where further data-model benchmarking study is suggested. Most TRENDY models embedded an exponential equation linking fAPAR with LAI. In other words, GPP is positively influenced by *V*_cmax_ and LAI. At study site KOG, most models overestimate LAI (Figure S7). Thus, LAI would not explain the underestimation of GPP at this site. At study sites BOB and ANK however, models averaged LAI is very close to field measurements although with large inter-model variation, suggesting that the key parameters accounting for GPP underestimation here should vary among models. To sum up, the investigation into the cause of DGVMs underestimating GPP is not straightforward. The parameterisation of *V*_cmax_ and LAI is one of the reasons for some models but there are likely other contributing factors that require future studies.

### The importance of field evidence in productivity estimation

The availability of comprehensive field measurements allows us to trace and quantify the sources of data-model discrepancy in the GPP of West African forests. We find that fAPAR and LUE (or photosynthetic capacities) are the dominant sources of data-model discrepancy, rather than model structure or climate variables. These findings reflect the lack of field measurements of West African forest photosynthetic traits and leaf area index (or fAPAR), while the environmental variables of the study regions are better represented. FLUXCOM also struggles with the only flux tower for West African forests that reported three years of patchy data. Beyond pointing out the sources of data-model discrepancy, this study also highlights that such issues could be fixed if models were better informed with field-measured fAPAR and LUE derived from measured traits, or more generally if better maps of canopy traits are applied^52^. The model simulated GPP could be improved with predicted LUE by optimality theory (Fig. 3), which highlights the importance of using ‘trait-based approaches’ parameters instead of PFTs prescription^30,53^. This study thus suggests that providing models with ample field evidence and ensuring strong fidelity to field measurements is critical in improving current carbon cycle simulations^54^.

### Implication for carbon cycle modelling of West African forests

Researchers studying tropical forest functioning should exercise caution when using satellite-based products subject to cloud contamination, as they are strongly compromised during the rainy season in this region due to high cloudiness (Fig. 1). Moreover, both cloud-free MODIS GPP (Fig. 3) and fAPAR (Fig. 4) are lower than field measurements, suggesting room for improvement beyond cloud contamination. Additionally, West African forests are highly fragmented^55^ (Fig. S1), so future studies across scales are suggested to check plant functional types assignment (Fig. S4)^32^.

The study has shown that the Pmodel has advanced prediction of c_i_/c_a_, *V*_cmax_ and *J*_max_ – essential parameters for the FvCB model. The data also support the coordination of the Rubiscio- and electron transport-limited photosynthetic rates, *A*_C_ and *A*_J_, which is one of the optimality principles underlying the Pmodel (Fig. S2). However, the reasons why almost all TRENDY models underestimate West African GPP has not been fully elucidated. The investigation of TRENDY models’ LAI and *V*_cmax_ suggests that these models do not share the same cause of the data-model discrepancy. While much criticism of TRENDY models has centred on likely inaccurate characterisations of plant functional types^30^, our study suggests that this is unlikely to be the cause of GPP bias at the study sites. Further, we identified *V*_cmax_ as a key factor. Numerous models calculate *V*_cmax_ from leaf nitrogen^30^ but nitrogen-coupled models do not contrast with carbon-only models at the study site (Fig. 2), suggesting that the nitrogen-GPP relationship still has room for improvement in future modelling studies. More transparency in models’ input data (e.g. leaf nitrogen content) and parameterization (e.g. *V*_cmax_) is necessary for further investigation. Although it is challenging to unbox each model and investigate their *V*_cmax_, *J*_max_ or LUE, our analysis suggests that the underestimation of GPP is associated with photosynthetic capacities, especially at site BOB and KOG (Fig. 2). Allowing *V*_cmax_, *J*_max_ or LUE to acclimate to brighter and drier environments should improve the simulation of GPP at semi-arid forests and savanna^56^.

In conclusion, the study not only reveals an underestimation of West African forest productivity but also explores why this underestimation occurs. The unique data-model comparison approach proposed in this study may also have wider potential as it successfully (1) shows consistency between field-measured photosynthetic traits and biometric GPP; (2) identify likely sources of data-model discrepancy by designing multiple experiments with a minimal photosynthesis model (Pmodel). The study also demonstrates that to gain insight, thorough field measurements of forest plots are valuable and the application of simple models (that can be easily understood and tuned) is key for ecological hypothesis testing. As this study is limited in terms of spatial cover, we encourage future research in this region, in particular drier African ecosystems where C4 grasses are abundant with more LUE but less cloudiness (Fig. 1)^57^. We also acknowledge that models are intended for global simulation and thus our site scales study does not serve as models benchmarking but approaches to improve model simulation. Nonetheless, given the broad consistency in our results displayed across the study sites, we expect that models’ carbon cycle simulation in West African region could be substantially improved by simulating a higher GPP across West African tropical forests. It is possible, indeed likely, that such model-data discrepancies are a more general feature of tropical forests. This requires further detailed comparison between biometric field measurements and model predictions, with the approach outlined here offering a valuable approach for such a pantropical analysis.

## Methods

### Study sites and field measurements

The three study sites span a wet-to-dry rainfall gradient in Ghana from evergreen rainforest at site Ankasa (ANK) with a mean annual precipitation of 2050 mm, to semi-deciduous forest at site Bobiri (BOB) with 1500 mm, and a dry forest and mesic savanna matrix at site Kogyae (KOG) with 1200 mm (Fig. 1). Each study site represents a major forest type of West African forests. See Table S3 for site environmental data.

Biometric GPP was measured in the field using the Global Ecosystem Monitoring protocol. Each component of GPP (e.g. canopy productivity, stem respiration, fine root productivity) is measured separately with common technique. For example, canopy productivity is measured using litterfall trap, and stem respiration is measured by attaching a CO_2_ Gas Analyzer (PPsystems EGM4) to stem surface. Biometric GPP was then calculated as a sum of each component^28^ and the field practice at our study sites is described in detail in a previous study^22^. Biometric GPP was originally quantified at the plot scale. There are three one-hectare plots at Ankasa, six plots at Bobiri, and five plots at Kogyae. This study was conducted at site rather than plot scale because one-hectare plots at the same site share almost identical climate variables and fall into one model grid cell. Specifically, a grid cell of TRENDY models and FLUXCOM covers approximately 50 × 50 km, or 0.5° × 0.5°. Nonetheless plots at the same site fall into different grid cell of MODIS due to higher resolution (500 ×500 m) (Figure S1). We, therefore, calculated a mean biometric GPP for each site as an average across plots. We did not calculate a standard error. The uncertainty in Biometric GPP (Fig. 2) represents measurements error (mostly systematic error) as calculated by error propagation from each GPP component, instead of spatial variation of GPP. See this study^22^ for more data and error propagation associated with the study sites.

The fraction of absorbed photosynthetically active radiation, fAPAR, was obtained using hemispherical photography, taken each month in each plot between 2012 and 2017. Photos were processed using machine learning-based software ‘*ilastik*’^58^ for pixel classification and CANEYE^59^ for fAPAR calculations (see Supplementary method for details).

In this study, we also need field-measured community-weighted mean light-saturated photosynthetic rate (*A*_sat_), light- and CO_2_-saturated photosynthetic rate (*A*_max_), to calculate a trait-based LUE and trait-based GPP (specific experiment explained in Section 2.4). We derived the maximum carboxylation rate (*V*_cmax_) and the maximum electron transport rate (*J*_max_) from each field measured *A*_sat_ and *A*_max_ at growth temperature^60^ using R package ‘plantecophys’^61,62^. We used these *V*_cmax_ and *J*_max_ in one of the Pmodel experiments for Objective 3. However, the *J*_*max*_ limitation equation in ‘plantecophys’ is different to that in ‘*rpmodel*’ (an R package used to conduct Pmodel experiments), so we modified the *J*_*max*_ equation in ‘*plantecophys*’ (see Supplementary Methods). Measurements of *A*_sat_ and *A*_max_ were made every three months from 2014 to 2016 to cover both wet and dry seasons. To measure *A*_sat_ and *A*_max_, we used an open-flow gas exchange system (LI-6400XT, Li-Cor Inc., Lincoln, NE, USA). To ensure a proper representation of the forest stand, we sampled tree species that constituted approximately 80% of the plot basal area. For each species, we selected three mature and canopy emergent trees, and cut one fully sunlit and one shaded branch per tree using a single rope climbing technique. We immediately placed and recut the cut branch under water, and measured the maximum rate of net CO_2_ assimilation at 400 ppm CO_2_ (*A*_sat_) and 2000 ppm CO_2_ (*A*_max_) on three leaves per branch. The PPFD was set to 2000 μmol m^–2^ s^–1^ and block temperature was kept constant at 30 °C. Although we measured both shade and sun leaves, we used sun leaves only in this study, consistent with common practice in field studies of photosynthetic traits^63^. Besides, we used above-canopy PPFD as model input and only sun leaves acclimate to this level of PPFD; shade leaves acclimate to darker environments and have consistently lower *A*_sat_ than sun leaves^64^. The ratio of leaf internal CO_2_ to eternal CO_2_ (c_i_/c_a_) was estimated from leaf δ^13^C measurements. We initially estimated the difference between the leaf stable isotope ratio and the atmospheric stable isotope ratio at that place and time (Δ^13^C) from δ^13^C, using the method described by a previous study^65^. Subsequently, we calculated c_i_/c_a_ from Δ^13^C using equation 11 in a previous study^60^. We compared measured c_i_/c_a_ to Pmodel estimated c_i_/c_a_ (Fig. S3).

### MODIS fAPAR and GPP

Using Google Earth Engine, we retrieved MODIS GPP from the collection MOD17A2H and retrieved MODIS fAPAR from MOD15A2H from 2011 to 2016. We extracted GPP and fAPAR of the 14 plots using their coordinates and calculated annual (Fig. 3) and monthly mean values (Fig. 4) per site. To remove cloud-contaminated GPP and fAPAR, we selected only data flagged as ‘Significant clouds NOT present (clear)’ or ‘Mixed cloud present on pixel’ provided in band ‘faparlaiQC’ and ‘Psn_QC_500m’. In rain season, almost no data is marked as ‘Significant clouds NOT present (clear)’ (Figs. 1 and 4). Since MODIS GPP and fAPAR share the same data flag, the above approach ensures that the fAPAR used in Pmodel experiments is identical to that used in MODIS GPP (Fig. 3), ensuring a fair comparison between them. MOD17A3HGF product contains MODIS GPP and NPP after gap-filling MOD17A2H. We used GPP and NPP from MOD17A3HGF to compare with other models and GPP products (Fig. 2, Fig. S1).

### FLUXCOM and TRENDY GPP

We chose the ‘RS_METEO’ version of FLUXCOM because the magnitude of GPP in this version does not involve uncertainty from MODIS fAPAR, which makes the comparison between FLUXCOM and MODIS GPP more independent. Specifically, we used “GPP.RS_METEO.FP-ALL.MLM-ALL.METEO-ERA5.720_360”. For TRENDY, we analysed the model outputs in version 9^66^ under the S2 protocol, in which climate and CO_2_ change while the land cover state is fixed to its pre-industrial state of 1700. Models are classified into nitrogen-carbon coupled models and carbon only models for readers convenience. Note that models still include ‘C4 crop land’ as one of the plant functional types (PFTs), but the cover of ‘C4 crop land’ is kept constant. We retrieved total GPP (sum of all PFTs) of the grid cell (variable ‘gpp’). We also used variables ‘gpppft’ and ‘landcoverfrac’ to calculate a forest-only GPP, which is the GPP that the model would have estimated if the whole grid cell were ‘forests’, including evergreen, deciduous and any other types of forests. For both TRENDY and FLUXCOM, we extracted the grid cells where the three study sites are located, as an average from 2011 to 2016.

### Experiment design for Objective 1

For Objective (1), we compared biometric GPP with values estimated by (a) the TRENDY ensemble of dynamic global vegetation models (DGVMs)^66^; (b) two data-driven products, FLUXCOM^67^, and MODIS^68^, and (c) an optimality-based model (Pmodel v1.0)^29^. The above choices are widely used models and GPP products that applied distinctly different approaches to calculate GPP^16^(Table 2).

The TRENDY v9 S2 ensemble is a collection of 15 DGVMs that calculate functional aspects of vegetation, including fAPAR or leaf area index (LAI), metrics that determine light absorption from environmental variables, without using any remotely sensed data as input. Most of these models calculate leaf-level photosynthesis via the Farquhar-von Caemmerer-Berry (FvCB) photosynthesis model^69^, which requires specification of several photosynthetic traits: *V*_cmax_, *J*_max_, and parameters of one or other of the semi-empirical schemes that are commonly used to estimate stomatal conductance (*g*_s_)^30^. Leaf-level photosynthesis is scaled up to the canopy, and thus to GPP, by methods that vary in complexity, but all depend on the modelled LAI or fAPAR.

FLUXCOM and MODIS, by contrast, are observation-based GPP products that do not depend on the FvCB model. FLUXCOM GPP is a machine learning application that uses eddy-covariance estimates of GPP from worldwide flux towers as the key input, combined with environmental covariates that include satellite-derived fAPAR, and shortwave radiation (closely related to PPFD). FLUXCOM used MODIS fAPAR, and shortwave radiation prepared by Japan Aerospace eXploration Agency (JAXA) using Terra MODIS data.

MODIS GPP^68^ is a light use efficiency model^70,71^ that calculates GPP as:

1 $${{\rm{GPP}}}={{\rm{fAPAR}}} \,*\, {{\rm{LUE}}} \,*\, {{\rm{PPFD}}}$$

where PPFD is sourced from Global Modelling and Assimilation Office (GMAO). The MODIS GPP algorithm calculates LUE as a prescribed (per biome) maximum light use efficiency, multiplied by reduction factors that are defined a priori as biome-specific functions of temperature and vapour pressure deficit.

In this analysis we also employ Pmodel^29^. The Pmodel, uniquely, is a LUE model (also using Eq. 1) but it calculates LUE based on the FvCB model, using optimality principles^72^ to determine the spatial and temporal variation in *V*_cmax_, *J*_max_ and the ratio (c_i_/c_a_) of leaf-internal to ambient CO_2_^29^. The c_i_/c_a_ ratio results from the combined effects of photosynthetic rate and stomatal conductance, which are co-regulated by plants. Leaf-level photosynthesis is scaled up to the canopy with the help of the big-leaf approximation^73^ and driven by satellite fAPAR data. The Pmodel thus combines the mechanistic basis of photosynthesis as represented in DGVMs with the simplicity of LUE models. The Pmodel also dispenses with the need to consider plant functional type (PFT) or biome distinctions (apart from the difference between C_3_ and C_4_ plants); the differences in photosynthetic traits among C_3_ PFTs are implicitly predicted as a consequence of their habitat preferences alone. The validity of these predictions has been supported by global-scale comparisons^74–76^.

### Experiment design for Objective 2

Before investigating the cause of the GPP data-model discrepancy (Objective 3), it is necessary to first investigate whether biometric GPP can be reproduced by the FvCB model (within Pmodel) fully informed by field-measured inputs – PPFD, fAPAR and photosynthetic capacities (Objective 2). Here we fed Pmodel with *V*_cmax_ *J*_max_ derived from field measured *A*_sat_ and *A*_max_ (Supplementary Method). Note that the ‘big leaf assumption’ is implied for all Pmodel simulation in this study. The above GPP experiment was called ‘Pmodel_PfL’. A match would indicate consistency between the field-measured canopy properties and the independent field biometric GPP.

If such a match is found between Pmodel_PfL GPP and biometric GPP, we can further investigate the cause of GPP data-model discrepancy by designing more experiments using Pmodel with different inputs (Table 1 and Fig. 3). The difference between Pmodel_PfL and biometric GPP is labelled as the ‘unresolved discrepancy’ in Fig. 3.

### Experiment design for objective 3

We elucidated four hypotheses for Objective (3) (investigate the cause of data-model discrepancy) (Table 1), which are tested with the following procedure:

To test Hypothesis 1 (incorrect LUE explains the mismatch), we used the Pmodel to predict photosynthetic capacity (Vcmax, Jmax) and LUE using optimality theory based on climate variables alone. The Pmodel then calculates a ‘Pmodel_Pf’ GPP using the above optimality-based predictions, combined with field-measured PPFD and fAPAR. As the only difference between ‘ Pmodel_Pf’ and ‘Pmodel_PfL’ GPP is in light use efficiency (derived either from optimality or from in situ measurements), we label the difference in GPP as ‘data-model discrepancy resolved by field traits’.

Next, the model calculates a ‘Pmodel_P’ GPP also using measured PPFD and the above optimality-theory predicted LUE, but with MODIS-derived fAPAR rather than in situ measured fAPAR as input. When ‘Pmodel_P’ GPP is compared to MODIS GPP, the difference originates from PPFD and LUE (predicted by optimality theory versus from MODIS lookup table). The differences in PPFD among GMAO (used by MODIS^68^), ERA5-Land and field measurements are trivial in explaining GPP bias (Fig. S3) and an intercomparison of climate products is out of the scope of this study. We compared ‘Pmodel_P’ and ‘Pmodel_null’ to show that PPFD could not explain the underestimation of GPP at the study sites (Fig. S6). Thus, for simplicity, the difference between ‘Pmodel_P’ GPP and MODIS GPP is labelled as ‘data-model discrepancy resolved by trait optimisation’.

To test Hypothesis 2 (incorrect fAPAR explains the mismatch), we compared ‘Pmodel_P’ to ‘Pmodel_Pf’. The only difference between these two is in fAPAR (in situ measurements versus MODIS). We referred to the difference as ‘data-model discrepancy resolved by fAPAR’.

Lastly, ‘Pmodel_null’ GPP is calculated using satellite PPFD, in which case Pmodel gets no information from field measurements, which is a fair comparison to other models (Fig. 2).

Equivalently to comparing GPP, one could directly compare *V*_cmax_, *J*_max_, fAPAR and LUE from multiple sources (some presented in Figs. S2 and S3). However, LUE used in MODIS GPP are not publicly available and were difficult to reproduce, so we did not include them. Since the data-model discrepancies in these variables all cascade into GPP, we focus on visualising the discrepancies in GPP in Fig. 3.

Hypothesis 3 (misclassification of land cover) is based on the fact that the GPP estimated from in situ measurements, which is the mean of data from several one-hectare plots, differs in scale from the GPP estimated by the TRENDY models, FLUXCOM and MODIS (as visualised in Fig. S1). West African forests are extremely fragmented, and some forest patches are smaller than the grid cell size of the models. For example, one of the study forests, Bobiri (BOB), is a forest fragment measuring only about 7 × 15 km (Fig. S1), surrounded by cocoa farmland. To illustrate this, we drew a map of MODIS GPP (with a resolution of 500 m) over the Bobiri site, covering an area similar to a single 0.5˚ x 0.5˚ grid cell of the TRENDY models. In some TRENDY models, each grid cell is a composite of multiple PFTs (often represented by different fractions in different models) and those models report GPP per PFT. Thus, we also show the PFT composition in those models of the study sites and compare ‘forest-only’ GPP to the grid cell average (Fig. S4). The ‘forest-only’ GPP may contain several types of forests (e.g. deciduous and evergreen) (Table S1). We checked each model documentation to ensure that the ‘forest-only’ GPP represents the potential GPP of the grid cell if the whole grid cell were forests. LPJ-GUESS was excluded due to its TRENDY documentation, which advises against using landcoverfrac to scale per-PFT data. In LPJ-GUESS, PFTs can overlap due to age-cohorts and understory vegetation, making it impossible to report PFT fluxes in PFT-m² and meet the requirement that the sum equals the land fraction of the grid cell. As a result, PFT fluxes are reported per m²-grid area, not per PFT-m². To calculate gridcell totals, one must sum PFT fluxes across all PFTs (e.g., gpp = sum(gpppft over all PFTs). Any scaling by *landcoverfrac is not meaningful for LPJ-GUESS.

### Data-model comparison for climate variables and c_i_/c_a_

For Objective 3 Hypothesis 4 (i.e. bias in climate variables explain the GPP data-model discrepancy), the following analysis was conducted. We compared temperature and relative humidity from local weather stations to products commonly used by vegetation models and GPP products. TRENDY simulation used CRU-JRA; MODIS GPP used GMAO products, whose latest version is MERRA-2. FLUXCOM reported results forced by different climate variables products and we chose ERA5-Land version. ERA5-Land, which has better resolution than the above, distinguishes climate condition of the study sites well. We chose ERA5-Land to calculate optimality-estimated c_i_/c_a_ via ‘*rpmodel*’. We also derive c_i_/c_a_ from leaf δ^13^C measurements, using the method described in ref. ^60^. This isotope-derived c_i_/c_a_ was compared to Pmodel-predicted c_i_/c_a_ (Fig. S3). PPFD in this study is calculated from surface incoming shortwave radiation, following^74^. Note that a previous data-model comparison of climate variables for tropical forest sites^77^ found ERA-interim outperform Climate Forecast System Reanalysis (CFSR), MERRA2 and the Japanese 55-year Reanalysis (JRA55) for Africa (including our study sites). As we found very small data-model discrepancy in climate variables (Fig. S3) at the study sites, to maintain consistency we used ERA5-Land temperature, vapour pressure and optimality-estimated c_i_/c_a_ for all Pmodel experiments. We avoid using field measured climate variables to inform Pmodel to ensure a fair comparison between Pmodel and other GPP products. The choice of PPFD dataset was specific to the experiment (see simulation schematic in Fig. 3).

## Supplementary information

Supplementary information

Description of Additional Supplementary Files

Supplementary Data 1

## Source data

Source Data

Peer Review File

## Data Availability

All data generated in this study have been deposited in the ‘figshare’ database under accession code **[**10.6084/m9.figshare.25431796**]**. The above includes the environmental data of the study sites. Models and satellite outputs are too large to be uploaded, which will be available upon request. Source data are provided with this paper.

## Code availability

All data generated in this study have been deposited in the ‘figshare’ database under accession code **[**10.6084/m9.figshare.25431796**]**. The above includes the environmental data of the study sites. Models and satellite outputs are too large to be uploaded, which will be available upon request. Source data are provided with this paper.
