# Supplementary material for: Why models underestimate West African tropical forest primary productivity
Source: Nat Commun. 2024 Nov 6;15:9574. doi: 10.1038/s41467-024-53949-0 (PMC11541734; doi:10.1038/s41467-024-53949-0)
Supplement: Supplementary file 1 — Supplementary information [file 41467_2024_53949_MOESM1_ESM.pdf]

Supplementary for

Why models underestimate West African tropical forest primary productivity

Huanyuan Zhang-Zheng[1,2]✉, Xiongjie Deng[1], Jesús Aguirre-Gutiérrez[1,2], Benjamin D. Stocker[3,4], Eleanor Thomson[1], Ruijie Ding[5], Stephen Adu-Bredu[6,7], Akwasi Duah-Gyamfi[6], Agne Gvozdevaite[1], Sam Moore[1], Imma Oliveras Menor[1,8], I. Colin Prentice[5]✉, Yadvinder Malhi[1,2]✉

✉ Corresponding Authors

(huanyuan.zhang@ouce.ox.ac.uk)

(c.prentice@imperial.ac.uk)

(yadvinder.malhi@ouce.ox.ac.uk)

1 Environmental Change Institute, School of Geography and the Environment, University of Oxford, Oxford, United Kingdom,

2 Leverhulme Centre for Nature Recovery, University of Oxford, UK

3 Institute of Geography, University of Bern, Hallerstrasse 12, 3012 Bern, Switzerland

4 Oeschger Centre for Climate Change Research, University of Bern, Falkenplatz 16, 3012 Bern, Switzerland

5 Georgina Mace Centre for the Living Planet, Department of Life Sciences, Imperial College London, Silwood Park Campus, Buckhurst Road, Ascot, SL5 7PY, UK

6 Forestry Research Institute of Ghana, Council for Scientific and Industrial Research, Kumasi, Ghana

7 Department of Natural Resources Management, CSIR College of Science and Technology, Kumasi, Ghana

8 AMAP (Botanique et Modelisation de l'Architecture des Plantes et des Végétations), CIRAD, CNRS, INRA, IRD,Université de Montpellier, Montpellier, France

Supplementary Method – Manual for reproducing results

The purpose of the supplementary material is to guarantee the reproducibility of all parameters used in our modelling work. As a result, it prioritizes technical details over readability, serving as a technical manual of the "Methods" chapter in the main text.

To reproduce Figure S3

The ratio of leaf internal CO_2 to eternal CO_2 (c_i/c_a) was estimated from leaf $\delta^{13}C$ measurements. We initially estimated the difference between the leaf stable isotope ratio and the atmospheric stable isotope ratio at that place and time ($\Delta^{13}C$) from $\delta^{13}C$, using the method described by a previous study [1]. Subsequently, we calculated c_i/c_a from $\Delta^{13}C$ using equation 11 in a previous study [2]. The community-weighted mean and standard error were then calculated based on 238 measurements. This isotope based c_i/c_a was compared to Pmodel predicted c_i/c_a explained in the following Pmodel experiment section (Figure S3).

The P-model requires several climate variables as input, and this can be a source of uncertainty in modelled GPP. To address this, we compared the temperature and relative humidity from local weather stations to products commonly used by vegetation models. We selected relative humidity and temperature provided by ERA-interim and shortwave radiation retrieved from ERA5-Land. However, the data-model differences we found for temperature, relative humidity and c_i/c_a (calculated from temperature and vapour pressure) were very small and not likely to contribute significantly to the GPP data-model discrepancy (Figure S3). Moreover, data-model comparisons of many climate variables have been done in previous literature [3]. Therefore, we used ERA-interim temperature, vapour pressure and calculated c_i/c_a for all GPP experiments in this study to maintain consistency and ensure a fair comparison with other models (Figure 2), while the choice of PPFD data set was specific to the experiment (Figure 3).

For satellite based fAPAR, we extracted MODIS fAPAR (MOD15A2H) of the 14 plots from 2001 to 2020 and averaged them into mean fAPAR per site. To remove cloud contaminated fAPAR, we selected only CLOUDSTATE=00 (clouds not present) and =10 (mixed clouds present). Since MODIS GPP and fAPAR share the same CLOUDSTATE, this ensures that the

fAPAR used in Pmodel_P is identical to that used in MODIS GPP (Figure 3). The issue of cloud contamination is visualized in Figure 1 and Figure 4.

To reproduce Figure 4 and Figure S2

This chapter explain how to calculate GPP for each Pmodel experiment as shown below.

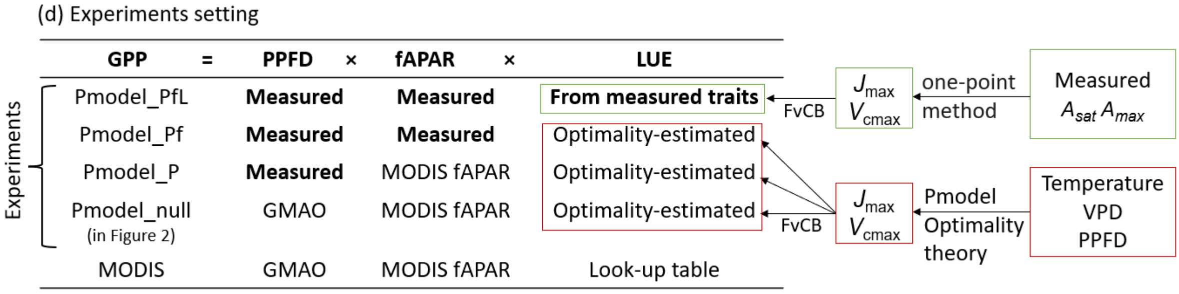

Experiment Pmodel_PfL

We used measured mean fAPAR, light-saturated photosynthetic rate (A_{sat}), light- and CO_2-saturated photosynthetic rate (A_{max}), χ, and climate variables for each site as inputs to the P-model in order to calculate 'Pmodel_PfL GPP' for each site. V_{cmax}, J_{max} and LUE can also be calculated from the above measurements. We chose field-measured A_{sat} and A_{max} instead of field-measured V_{cmax} and J_{max} because the former is simpler to measure. We have multi-seasonal samplings and lots of replicates for A_{sat} and A_{max} (which do have strong seasonal variation) [4].

fAPAR was estimated using hemispherical photography. hemispherical images were taken with a Nikon 5100 camera and Nikon Fisheye Converter FC-E8 0.21x JAPAN near the center of each of the 25 subplots in each plot in each site, at a standard height of 1 m, and during overcast conditions. 22,000 photos were collected in total, every month during 2016-2017(ANK), 2012-2017 (BOB&KOG). Photos were processed using machine learning-based software 'ilastik' [5] for pixel classification and CANEYE [6]for leaf area index calculations. The exposure procedure followed [7] and GEM manual [8] (http://gem.tropicalforests.ox.ac.uk). The following parameters were supplied to CANEYE to calculate both leaf area index (LAI) and fAPAR

(1) P1 = angle of view of the fish eye divided by the amount of pixels from centroid of the fish eye circle to where horizon is on the image.
(2) angle of view = 90 degree, in which case, the edge of the photo is the horizon and the centroid of the image is zenith.
(3) COI = 80, consideration of field is 80 degrees, we don't want the edge of the photo because it is not clear and sometime obscure by tall grasses or saplings.
(4) Sub sample factor =1
(5) Fcover = 20 degree, this is to calculate the percentage of black pixels within central 20-degree ring. We used this to understand the relative openness of canopy for the given image. It is not relevant to LAI
(6) PAIsat = 10, When a pixel is completely black, mathematically, the leaf area index (LAI) is infinite. As we provide CANEYE 25 subplot images for each estimation of LAI, this means all 25 subplot images show black at a given pixel. To address this 'infinite'issue, we used a value of 10 for LAI in such cases. This value is based on the guess that, the densest point in a tropical forest should have an LAI of 10.
(7) Latitude 0 and Day of year a random number (not relevant for tropical site LAI)

CANEYE reported a diffuse fAPAR and a direct fAPAR. We calculated overall fAPAR as 0.8*Diffuse_fapar +0.2*Direct_fapar [9]. We used 0.8 and 0.2 because We assumed that 80% of days in the study region is cloudy.

To measure A_{sat} and A_{max}, we used an open-flow gas exchange system (LI-6400XT, Li-Cor Inc., Lincoln, NE, USA). To ensure a proper representation of the forest stand, we sampled tree species that constituted approximately 80% of the plot basal area. For each species, we selected three mature and canopy emergent trees, and cut one fully sunlit and one shaded branch per tree using a single rope climbing technique. We immediately placed and recut the cut branch under water, and measured the maximum rate of net CO_2 assimilation at 400 ppm CO_2 (A_{sat}) and 2000 ppm CO_2 (A_{max}) on three leaves per branch. The PPFD was set to 2000 μmol m^{-2} s^{-1} and block temperature was kept constant at 30 ºC. Each measurement was derived into V_{cmax} and J_{max} at growth temperature [2] using R package 'plantecophys' [10,11]. Measurements were made every three months from 2014 to 2016 to cover both wet and dry seasons. Although we measured both shade and sun leaves, we used sun leaves only in this study (consistent with common practice in field studies of photosynthetic traits). We used above canopy PPFD as model input and we note that only sun leaves acclimate to this level of PPFD; shade leaves acclimate to darker environments and have consistently lower A_{sat} than sun leaves [12]. Measurements from 1394 leaves were used to calculate community-weighted means based on the basal area of each species. The same weights were applied in calculating standard errors [13].

LUE was calculated from A_{sat} and A_{max} following the method below.

We first calculate ci, using equation (1) and (2).

$$\frac{c_i}{c_a} = \frac{\Gamma^*}{c_a} + \left(1 - \frac{\Gamma^*}{c_a}\right)\frac{\xi}{\xi + \sqrt{D}} \quad (1)$$

$$\xi = \sqrt{\frac{\beta(K + \Gamma^*)}{1.6\eta^*}} \quad (2)$$

We have shown that field measured ci/ca is in good consistency with optimality estimated ci/ca (Figure S2).

We then **convert $\mathbf{A_{sat}}$ to $\mathbf{V_{cmax}}$** using 'one-point' method, equation 3 in [11].

$$V_{c_{\max}} = A_{\text{sat}}\left(\frac{C_i + K_m}{C_i - \Gamma^*} - 0.015\right) \quad (3)$$

We used the R package *Plantecophys* [10] to do so using the following codes.

```
library(plantecophys)
ghana_data2 <- fitaci(data2[i, ],
                      varnames = list(ALEAF = "Asat", Tleaf = "Mean.Tleafasat",
                              Ci = "Mean.Ciasat", PPFD = "PARi", Rd = "DResp"),
                      fitTPU = FALSE, Tcorrect = F, Patm = 100,  PPFD = 2000,
                      fitmethod = "onepoint")
# Dresp is dark respiration reported by LICOR, PARi is light environment of
# the chamber at 2000 μmol m-2 s-1, Mean.Ciasat is leaf-internal CO2 found
# by LICOR, while leaf external CO2 was fixed to 400 ppm, Mean.Tleafasat is the
# leaf temperature reported by LICOR, chamber temperature was kept at 30 degree
# so Mean.Tleafasat is around 30 degree for most measurements.
```

Supplementary Box 1. Calculation of $\mathbf{V_{cmax}}$

We then **convert $\boldsymbol{A}_{\mathbf{max}}$ to $\mathbf{J_{max}}$**, using 'Jmax limitation equation'.

However, there are two 'J_{max} limitation methods' that leads to two equations linking Amax and J_{max}. There is no consensus on which equation should be preferred. Historically, without considering J_{max} limitation, we simply have A_{max} = J_{max} / 4. A previous study [14] explained and compared the two equations. More information on J_{max} limitation see [15–17].

In our paper, we used the following equation (4), which was equation 13 in [14]:

$$A_J = \varphi_0 I_{\text{abs}} \text{m} \cdot \frac{1}{\sqrt{1 + \left(\frac{4\varphi_0 I_{\text{abs}}}{J_{\max}}\right)^2}} \quad (4)$$

with $m = \frac{c_i - \Gamma^*}{c_i + 2\Gamma^*}$.

There is also the other $\mathbf{J_{max}}$ equation (5), which was equation F20 in [14], not used in our study:

$$A_J = \left(\frac{m}{4}\right)\frac{\varphi_0 I_{abs} + J_{max} \pm \sqrt{(\varphi_0 I_{abs} + J_{max})^2 - 4\theta\varphi_0 I_{abs} J_{max}}}{2\theta} \quad (5)$$

Where A_J is equivalent to A_{max} under 2000 µmol m^{-2} s^{-1} light and 2000ppm CO_2. R package '*rpmodel'* do have both equations written in, but for an unknown reason, results given by equation (5) (called 'Smith19' in the package) have unreasonably low J_{max}. It is more likely to be a coding issue because the Python version of 'rpmodel' reports reasonable results for both methods. The Python version is called 'pyrealm' (https://pyrealm.readthedocs.io/en/latest/index.html) (https://pypi.org/project/pyrealm/). Pmodel version is out of the scope of this paper, so results are not presented here. We thus used the R package 'rpmodel' with equation (4) (named 'Wang17' in the package).

However, package '*Plantecophys*' used equation (5). To be consistent with *rpmodel*, we modified the source codes of *Plantecophys* in function *photosyn.R*, by **replacing Jfun (Box 2) with Jfun4 (Box 3).**

```
Jfun <- function(PPFD, alpha, Jmax, theta){
  (alpha*PPFD + Jmax -
     sqrt((alpha*PPFD + Jmax)^2 - 4*alpha*theta*PPFD*Jmax))/(2*theta)
}

inverseJfun <- function(PPFD, alpha, J, theta){
  J*(J*theta - alpha*PPFD)/(J - alpha*PPFD)
}
```

Supplementary Box 2 Jmax limitation Equation (5) as written in R package '*Plantecophys*'.

```
Jfun4 <- function(PPFD, alpha, Jmax, theta){
  alpha*PPFD/sqrt(1+(alpha*PPFD/Jmax)^2)
}

inverseJfun <- function(PPFD, alpha, J, theta){
  sqrt(1/(1/J^2 - 1/ (alpha*PPFD)^2 ))
}
```

Supplementary Box 3 Jmax limitation Equation (4), modified from R package '*Plantecophys*'

Now, J_{max} could be obtained by codes in Box 4

```
   ghana_data2 <- fitaci(data2[i, ],
                         varnames = list(ALEAF = "Amax", Tleaf = "Mean.Tleafamax",
                            Ci = "Mean.Ciamax", PPFD = "PARi", Rd = "DResp"),
                         fitTPU = FALSE, Tcorrect = F, Patm = 100, PPFD = 2000,
                         fitmethod = "onepoint")
# Dresp is dark respiration reported by LICOR, PARi is light environment of
# the chamber at 2000 µmol m-2 s-1, Mean.Ciasat is leaf-internal CO2 found
```

```
# by LICOR, while leaf external CO2 was fixed to 400 ppm, Mean.Tleafasat is the
# leaf temperature reported by LICOR, chamber temperature was kept at 30 degree
# so Mean.Tleafasat is around 30 degree for most measurements.
```

Supplementary Box4 Calculate Jmax from Amax using equation (4).

Note that temperature correction was turned off in the above. The outputs Vc_{max} and J_{max} are under measurement chamber temperature (around 30 degree). We used function ‘ftemp_inst_vcmax’ provided by *rpmodel* to standardise Vc_{max} and J_{max} from measurement temperature to mean annual air temperature of the site[14]. We tagged the above as Jmax_onepoint_tair and Vcmax_onepoint_tair (Figure S2).

GPP, LUE, A_J and A_C could then be calculated using Jmax_onepoint_tair and Vcmax_onepoint_tair following the procedure below.

First, A_C needs to be calculated using equation (3), under plants growing light and CO_2.

A_J needs to be calculated using equation (4) under plants growing light and CO_2.

Next, we should check whether $A_C = A_J$, following coordination hypothesis [18] (Figure S2). Upon checking coordination, we calculate the per unit leaf surface area assimilation rate following A_J only because (1) we are comparing to other light use efficiency model (MODIS) (2) Pmodel is essentially a light use efficiency model and was presented using A_J equation [14,16]

The last step is to calculate trait-based GPP and LUE from A_J (i.e. experiment Pmodel_PfL), using:

$LUE = M_C \cdot A_J / I_{abs}/\varphi(0)$ (6)

$I_{abs} = fAPAR \cdot PPFD$ (7)

$GPP = fAPAR \cdot PPFD \cdot LUE$ (8)

Equation (8) is the equation displayed in Figure 3. Relevant codes are in Box 5.

Parameters involved above are available in ‘*rpmodel*’ documentary and explained in [14]. The above calculation (i.e. experiment Pmodel_PfL) featured Jmax_onepoint_tair and Vcmax_onepoint_tair, instead of Pmodel predicted and optimality theory based Vc_{max} and J_{max}. For the later, GPP could be simply obtained by function pmodel() in package ‘*rpmodel*’ (Box 6).

```
jmax = Jmax_onepoint_tair / (10^6 /24/3600),
vcmax = Vcmax_onepoint_tair / (10^6 /24/3600), # mol C m-2 day-1
patm = calc_patm(elv),
co2 = 414,
kphio = (0.352+0.021*tc-tc^2 * 3.4/10^4)/8,
# kphio is from Peng et al New phytologist 2020
ca = co2_to_ca( co2, patm ),
## photorespiratory compensation point - Gamma-star (Pa)
gammastar = calc_gammastar( tc, patm ),
## Michaelis-Menten coef. (Pa)
kmm = calc_kmm( tc, patm ),   ##
## viscosity correction factor = viscosity( temp, press )/viscosity( 25 degC, 1013.25 Pa)
ns      = viscosity_h2o( tc, patm ),  # Pa s
ns25    = viscosity_h2o( kTo, kPo ),  # Pa s
ns_star = ns / ns25,  # (unitless)
xi  = sqrt( (beta * ( kmm + gammastar ) ) / ( 1.6 * ns_star ) ),
chi = gammastar / ca + ( 1.0 - gammastar / ca ) * xi / ( xi + sqrt(VPDmean) ),
ci = chi * ca,
gamma = gammastar / ca,
kappa = kmm / ca,
## use chi for calculating mj
mj = (chi - gamma) / (chi + 2 * gamma),
## mc
mc = (chi - gamma) / (chi + kappa),
## mj:mv
mjoc = (chi + kappa) / (chi + 2 * gamma),
a_c = vcmax * (ci - gammastar) / (ci + kmm),
fact_jmaxlim = 1/(sqrt(1+(4*kphio*ppfd_field /jmax)^2)),
a_j = kphio * ppfd_field  * (ci - gammastar)/(ci + 2 * gammastar) * fact_jmaxlim,
# unit is mol C /m2 /day
LUE = a_j * c_molmass / (ppfd_field /4.6) ,
# ppfd is in unit (mol /m2 / day), use 4.6 to convert to MJ/m2/day, now LUE is in gC/MJ
a_coordinated=(a_j+a_j)/2,
# follow A_j only after checking coordination hypothesis
gpp_calculated = a_coordinated *FAPAR_field*  c_molmass / 10^6 * 10000 * 365
```

Supplementary Box 5 Calculation of trait-based GPP and LUE for experiment Pmodel_PfL.

Experiment Pmodel_null, Pmodel_P and Pmodel_Pf

The R package ‘rpmodel’ can be used to predict values of χ, V_{cmax} and J_{max} from climate variables. These values, combined with MODIS fAPAR (MOD15), were used to derive the Pmodel_P. Combined with field-measured fAPAR, they were used to derive Pmodel_Pf. Combined with MODIS fAPAR and ERA5 PPFD, they were used to provide Pmodel_null (Figure 3).

Pmodel_null, Pmodel_P and Pmodel_Pf share the same codes as shown below, just with different input variables of fAPAR and PPFD (Figure 3).

```
library(rpmodel)
```

```
  rp_model_output<-rpmodel(
     tc=temp_result$tc,
     vpd=temp_result$VPDmean,
     co2=temp_result$co2,
     fapar=temp_result$FAPAR,
     ppfd=temp_result$ppfd ,
     patm = NA,
     elv=temp_result$elv,
     beta = 146,
     soilm = 1,
     meanalpha = 1,
     apar_soilm = 0,
     bpar_soilm = 0.733,
     c4 = FALSE,
     method_jmaxlim = "wang17", #Other options are "smith19" or 'none' "wang17",
     do_ftemp_kphio = TRUE,
     do_soilmstress = FALSE,
     returnvar = NULL,
     verbose = FALSE
  )
rp_model_output$gpp<-rp_model_output$gpp / 10^6 * 10000 * 365
#from g C m-2 day-1 to MgC per ha yr-1
```

Supplementary Box 6 GPP calculation using Pmodel predicted and optimality theory based Vc_{max} and J_{max}

Supplementary figures

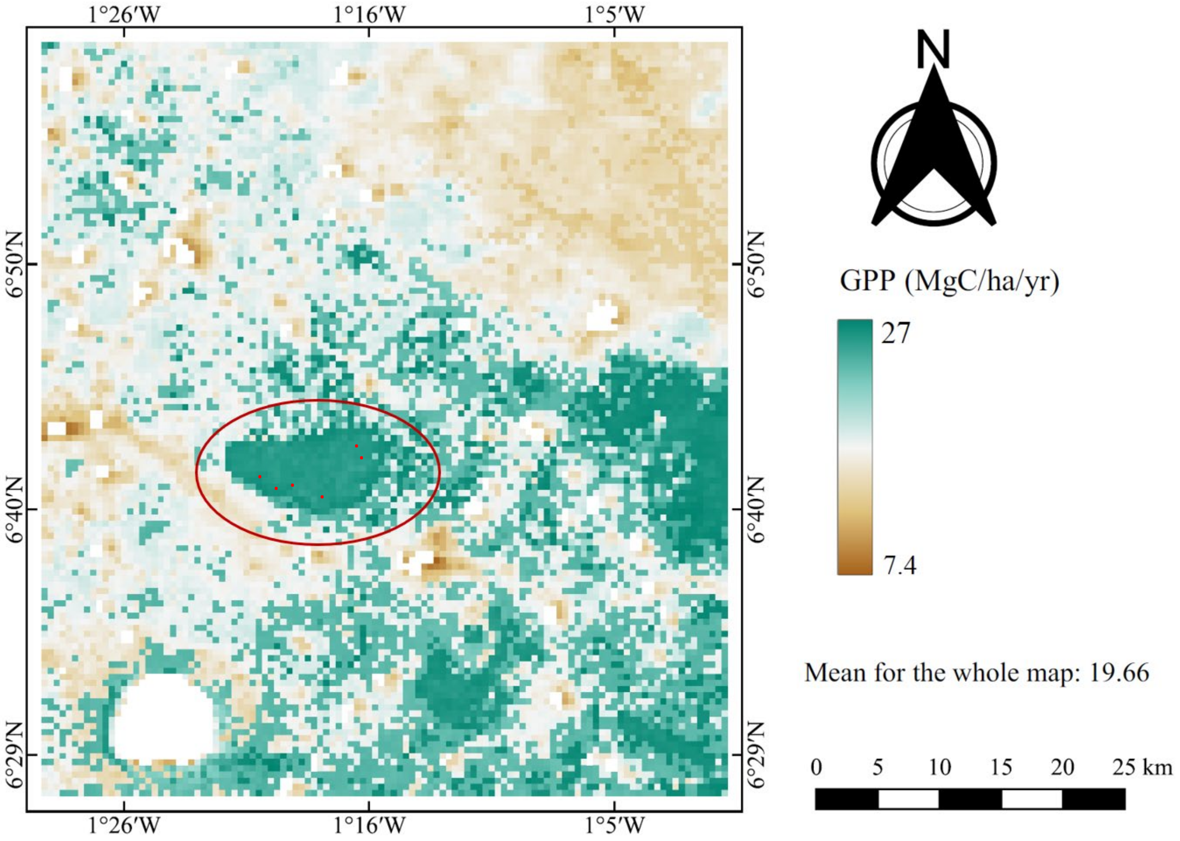

Figure S1 MODIS gross primary productivity (GPP). The red circle highlights Bobiri (BOB) forest reserve, surrounded by farmland. Six red dots denote six plots (100 x 100 m) established for GPP measurements in this forest reserve. The whole map area is roughly equal to a grid cell in TRENDY models, the typical resolution of which is half-degree (or 50 x 50 km). We visualized the GPP of the area averaged over the period 20010101 to 20201231 using product MOD17A3HGF (500m resolution, much finer than TRENDY), which has been cloud contamination-filtered and gap-filled. The figure shows that (1) West African forests are very fragmented and the study site BOB is surrounded by farmland, which makes plant functional type recognition an important issue in modelling (see hypotheses for Objective 3 in main text). (2) MODIS GPP and TRENDY models are on different resolution. Study plots fall into different grid cell of MODIS but into the same grid cell of a carbon model.

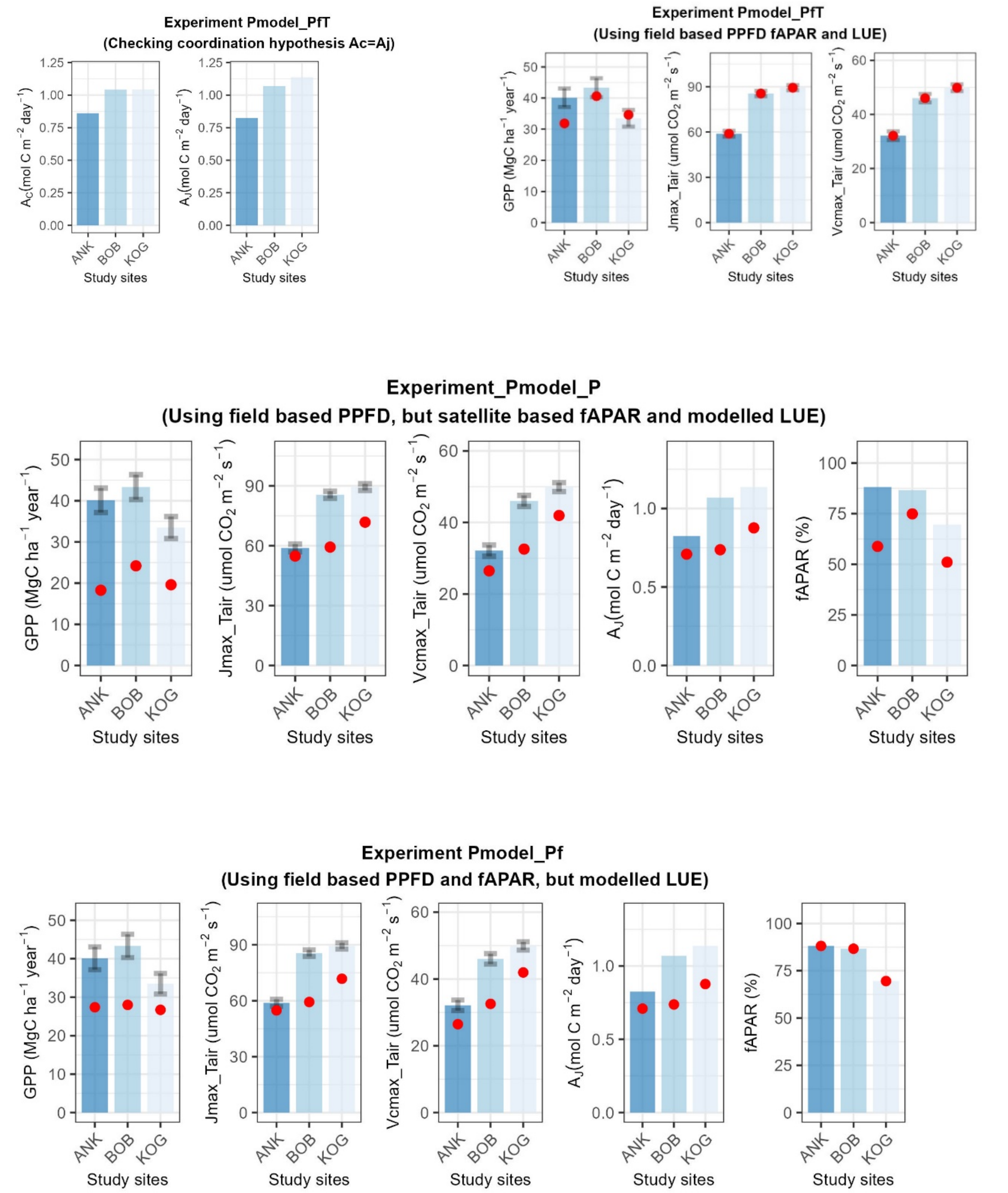

Figure S2 Comparing biometric gross primary productivity (GPP, MgC/ha/year) (Blue bar) to GPP of multiple experiments (red dots). Precipitation decreases from ANK to BOB, KOG. The figures also show data (blue bars) model (red dots) comparison for Rubisco carboxylation capacity (Vcmax_Tair, umol CO_2 m^{-2} s^{-1}), and electron transport capacity (Jmax_Tair, umol CO_2 m^{-2} s^{-1}) under mean annual air temperature, photosynthesis on the enzyme-limited (A_C, mol C m^{-2} day^{-1}) and electron transport-limited (A_J, mol C m^{-2} day^{-1}) rates under mean annual air temperature. Note that for experiments Pmodel_P and Pmodel_Pf, Pmodel predicts Vcmax and Jmax with the assumption that $A_C = A_J$ (coordination hypothesis). Therefore, only A_J was

displayed. For experiment Pmodel_PfT however, A_C and A_J are independently calculated and thus A_C is not identical to A_J. All variables were calculated under mean annual air temperature not standardized to 25 °C.

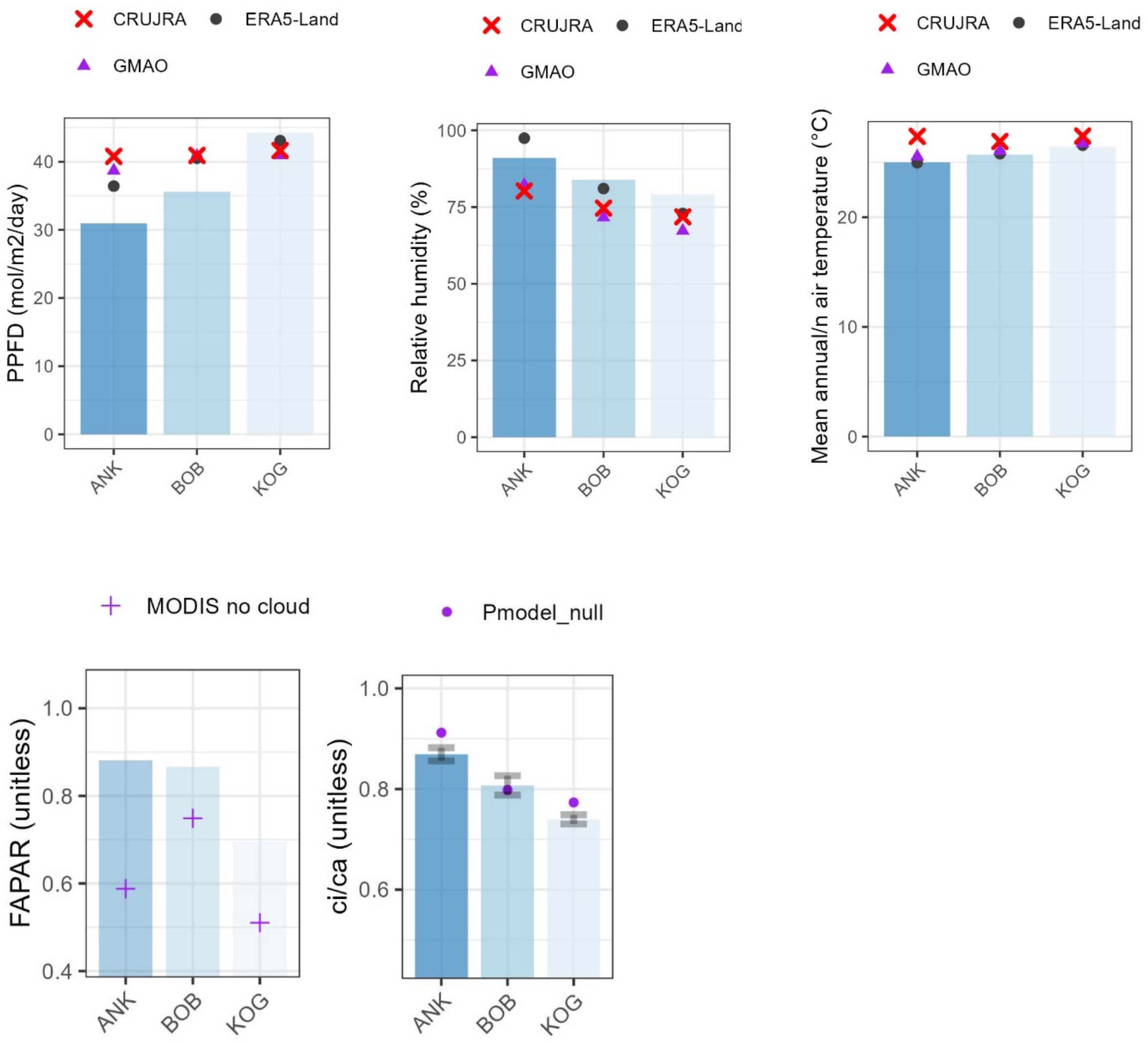

Figure S3 Field measurements (blue bars) and models (dots and marks) comparison for relative humidity (%), mean annual temperature (°C), leaf internal to external CO_2 concentration (c_i/c_a, unitless), the fraction of absorbed photosynthetically active radiation (fAPAR, unitless) and photosynthetic photon flux density (PPFD, mol/m^2/day)). Climate variables are from the University of East Anglia Climatic Research Unit Japanese Reanalysis (CRUJRA), ERA5-Land and MERRA-2 by Global Modeling and Assimilation Office NASA (GMAO). FAPAR is from MODIS MOD15A2H without data marked as ’01 Significant clouds were present. Ci/ca is shown as community weighted mean at each site with standard error.

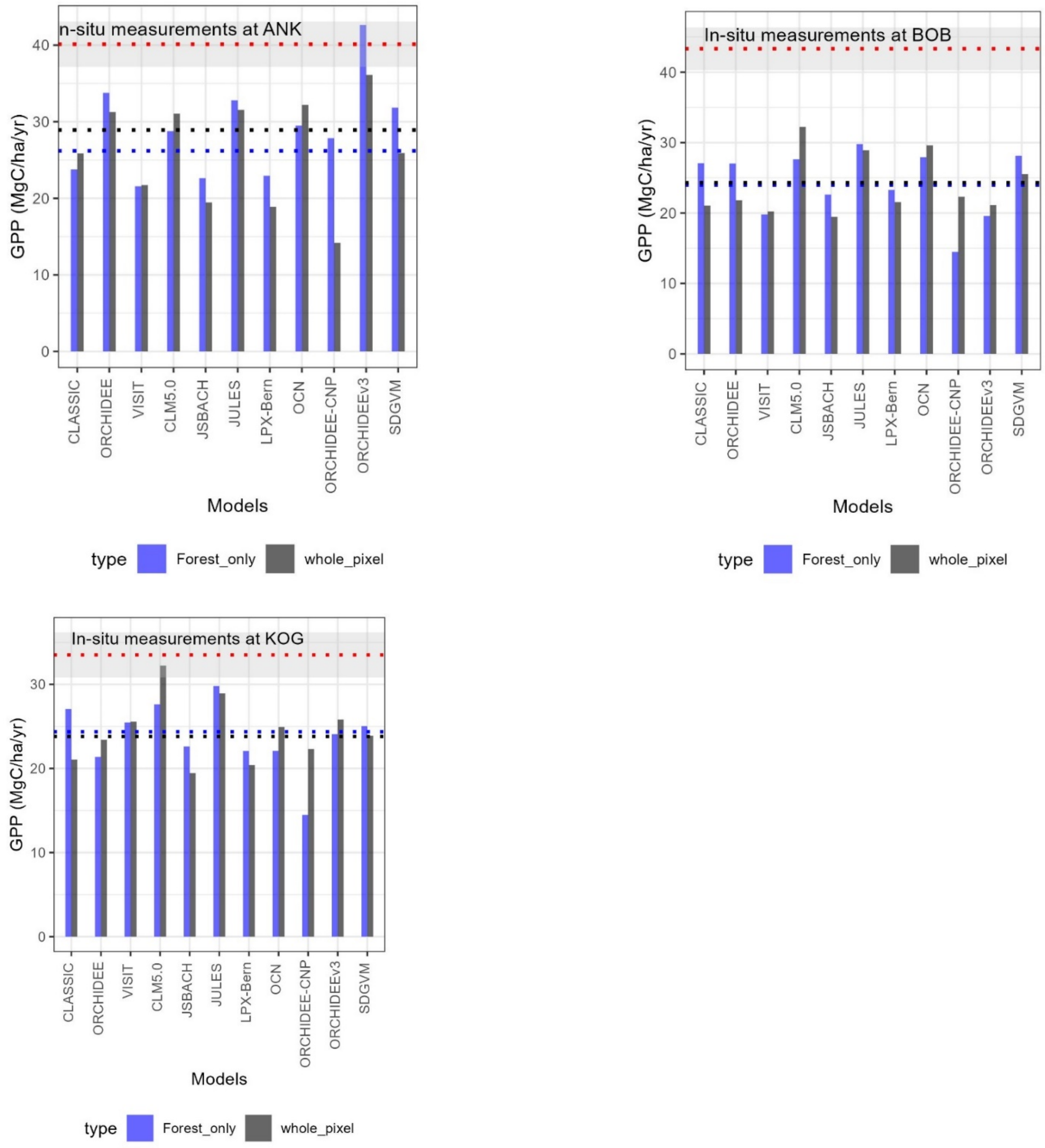

Figure S4 Comparing Forest-only gross primary productivity (GPP) (blue) to the GPP of the whole gridcell (black), which is a mixture of agricultural land, forest and grass at the three study sites, with percentage varying considerably among models. The forest-only GPP is not a proportion of whole-pixel GPP, but a potential GPP if the whole grid cell was presumed totally forested. The analysis is only possible for TRENDY models that reported GPP of each plant functional type. The red line and grey zone denote field measured GPP and its uncertainty. Figure 2 has more models than Figure S 4 because some models do not report GPP per plant functional type. The readme file of LPJ-GUESS asks not to upscale GPP (variable gpp_pft) based on land cover fraction (variable landCoverFrac), because of which LPG-GUESS was neglected.

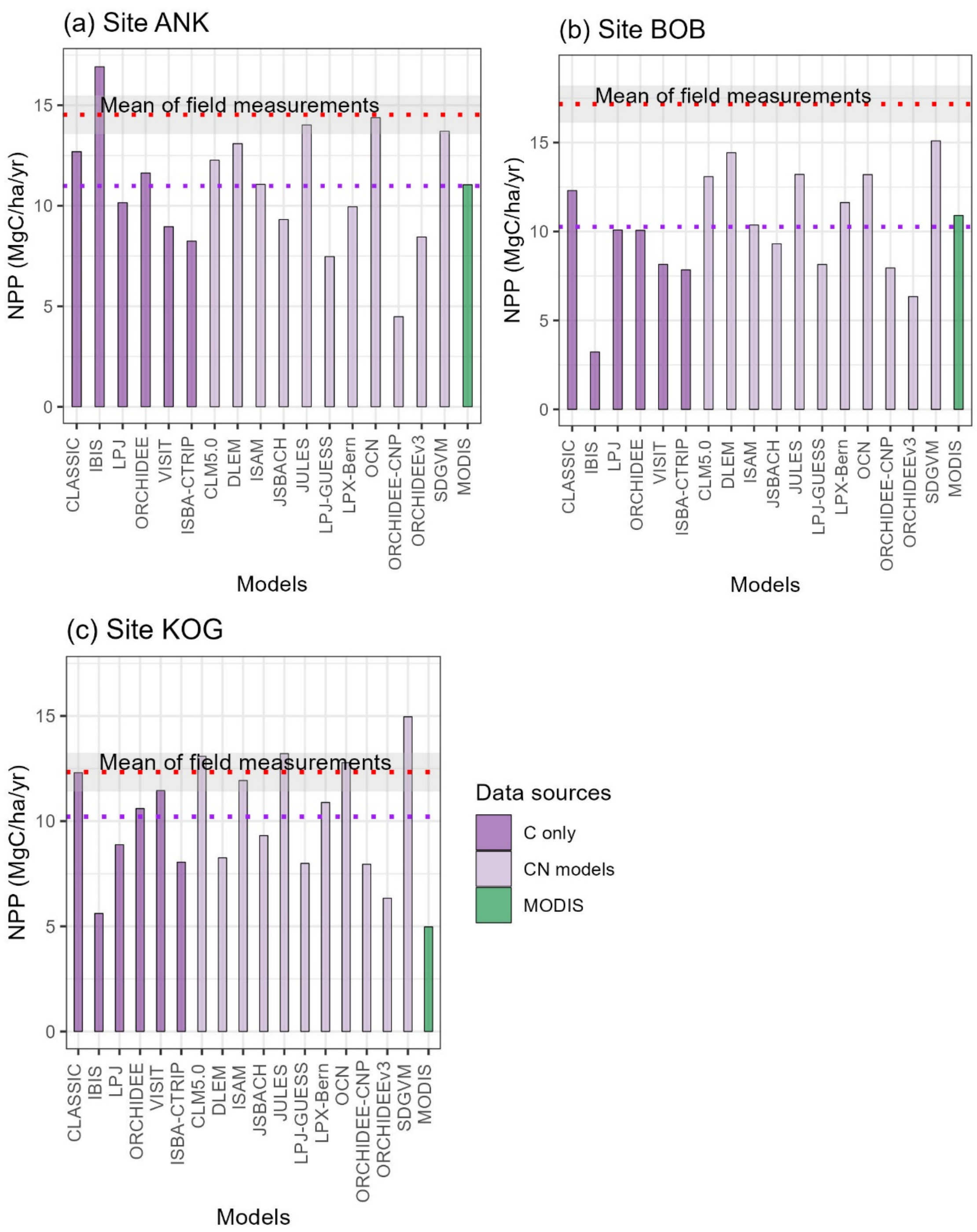

Figure S5 Intercomparison of net primary productivity (NPP, MgC/ha/year) from various independent sources. The figure contains study sites (a) Ankasa, (b) Bobiri and (c) Kogaye. The red dotted lines denote in situ biometric NPP, as a mean of multiple one-hectare plots (Table S3). The grey areas denote measurement uncertainty, not standard error. The uncertainty is calculated through error propagation. Bars denote Carbon only TRENDY models (dark purple), Carbon-Nitrogen coupled TRENDY models (light purple), and MODIS (green), as an average of mean annual GPP from 2011 to 2016. Error bar shows the maximum and minimum annual mean NPP value across the study period from 2011 to 2016. The purple dotted line denotes all TRENDY models' average

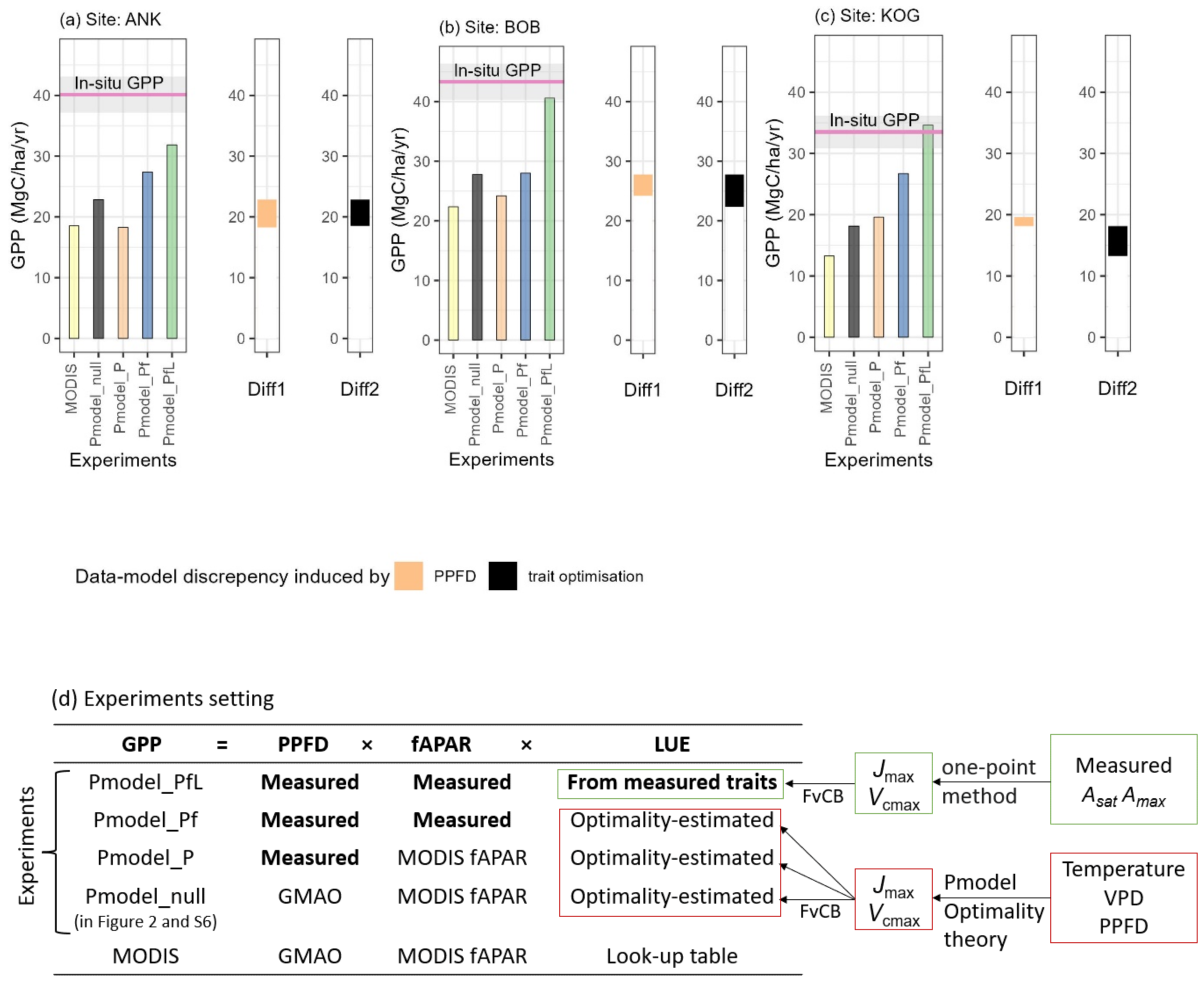

Figure S6 Partitioning of GPP data-model discrepancy, by comparing different experiments. The figure is similar to Figure 3 but incorporates Pmodel_null. Results are shown for study sites (a) Ankasa, (b) Bobiri and (c) Kogaye. The left panel displays field-based biometric GPP (pink line with grey zone showing uncertainty) and multiple GPP experiments (bars). Pmodel_PfL, Pmodel_Pf and Pmodel_P are GPP experiments which are all simulated using Pmodel but with different inputs, explained in (d). Both Pmodel and MODIS GPP were calculated from equation (1), enabling direct comparison. The right panel, a 'Diff' bar, illustrates the difference in GPP between experiments, which represents the sources of GPP data-model discrepancy. The comparison between Pmodel_null and Pmodel_P (orange, Diff1) shows that GAMO PPFD leads to overestimating GPP at ANK and BOB, but leads to underestimating GPP at KOG. The comparison between Pmodel_null and MODIS (black, Diff2) shows the difference induced by optimality theory-based LUE.

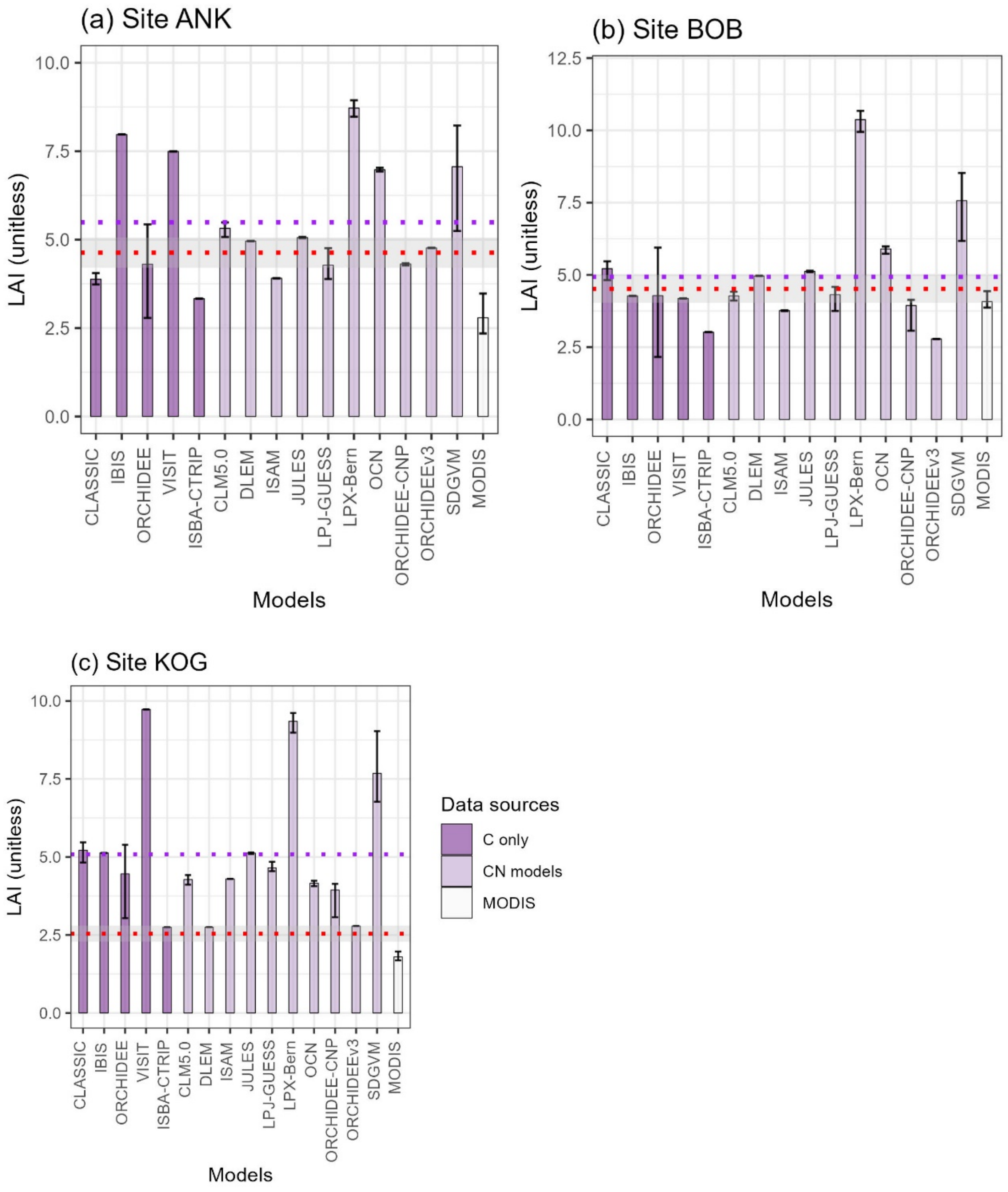

Figure S7 Intercomparison of leaf area index (LAI, unitless) from various independent sources. The figure contains study sites (a) Ankasa, (b) Bobiri and (c) Kogaye. The red dotted lines denote field LAI measurement, as a mean of multiple one-hectare plots (Table S3) [19]. The grey areas denote measurement uncertainty, not standard error. The uncertainty is calculated through error propagation. Bars denote Carbon only TRENDY models (dark purple), Carbon-Nitrogen coupled TRENDY models (light purple), and MODIS (white) as an average of mean annual LAI from 2011 to 2016. Error bar shows the maximum and minimum annual mean LAI value across the study period from 2011 to 2016. The purple dotted line denotes all TRENDY models average.

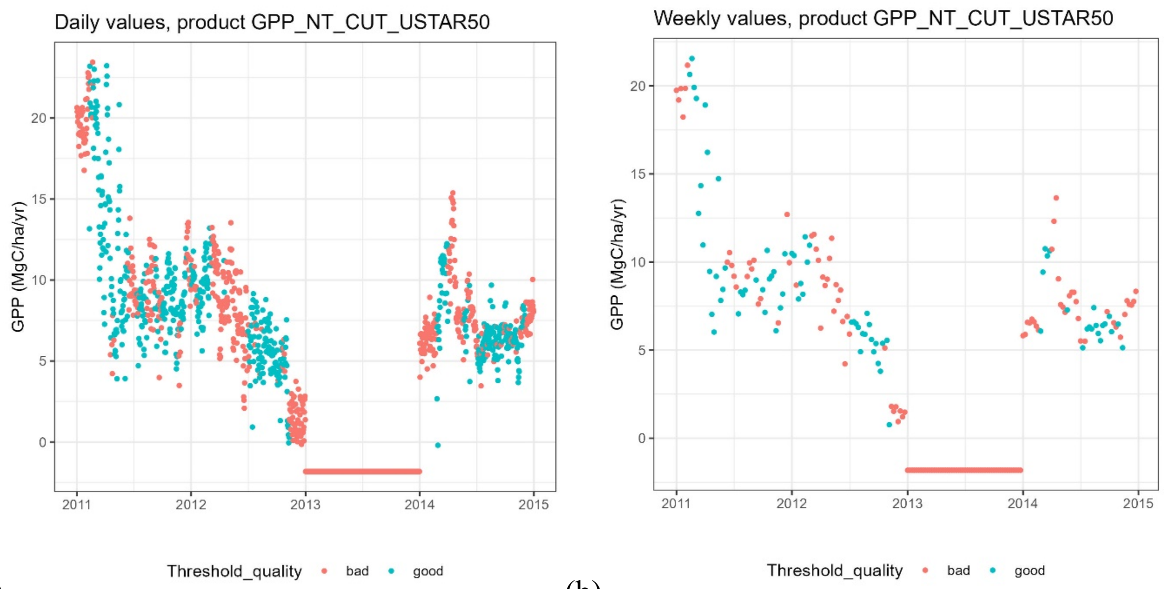

(a) (b)

Figure S8 Gross primary productivity (GPP) based on Eddy covariance data reported by flux tower GH-ANK at study site ANK, coloured as good data (blue) or bad data (gap-filled) (red). Eddy covariance data are originally half-hourly record, FLUXNET2015 database synthesized these records as daily values (a) and weekly values (b).

Table S1 Extracting variable 'landcoverfrac' from TRENDY models for each gridcell where our study sites fall into. There are three study sites: ANK BOB and KOG. We first group plant functional types into 'forests' (including evergreen and deciduous) and 'Grass, Savanna and Shrub' (including savanna, shrub C4 and C3 grass). Cropland and bare soil are omitted. Then we calculate a mean value across the models for the land cover fraction of each group. The table also shows the standard deviation, maximum and minimum land cover fraction among models. This table is a summary of Supplementary Data 1.

Model	Forests				Grass, Savanna and Shrub			
	mean	max	min	std	mean	max	min	std
ANK	0.65	0.97	0.17	0.22	0.16	0.74	0.00	0.22
BOB	0.57	0.94	0.00	0.25	0.24	0.92	0.00	0.28
KOG	0.57	0.96	0.00	0.24	0.25	0.95	0.00	0.28

Table S2 Comparing rubisco carboxylation capacity (Vcmax, umol CO_2 m2 s-1) at 25 °C for various plant functional types (PFT) among (1) DGVMs, provided by [20] (2) derived from field measured A_{sat} and A_{max} , same as Figure S2 but standardised to 25 °C, and (3) Pmodel_P experiment, same as Figure S2 but standardised to 25 °C. Column 'Vcmax25 Source' shows the photosynthesis submodule; Column 'TRENDY' shows corresponding parental DGVMs, if the module is used by one of the TRENDY DGVMs. Note that modelled values are compared to field measurements derived values of V_{cmax} to judge whether models over or underestimate (by more than 20%) V_{cmax} for West African forests. There are no field measurements for grass so a comparison is not made. Note that numerous models still lack Vcmax dynamic within PFT. These Vcmax values are recalculated for each PFT[20] and thus may not correspond to the TRENDY v9 S2 GPP shown in Figure 2. Unfortunately, after personal communications, It is not possible to retrieve Vcmax directly from TRENDY v9 simulation. C4 grass is also presented because many models tag a portion of PFT of the study sites as C4 grass. Table S2 could be downloaded in excel format via supplementary data link.

TRENDY	Photosynthesis module	Plant functional type	Vcmax25	Compared to measurements
	Field measurements derived	ANK (broadleaf evergreen)	30.49	
	Field measurements derived	BOB (broadleaf evergreen+raingreen)	39.07	
	Field measurements derived	KOG (broadleaf raingreen)	39.61	
	Pmodel_P	ANK (broadleaf evergreen)	26.54	Similar
	Pmodel_P	BOB (broadleaf evergreen+raingreen)	30.35	Underestimate
	Pmodel_P	KOG (broadleaf raingreen)	36.55	Similar
	AVIM	Broad leaf evergreen tropical tree	64	Overestimate
	AVIM	Broad leaf deciduous tropical tree	56	Overestimate
	AVIM	C 4 grass	25	
JSBACH	BETHY	Tropical tree (oxisols)	28	Similar
JSBACH	BETHY	Tropical tree (non-oxisols)	36	Similar
JSBACH	BETHY	C 4 grass	20	
CLM, LPJ	Biome-BGC	Evergreen broad leaf forest	51	Overestimate
CLM, LPJ	Biome-BGC	Deciduous broad leaf forest	47	Overestimate
CLM, LPJ	Biome-BGC	C 4 grass	60	
CLM5.0	CLM	Broad leaf evergreen tree—tropical	59	Overestimate
CLM5.0	CLM	Broad leaf deciduous tree—tropical	34	Similar
CLM5.0	CLM	C 4 grass	33	
CLASSIC	CTEM	Broad leaf evergreen	35	Overestimate
CLASSIC	CTEM	Broad leaf deciduous—dry	65	Overestimate
CLASSIC	CTEM	C 4 grass	30	
	Hybrid	Grassland	20	
	Hybrid	Deciduous	31	Underestimate
	Hybrid	Evergreen	43	Overestimate
	Hybrid	Rain forest	49	Overestimate
IBIS	IBIS	Tropical broad leaf evergreen	163	Overestimate
IBIS	IBIS	Tropical broad leaf drought-deciduous	163	Overestimate

IBIS	IBIS	Warm grass	38	
JULES	JULES	Broad leaf tree	37	Similar
JULES	JULES	C 4 grass	24	
OCN	O–CN	Tropical broad leaved evergreen	24	Underestimate
OCN	O–CN	Tropical broad leaved rain green	20	Underestimate
OCN	O–CN	C 4 herbaceous	13	
ORCHIDEE	ORCHIDEE	Tropical broad leaf evergreen trees	18	Underestimate
ORCHIDEE	ORCHIDEE	Tropical broad leaf rain green trees	22	Underestimate
ORCHIDEE	ORCHIDEE	Natural C 4 grass	28	

Supplementary information - Description of the study sites

The following study sites description information is also available at [21].

The study focuses on an aridity gradient established in Ghana, West Africa (Figure 4), that features a strong variation in aridity with only a 1.4 °C change in mean annual temperature (Table S3). The gradient contains three wet (mean annual precipitation, MAP 2000 mm) evergreen forest one-hectare plots in the Ankasa national forest (ANK-01 to ANK-03) in the extreme southwest of Ghana, close to the Côte d'Ivoire border. Two hundred kilometres to the northeast, there are six semideciduous one-hectare forest plots (MAP 1500 mm) located within Bobiri Forest Reserve (BOB-01 to BOB-06), close to the second largest city in Ghana, Kumasi. An additional one hundred kilometres to the northeast, there are six one-hectare plots located within the Kogyae Strict Nature Reserve, which encompasses the dry forest (MAP 1200 mm) to savanna transition zone (KOG-01 to KOG-06). All these plots are part of the African Tropical Forest Observation Network (AfriTRON) and ForestPlots.

The sites were at least 100 km away from each other. Plots within the same site were approximately 1 km away from each other. Therefore, the three study sites were in different grid cells of a model (or satellite product), but plots belonging to the same site were in the same grid cell. This study is thus conducted on site scale.

KOGYAE (dry, including KOG-01 to KOG-06)

The Kogyae Strict Wildlife Reserve (7.28464, -1.42414) is located near the Ejisu-Juaben District of the Ashanti Region, Ghana. The reserve covers 386 km2 and was created in 1971.

From KOG01 to KOG06, it spans from semideciduous forest to open grassland (forest-savanna transition). In the past, the forest was a continuous block that covered the whole reserve's centre and took up nearly one-third of its space. However, during the 1980s and the early 1990s, logging, farming, and bushfires all contributed to a significant loss of forest cover and the consequent fragmentation of the forest into isolated areas. The main wet season lasts from April through June with a secondary wet season from August to September, which in total delivers 1200-1300 mm average annual rainfall. The Voltarian system characterises Kogyae's geology, and exposed rocks are typically reddish-brown sandstone. The soil in the savanna and transition regions is Haplic Arenosols, with thin, sandy loam topsoil, while the forest sites have Haplic Nitosols, which are more acidic than the savanna soil [22,23].

BOBIRI (middle, including BOB-01 to BOB-06)

The Bobiri Forest Reserve (Lat 6.68697, Long -1.34401) covers 54.65 km2 and was established in 1931. It is largely covered with semideciduous and old-growth forest, which has a multilayered canopy structure, including an understorey layer composed of both shade-tolerant and shade-intolerant species, large coarse woody debris in all decay stages on the forest floor, and the presence of ferns. This site has six 1 ha plots (BOB-01 to BOB-06) with different levels of logging: BOB-01 is intact forest. BOB-02 was last logged approximately 60 years ago (2-4 stems per hectare); BOB03 and BOB04 were logged in 2015 (2-3 trees/ha); BOB05 and BOB06 were logged in 2014 using a higher intensity (felling more number of trees) than BOB03 and BOB04. The 10-yr annual rainfall ranges from 1210 to 1800 mm, with a dry season lasting from December to mid-March. The soils are Oxisols, deeply weathered, and highly acidic (3.5–4.0 in pH).

ANKASA (wet, including ANK-01 to ANK-03)

The Ankasa National Forest (Lat 5.26313, Long -2.57924) was created in 1976 and has an area of 500 km2. The reserve is mostly covered by tropical evergreen rainforest. The mean annual precipitation (MAP) is approximately 2,000 mm, mainly concentrated from March to mid-July and from September to November. A dry period extends from December to February. The relative humidity is high throughout the year; daily, it ranges from 90% at night to 75% in the early afternoon. The landscape is characterised by the presence of small, gentle hills with an average elevation of 90 m.a.s.l., such that rugged and deeply divided terrains characterise the forest, with ANK03 in the swampy valley and others on the drier hills. The deeply weathered soils are highly acidic, 3.5–4.0 in pH, and on a broad basis classified as Oxisols.

Table S3 Study plots information. These are all one-hectare plots. Data source from [21]. Table S3 could be downloaded in excel format via supplementary data link.

Plot_code	RH (%)	vwc (%)	MCWD (mm)	MAT (°C)	MAP (mm)	Lat	Lon
ANK-03	91	11.63	-13	25	2050	5.27102	-2.69234
ANK-02	91	6.78	-13	25	2050	5.268485	-2.695035
ANK-01	91	5.96	-13	25	2050	5.267868	-2.693635
BOB-03	83.9	11.37	-374	25.7	1500	6.694531	-1.293695
BOB-04	83.9	9.42	-374	25.7	1500	6.69096	-1.317038
BOB-05	83.9	8.64	-374	25.7	1500	6.692606	-1.30727
BOB-06	83.9	8.19	-374	25.7	1500	6.691368	-1.307001
BOB-02	83.9	7.87	-374	25.7	1500	6.69147	-1.338429
BOB-01	83.9	6.4	-374	25.7	1500	6.704713	-1.319068
KOG-02	79.2	4.13	-412	26.4	1200	7.262316	-1.149953
KOG-03	79.2	2.9	-412	26.4	1200	7.306792	-1.156446
KOG-05	79.2	2.54	-412	26.4	1200	7.305341	-1.164546
KOG-04	79.2	2.42	-412	26.4	1200	7.302644	-1.180213
KOG-06	79.2	1.72	-412	26.4	1200	7.329423	-1.15578

Note: (1) Volumetric water content (vwc) at site ANK, BOB and KOG are measurements of topsoil (12 cm) only. This data is provided for reference only and are not suggested for any quantitative analysis. (2) RH = relative humidity; vwc= volumetric water content, indicating surface soil moisture; MCWD = maximum climatological water deficit; MAT= mean annual air temperature; MAP = mean annual air precipitation; Lat = latitude; Lon = longitude. Plots are ranked by MCWD.

Table S3 (continue)

Plot	Elev	PPFD	Trees	P	N	C	Ca	K	Mg	Sand	Clay	Silt
	(m)	(mol/m2/day)	(#/ha)	(mg/kg)	(%)	(%)	(mg/kg)	(mg/kg)	(mg/kg)	(%)	(%)	(%)
ANK-03	86	30.97	517	109.7	0.12	1.9	40	33.7	29.2	75.9	12.8	11.4
ANK-02	124	30.97	445									15.3
ANK-01	114	30.97	476	146.8	0.17	2.6	26.8	32.3	42	63.1	21.6	15.3
BOB-03	294	35.58	631									
BOB-04	272	35.58	506									
BOB-05	246	35.58	527									
BOB-06	278	35.58	545									
BOB-02	281	35.58	789	258.3	0.16	1.7	657.6	49	133.7	46.7	28.8	24.5
BOB-01	277	35.58	519	77.8	0.09	0.8	306.3	47.6	79.7	64.2	6.7	26.8
KOG-02	229	44.26	197	67.2	0.06	0.7	378.9	42.5	75.6	82.4	2.3	15.34
KOG-03	198	44.26	216									
KOG-05	221	44.26	193	81.9	0.04	0.6	237.1	28.7	81.3	76.9	4.3	18.71
KOG-04	230	44.26	234	74.6	0.05	0.7	308	35.6	78.7	79.7	3.3	17.02
KOG-06	195	44.26	202									

Note: Plots are ranked by MCWD; Empty cells are due to the lack of data. Elev, elevation; MAP, mean annual precipitation; PPFD, photosynthetic photon flux density, calculated from shortwave radiation *0.45; Trees, total number of trees larger than 10cm diameter at breast height, as this number change from year to year, the first year is picked if a plot has multiple years censuses; Soil nutrients (P, phosphorus; N, nitrogen; C, carbon; Ca, calcium; Mg, magnesium), and soil percentage of sand (Sand) and of clay (Clay)

Table S3 (continue)

Plot	Narea	Narea_se	Nmass	Nmass_se	Pmass	Pmass_se	Parea	Parea_se	LMA	LMA_se
	g/m2		g/kg		g/kg		g/m2		m2/kg	
ANK-03	1.985	0.061	19.464	0.527	1.015	0.070	0.100	0.003	103.316	3.088
ANK-02										
ANK-01	2.075	0.083	20.745	0.682	0.944	0.085	0.089	0.005	93.349	3.291
BOB-03										
BOB-04										
BOB-05										
BOB-06										
BOB-02	2.461	0.078	27.038	0.772	1.378	0.082	0.125	0.006	86.317	1.942
BOB-01	2.620	0.105	29.611	1.211	1.296	0.158	0.142	0.010	118.623	8.340
KOG-02	2.219	0.102	18.635	0.539	1.477	0.110	0.171	0.014	109.671	3.171
KOG-03										
KOG-05	1.590	0.068	14.100	0.529	1.677	0.214	0.181	0.012	113.131	4.839
KOG-04	2.334	0.079	19.564	0.657	1.179	0.115	0.160	0.008	120.451	3.873
KOG-06										

Note: Plots are ranked by MCWD; Empty cells are due to the lack of data. Narea, community weighted mean of leaf nitrogen content by leaf area; Nmass, community weighted mean of leaf nitrogen content by leaf mass; Pmass, community weighted mean of leaf phosphorus content by leaf mass; Parea, community weighted mean of leaf phosphorus content by leaf area; LMA, community weighted mean of leaf mass per area. The _se representes standard error.

Ankasa

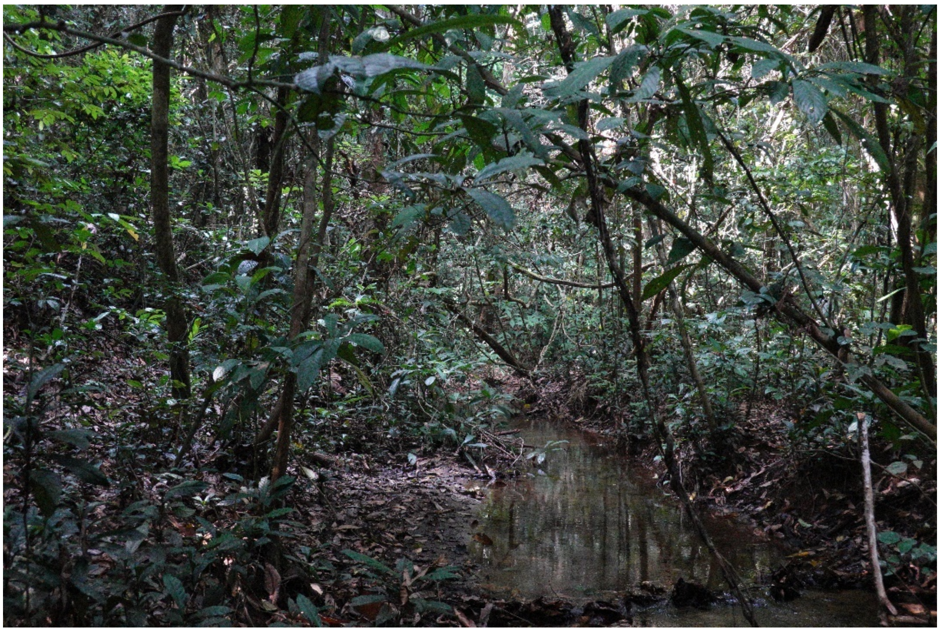

Figure S9 Field photo of study plot ANK03. There is a stream running through ANK03 which largely floods the plot in the wet season. Ankasa - ANK03. Photo Credit: Huanyuan Zhang-Zheng taken in January 2022.

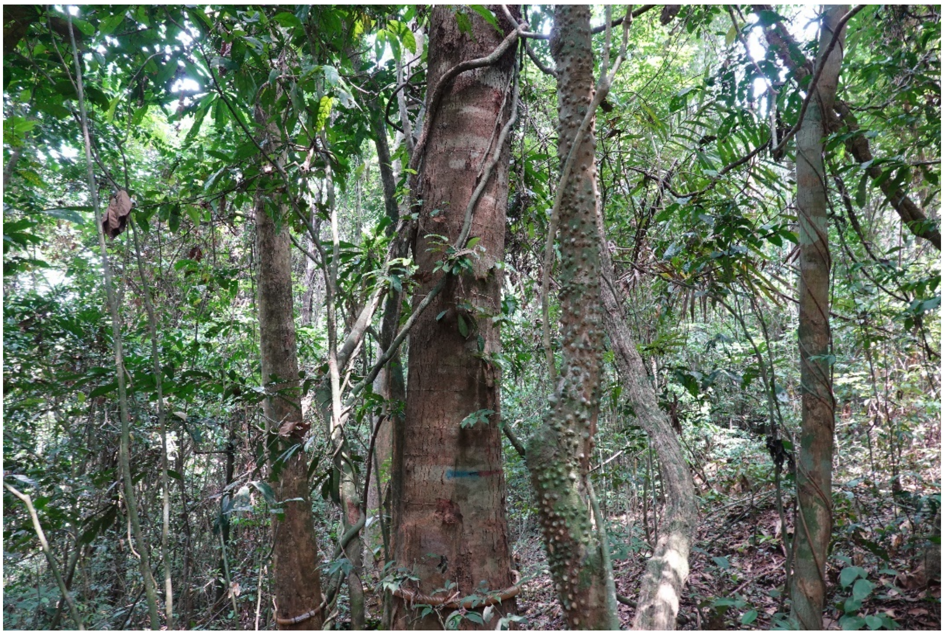

Figure S10 Field photo of study plot ANK01 and ANK02. They are located on well-drained local hilltops. The Flux Tower GH-Ank is situated next to ANK01 and ANK02. Photo Credit: Huanyuan Zhang-Zheng taken in January 2022.

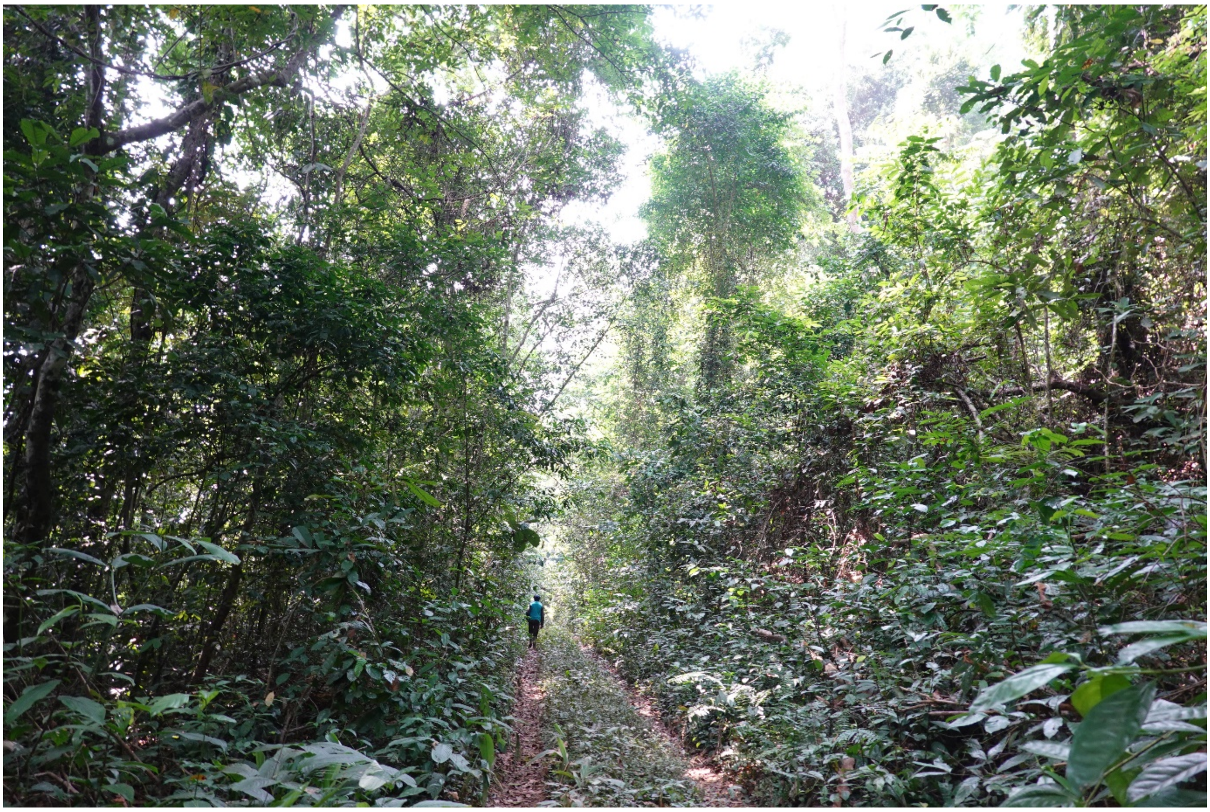

Figure S11 Field photo outside study site ANK. This is the road leading to site ANK (including the flux tower). The road is seasonally inundated so plots visitors sometimes had to walk. Photo Credit: Huanyuan Zhang-Zheng taken in January 2022.

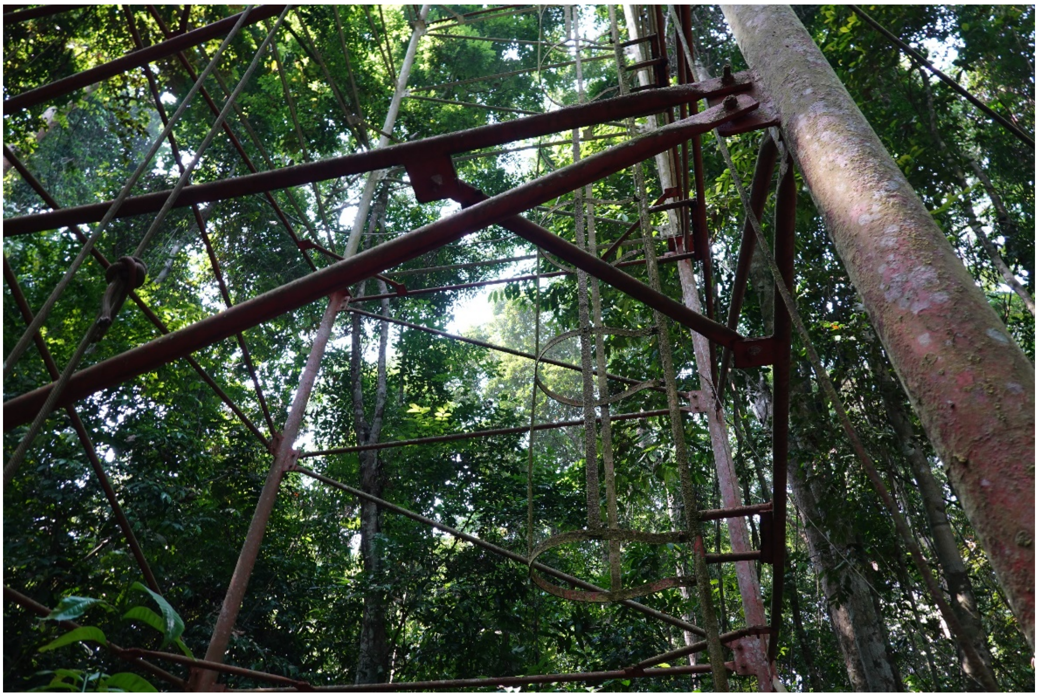

Figure S12 The flux tower (GH-Ank) operating between 2011 to 2014. The photo was taken on 2022 January 5th. Photo Credit: Huanyuan Zhang-Zheng taken in January 2022.

Bobiri - BOB01

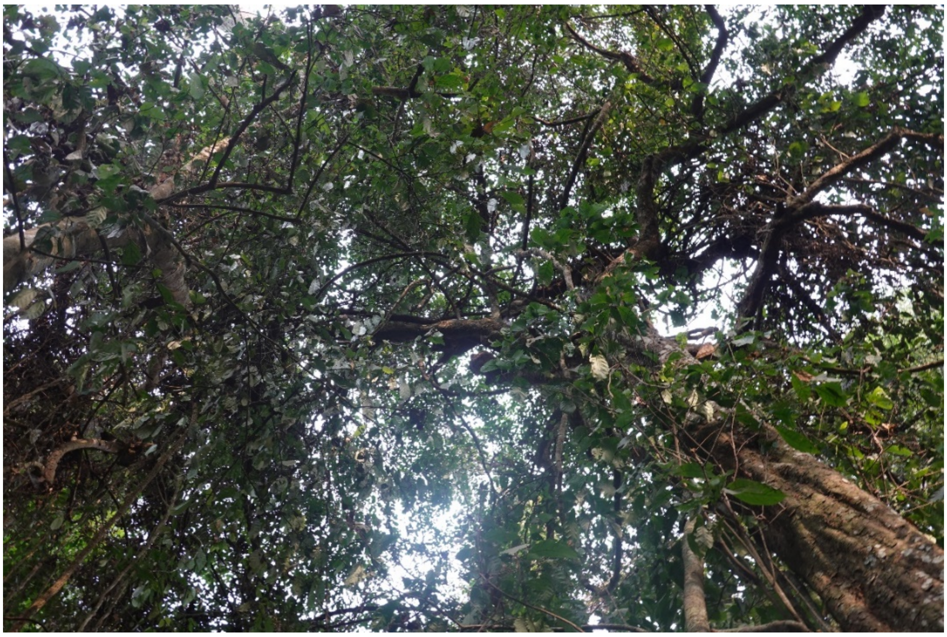

Figure S13 Study plot BOB01. Photo Credit: Huanyuan Zhang-Zheng taken in January 2022.

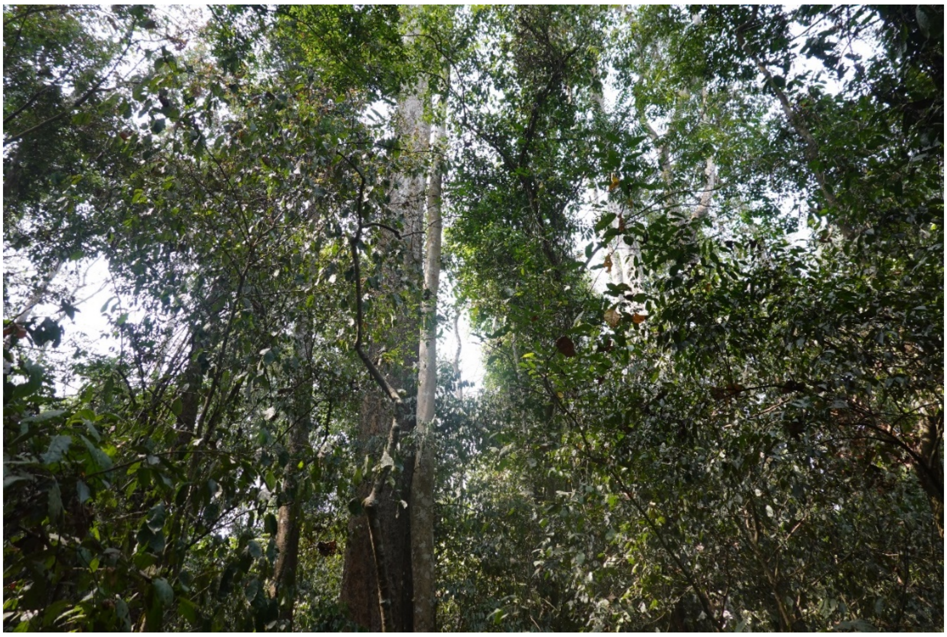

Figure S14 Study plot BOB01. Photo Credit: Huanyuan Zhang-Zheng taken in January 2022.

Bobiri - BOB02

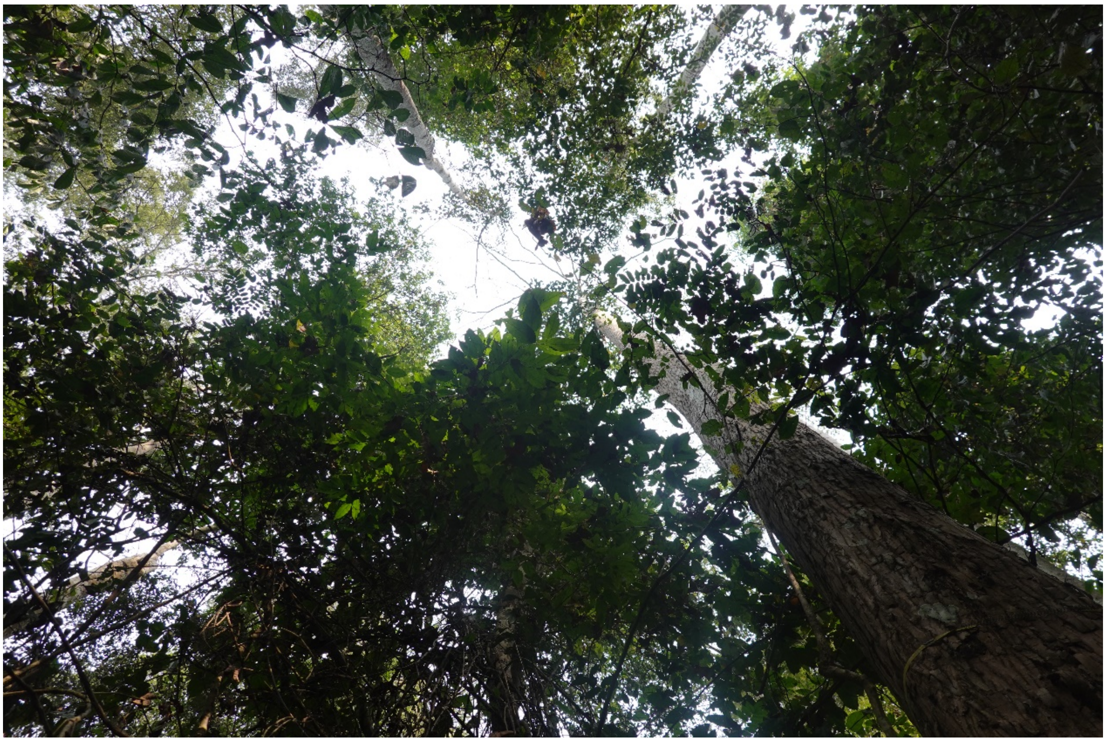

Figure S15 Study plot BOB02. Photo Credit: Huanyuan Zhang-Zheng taken in January 2022.

Kogaye - KOG01

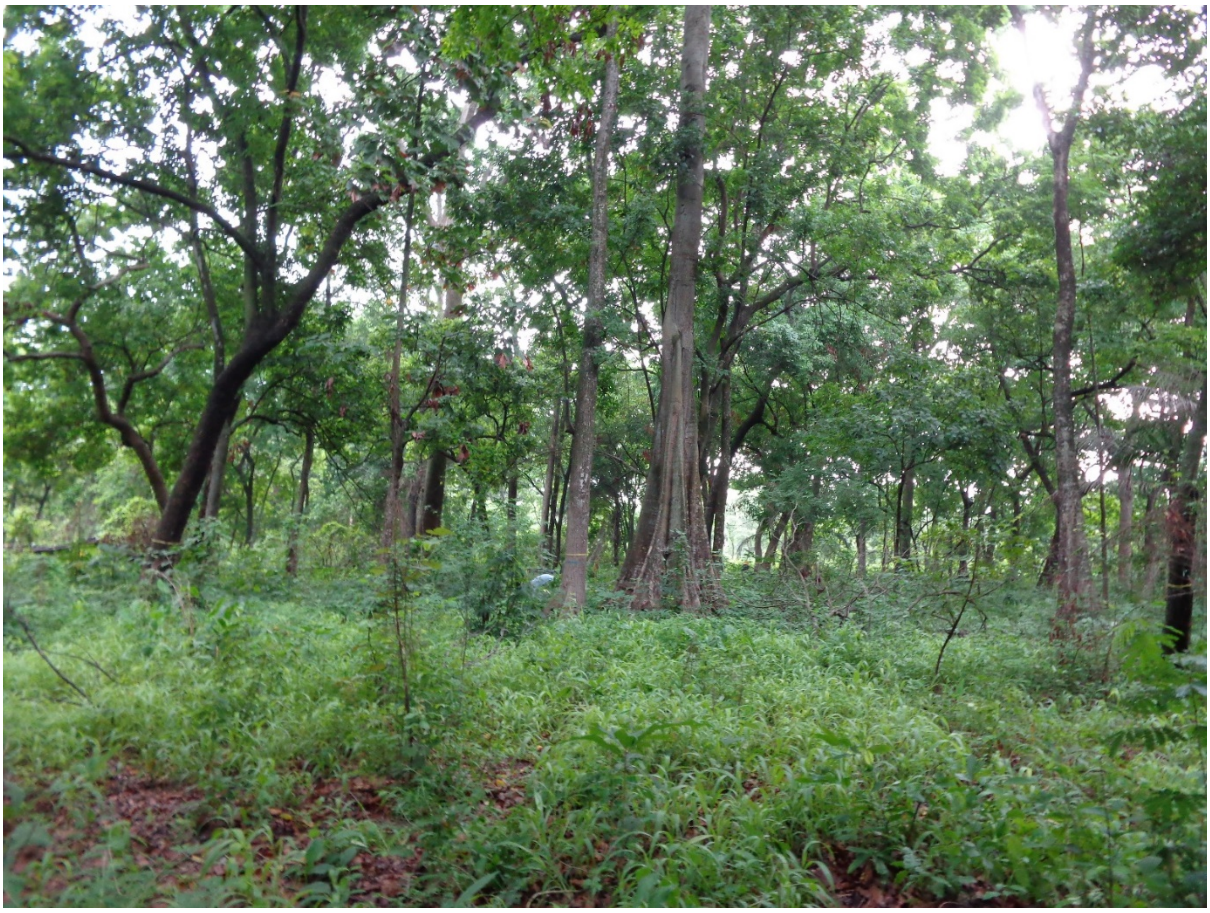

Figure S16 Study plot KOG01. Photo Credit: the photo was shared by Akwasi Duah-Gyamfi. The photo was taken on 16 July 2013.

Kogaye - KOG02

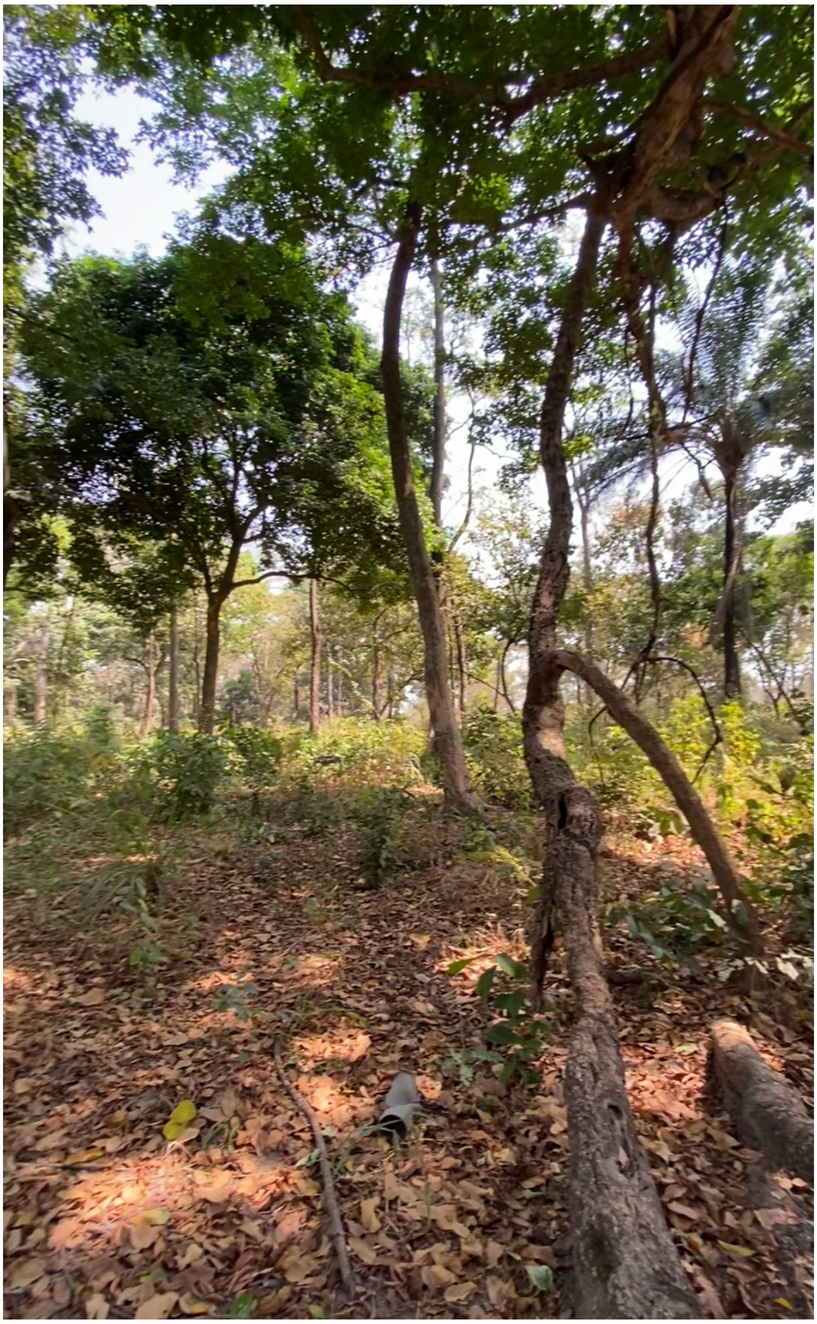

Figure S17 Study plot KOG02. Photo Credit: taken by Huanyuan Zhang-Zheng in January 2022.

Kogaye - KOG04

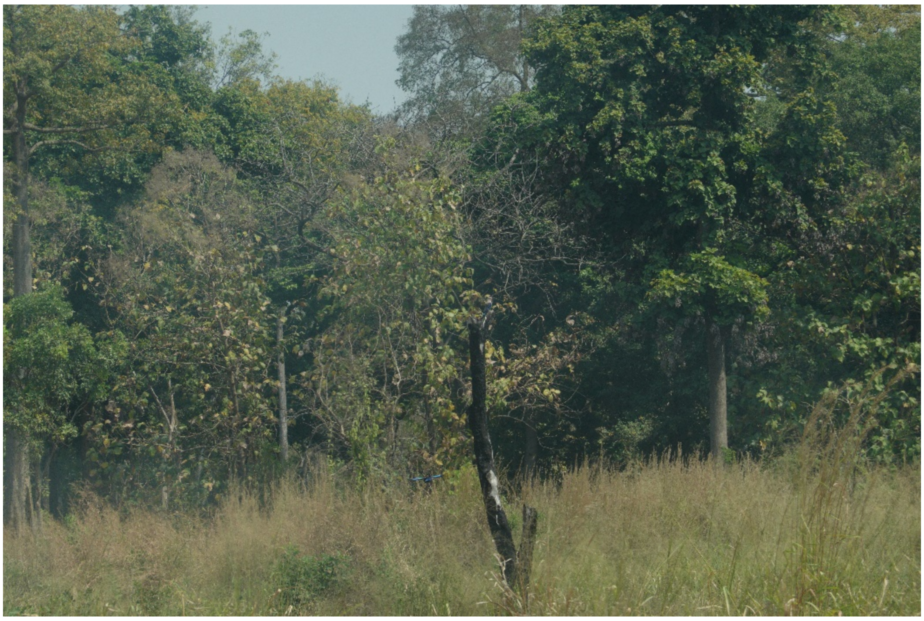

Figure S18 Outside study plot KOG04. This is at the forest-savanna transition. Photo Credit: taken by Huanyuan Zhang-Zheng in January 2022.

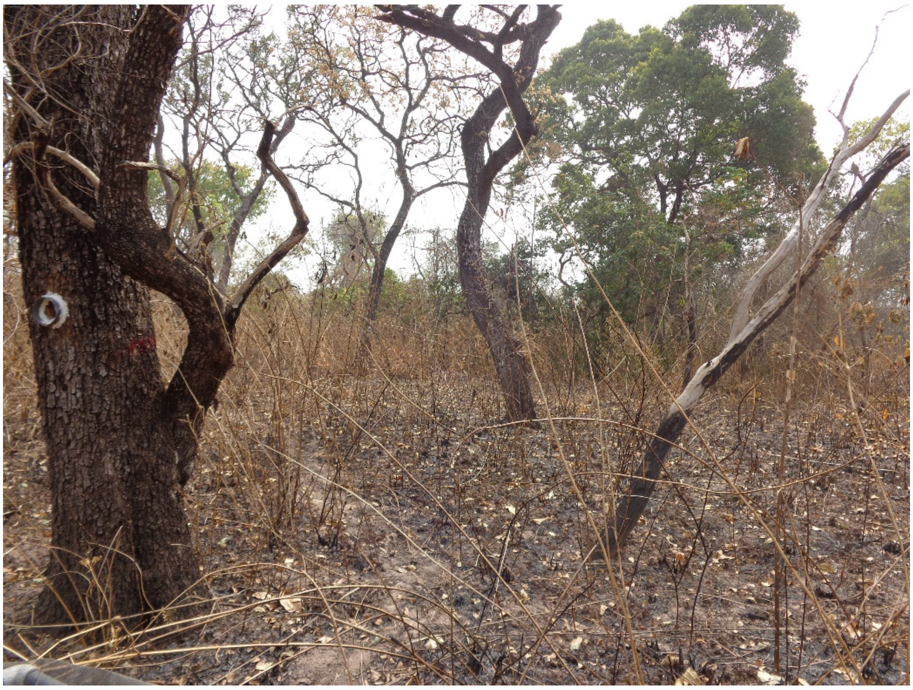

Figure S19 Study plot KOG04. This plot rarely burns (as told by locals), but it looks like this when it does burn. Photo Credit: the photo was shared by Akwasi Duah-Gyamfi. The photo was taken on 03 February 2014,

Kogaye - KOG05

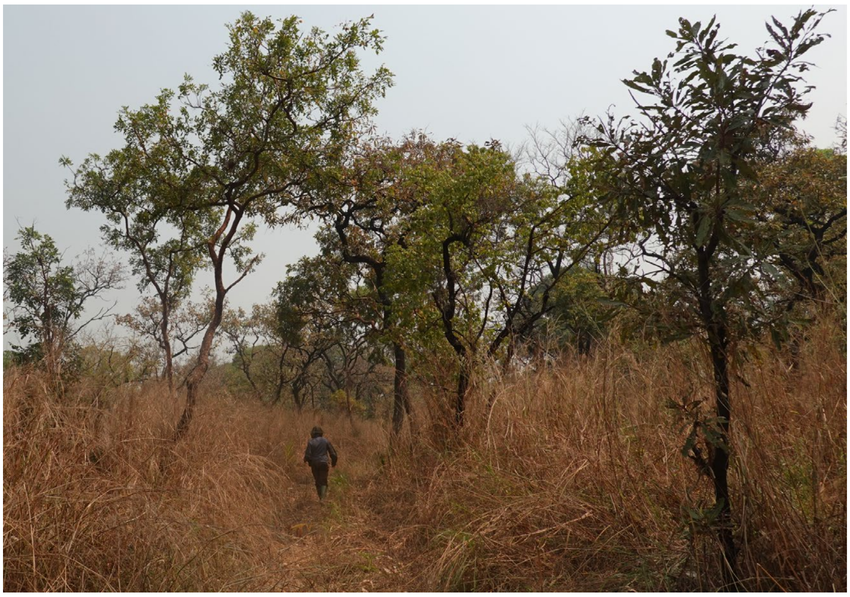

Figure S20 Outside study plot KOG05. Photo Credit: taken by Huanyuan Zhang-Zheng in January 2022.

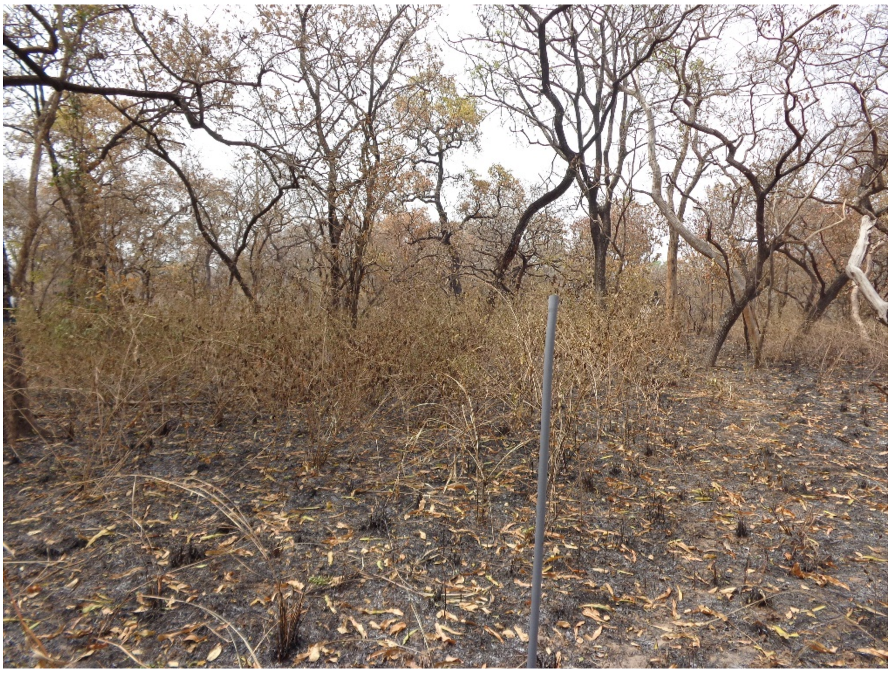

Figure S21 Study plot KOG05. This plot frequently burns. Photo Credit: the photo was shared by Akwasi Duah-Gyamfi. The photo was taken on 06 February 2014,

Supplementary References

1. Cornwell, W. K. *et al.* A global dataset of leaf delta 13C values. *Sci. Data* (2016).
2. Peng, Y., Bloomfield, K. J. & Prentice, I. C. A theory of plant function helps to explain leaf-trait and productivity responses to elevation. *New Phytol.* **226**, 1274–1284 (2020).
3. Burton, C., Rifai, S. & Malhi, Y. Inter-comparison and assessment of gridded climate products over tropical forests during the 2015/2016 El Niño. *Philos. Trans. R. Soc. B Biol. Sci.* **373**, 20170406 (2018).
4. Jin, W. *et al.* Leaf development and demography explain photosynthetic seasonality in Amazon evergreen forests. *Science* **351**, 972–976 (2016).
5. Berg, S. *et al.* Ilastik: interactive machine learning for (bio) image analysis. *Nat. Methods* **16**, 1226–1232 (2019).
6. Weiss, M. & Baret, F. CAN_EYE V6. 4.91 user manual. (2017).
7. Zhang, Y., Chen, J. M. & Miller, J. R. Determining digital hemispherical photograph exposure for leaf area index estimation. *Agric. For. Meteorol.* **133**, 166–181 (2005).
8. Malhi, Y. *et al.* The Global Ecosystems Monitoring network: Monitoring ecosystem productivity and carbon cycling across the tropics. *Biol. Conserv.* **253**, 108889 (2021).
9. Zhang, Y. *et al.* Modeling the impacts of diffuse light fraction on photosynthesis in ORCHIDEE (v5453) land surface model. *Geosci. Model Dev.* **13**, 5401–5423 (2020).
10. Duursma, R. A. Plantecophys-an R package for analysing and modelling leaf gas exchange data. *PloS One* **10**, e0143346 (2015).
11. De Kauwe, M. G. *et al.* A test of the 'one-point method'for estimating maximum carboxylation capacity from field-measured, light-saturated photosynthesis. *New Phytol.* **210**, 1130–1144 (2016).

12. Keenan, T. F. & Niinemets, Ü. Global leaf trait estimates biased due to plasticity in the shade. *Nat. Plants* **3**, 1–6 (2016).

13. Madansky, A. & Alexander, H. Weighted standard error and its impact on significance testing. *Anal. Group Inc* (2017).

14. Stocker, B. D. *et al.* P-model v1.0: An optimality-based light use efficiency model for simulating ecosystem gross primary production. *Geosci. Model Dev.* **13**, 1545–1581 (2020).

15. Bernacchi, C., Pimentel, C. & Long, S. P. In vivo temperature response functions of parameters required to model RuBP-limited photosynthesis. *Plant Cell Environ.* **26**, 1419–1430 (2003).

16. Wang, H. *et al.* Towards a universal model for carbon dioxide uptake by plants. *Nat. Plants 2017 39* **3**, 734–741 (2017).

17. Smith, N. G. *et al.* Global photosynthetic capacity is optimized to the environment. (2019) doi:10.1111/ele.13210.

18. Wang, H. *et al.* Photosynthetic responses to altitude: an explanation based on optimality principles. *New Phytol.* **213**, 976–982 (2017).

19. Zhang-Zheng, H. *et al.* Photosynthetic and water transport strategies of plants along a tropical forest aridity gradient: a test of optimality theory. 2023.01.10.523419 Preprint at https://doi.org/10.1101/2023.01.10.523419 (2023).

20. Rogers, A. The use and misuse of Vc,max in Earth System Models. *Photosynth. Res.* **119**, 15–29 (2014).

21. Zhang-Zheng, H. *et al.* Contrasting carbon cycle along tropical forest aridity gradients in W Africa and Amazonia. *bioRxiv* 2023–07 (2023).

22. Domingues, T. F. *et al.* Co-limitation of photosynthetic capacity by nitrogen and phosphorus in West Africa woodlands. *Plant Cell Environ.* **33**, 959–980 (2010).

23. Oliveras, I. *et al.* The Influence of Taxonomy and Environment on Leaf Trait Variation Along Tropical Abiotic Gradients. *Front. For. Glob. Change* **3**, 18 (2020).
