# Supplementary material for: Why models underestimate West African tropical forest primary productivity
Source: Nat Commun. 2024 Nov 6;15:9574. doi: 10.1038/s41467-024-53949-0 (PMC11541734; doi:10.1038/s41467-024-53949-0)
Supplement: Supplementary file 2 — Description of Additional Supplementary Files [file 41467_2024_53949_MOESM2_ESM.pdf]

Description of Additional Supplementary Files

File Name: Supplementary Data 1
Description: Extracting variable ‘gpppft’ and variable ‘landcoverfrac’ from TRENDY models for each gridcell where our study sites fall into. Longitude and latitude are coordinates of the study sites. ‘Name’ is the name of study site. GPP is the value contained in variable ‘gpppft’ in original unit of model outputs. Models normally have multiple plant functional type. Each has a unique ID. ‘Level’ is the id. GPP_MgC_ha_year is converted from column GPP, standardised to unit MgC_ha_year. ‘Frac’ is the percentage fraction of such plant functional type in such model at the grid cell. ‘pft’ the name of plant functional type defined by the model. ‘Unit’ explains how to link GPP_MgC_ha_year here (stored in variable gpp_pft) with total GPP of a grid cell (variable gpp). For most models, you need to multiply "frac" first, but for some model (e.g. LPJ-GUESS), the "multiplied with frac" has been done by the modeler so you just need to sum all gpp-pft records under this model. Note: frac < 10% was neglected. Model ISAM was neglected because we could not find plant functional type information in their nc files. PFT noted as 'not classified probably bare soil' implies that frac of the gridcell does not sum up to 1, the remaining was assumed bare soil as told by LPJ-GUESS user manual.
