# Supplementary material for: Why models underestimate West African tropical forest primary productivity
Source: Nat Commun. 2024 Nov 6;15:9574. doi: 10.1038/s41467-024-53949-0 (PMC11541734; doi:10.1038/s41467-024-53949-0)
Supplement: Supplementary file 4 — Source Data [file 41467_2024_53949_MOESM4_ESM.pdf]

nature portfolio

Peer Review File

Why models underestimate West African tropical forest primary productivity

Corresponding Author: Dr Huanyuan Zhang-Zheng

This file contains all reviewer reports in order by version, followed by all author rebuttals in order by version.

Attachments originally included by the reviewers as part of their assessment can be found at the end of this file.

Version 0:

Reviewer comments:

Reviewer #1

(Remarks to the Author)
Please find my review in the attached PDF.

Reviewer #2

(Remarks to the Author)
Key results
In response to several indications of high uncertainty in estimates of forest productivity, specifically for West Africa, the authors explored possible reasons for GPP data-model discrepancy. Addressing three objectives, the authors compared biometric GPP to various models and satellite-based products, interrogated the consistency of in situ high productivity to leaf photosynthetic traits and field observations usually simulated by models, and substituted model parameters with field observations to evaluate the data-model discrepancies. This was done at three sites with different forest structure and along a rainfall gradient in Ghana. Results indicated that GPP models generally underestimate the GPP when compared to the field measured data. They also identified that fAPAR and LUE from satellite products are mostly responsible for the data-model discrepancies and increasing field observations to inform models may be required to reduce uncertainty in model estimates.
The study has wide significance to the global modelling community, especially for carbon cycles, and highlights the need for increased field observations in forest areas in Africa that is highly recognised. This may even be true beyond forests and the results may extend to other ecosystems, thus pointing out that increasing model accuracy may require investigations like this to be rolled out more extensively.

Validity
The data interpretation and conclusions are valid and the authors covered the important aspects of the topic. They also point out the limitation of their results.
Significance
The results of the study validates what has been implied by many studies that used GPP models. It also narrows down some of the possible causes of model discrepancy. Although the study was conducted at a local scale it certainly has important implications for various global and regional models.

Data and methodology
The methodological approach is logical and comprehensive and the data appears to be of good quality. A description of the equipment used for field measurements may be useful.

Analytical approach
The analytical approaches are valid and sufficient to test the hypotheses.

Suggested improvements

1. In line 478 the authors mention “possible bias in field observations” – perhaps they could be more explicit about what

such bias would entail.
2. As far as I can tell from the manuscript, the authors compared MODIS fAPAR data averaged over 2001-2020 to field data averaged over 2012-2017. This does not make sense as it introduces environmental variability that is not accounted for by the field data collection period. Could the authors address this or clarify. Perhaps being more clear about the observation periods of the different data sources in the main methods section could also be useful.
3. Typo line 708: “uses” should be “used”
4. Typo line 709: “had” should be “have”
5. Typo line 726: “above –anopy” should be “above-canopy”
6. Lines 950-954: the season description is repeated twice
7. Lines 965-966: I was wondering what the logging status of BOB-03 and BOB-04 were?

Clarity and context
The manuscript is clearly written and it is easy to follow the methodology and approaches. The study also provided sufficient and relevant background and created good context that highlights the importance of the study.

References
The references are appropriate and relevant.

Reviewer #3

(Remarks to the Author)
Main comment

The study by Zhang-Zhen et al. seeks to explain why models currently underestimate West African tropical forest (gross) productivity, which is a crucial question for the global carbon cycle. Especially given the importance of tropical forests and the fact that African (rain)forests have been long neglected in terms of observations (and hence data constraints for models). This study is based on previous (and amazing!) findings of very large GPP in multiple forest sites in Ghana, and builds on a dataset that was recently published in the very same journal.
The study starts by comparing field-based observations of GPP with multiple model (types): DGVMs with Trendy v9, and remote-sensing (RS) estimates.

I was surprised to see that the RS products exactly correspond to what was used in the previous study and hence by the overlap with an existing manuscript. The authors then dive into the reasons why the models and the observations might diverge. They designed a series of experiments to explain the mismatch between observations and simulations. They show very nicely how underestimated fAPAR biases the estimates of GPP as well as the importance of locally measured field traits like Vcmax. However, there is almost no attempt to explain why DGVM might underestimate GPP. The authors looked at the distribution of traits in the models but they could not trace back the values that were actually used in the simulations (they are indeed not reported but a simple email to the different model contributors should have been sufficient to get those values). Also, Trendy is forced with crujra (if I remember well) but there was no attempt to check biases and uncertainties in those drivers compared to locally available ones nor with simulated LAI. I believe adding this piece somehow would greatly improve the quality of the study.

In short, I think the authors managed to achieve objective 1 (but some overlap with a previous study, which might be problematic in editor's eye), objective 2 (the biggest finding of this study) but only partially objective 3 which is the core (and the title) of the paper. They managed to explain the discrepancy between MODIS and observations with the underestimated fAPAR (which is per se not mindblowing as everybody knows the cloudiness in the tropics should make users cautious) but did not explain the mismatch between Trendy and observations (nor tried to do so), which was very disappointing.

Finally, I found the manuscript sometimes hard to read and the figures of rather poor quality. I think its structure could be significantly improved, the figures enhanced, and the text very much polished (some sections, especially in the supplementary, need to be completely rewritten in my opinion).

Other minor comments
I think you could make the abstract more appealing by adding some of the numbers you found: e.g. L61 “higher” but how much?, L64: underestimate (by how much?), etc. Those numbers would give the significance of the discrepancies.
Wouldn’t it be interesting to also look at NPP as all those models that should also report those values (it is unlikely that the models would capture NPP while being so wrong about GPP but you never know: they might be right for the wrong reasons, which would reinforce your findings!)
L52: works → work
L86: the seasonality is also a bit question in tropical forests. And while it is a great aspect of your field data, you don’t try to benefit from it, why? Wouldn’t it be interesting to check how wrong the models are in terms of dynamics? Would also reinforce your point about clouds and fAPAR for MODIS, I suppose.
L87-88: This sentence reads weird, I suggest rephrasing
L91-92: same as above, suggest rephrasing
L98: too much detail for the intro
L104: Or a mismatch in the drivers of photosynthesis?

L134: first reference to a figure is to Figure 3… The order of the figures should be corrected!
L168: reference to an experiment that was not yet described (PfL)
L184: this is not my reading from Zhang and Ye and certainly not the most up-to-date list of GPP products … The authors need a better reason to select those specifically..
L185: Trendy version? Why use version v9 while now v12 is available?
L208: rpmodel already mentioned earlier.
L228: Asat AND Amax, remove "see equations…" already mentioned earlier
L232: weird phrasing… Do you need to strengthen confidence in the field estimates? I suppose this was discussed at length in the previous paper but here it comes out of the blue
L240: remove "also see introduction"
L240 and following: this is repeated from the intro
L243-244: rephrase
L257: slight → small
L285: Which Trendy scenario was used? → It is given but way too late → reorganize the methods!
The structure of the methods could be better. It is currently messy and hard to follow. Trendy and MODIS for instance are referred to many times before it is explained what they are exactly.
L299: Does it make sense for Trendy? I suppose you should check the actual Trendy drivers..
L301: "see results" is useless
L312: Why talking about Trendy in the MODIS subsection? Plus it was already explained..
L321: shouldn't the fluxtower data be used for objective 1?
L334: Did you average over 20 years? Why not take the monthly values and also evaluate the seasonality/interannual variability?
~L380: Comparison with RS was already reported … What about the model ensemble mean?
L392: Do you mean the mean discrepancy across sites?
L399: 'short of biometric GPP' is a weird formulation
L414: the "peak" is rather flat (at least with the axis chosen for Figure 5), either quantify the increase in the data or reformulate
L418: It would be good to add fAPAR with cloud filtering/gap-filtering as well in Figure 5. Your datasets suggest rather constant fAPAR over time (and almost across sites), which is nice and interesting!
L444: but you mainly investigate GPP here… Couldn't they have similar NPP but very different GPP?
L447: and (not or)
L458: but ERA was not used for Trendy…
L476: probably better to avoid those qualitative statements ("a modest, though greatly reduced") and replace them by more objective values.
L506-508: If you could quantify this for your region of interest (Figure 1) or even the tropics or refer to studies that do would strengthen your message!
L522: To me, if you are not 100% confident which Vcmax values were used in the actual simulations, that discussion makes no sense..
L551-L552: why not report the fluxtower data with the other estimates of GPP?
L552-553: this belongs to method
L572: should?
L585: likely?

Figure 1: rather than a specific day → average frequency of cloud for the studied period (or the rainy season)? Switch subplots a and b (from more global to more local). Why restrict to that specific area? Wouldn't it be nice to see the full "cloud issue" over the tropics?
Figure 2: The dotted purple line is not described, I think? I suggest moving the individual DGVM/RS to supplementary and only keep C-DGVM (mean and distribution), CN DGVMs, All DGVMs, All RS products, P-model (null and _Pfl). The order of the DGVMs changes between subplots which makes the comparison difficult…
Figure 3: don't refer to figure 4 in the caption of Figure 3. Wouldn't it be interesting to test the other scenarios: like measured LUE and fAPAR with ERA-Interim for instance? Just to get the full picture?
L711-713: Does this calculation make sense?
L726: anopy → canopy
L727: not clear if the outliers are detected at the community or species level → More details: how many were removed?
Section starting at line 734 should be rewritten and reformatted
L748: remove "we will use this later"
Figure S1: rather than reporting the mean, a density distribution would be more informative
Figure S3: what do the error-bars represent? Why aren't they shown for all variables? Where are the "supplementary notes" that we need to interpret Figure S3?
L884: That makes no sense, then use the closest gridcell that is not ocean
L885: Why? Did you check if total GPP was the sum of the PFT GPP? If so you can report it!
L901: please don't use a screenshot for a table … Report instead some summary stats for the different models, which should simplify Table S1 greatly..
Table S2: how do you define "severely"? Before you said it was 15 models, here you report less and perhaps the values not used in the simulations! This makes no sense. Please report the values in the simulations by contacting the persons in charge of those! Please report variability in the field-measured values (CWM and deviations!).

Version 1:

Reviewer comments:

Reviewer #1

(Remarks to the Author)
I am satisfied with how the authors have addressed my comments. However, I have one remaining minor suggestion: In line 65, the text states that satellites underestimate fAPAR. Please replace 'satellites' with 'remote-sensing products,' 'satellite sensors,' or a similar term.

Reviewer #2

(Remarks to the Author)
General comment: Thank for addressing the comments from the reviewers. In my opinion you improved the paper substantially.

Minor comments:
lines 308-320: check formatting
lines 322-324: “LPJ-GUESS was excluded as its documentation suggested against this calculation.” – change “suggested” to “advised” – a reason for this would also be helpful to see. How is it different from the other models in this regard?

lines 408-410: keep the decimals reported for different values consistent

lines 588-590: “At study site KOG, most models overestimate LAI (Figure S7), suggesting that LAI would not explain the underestimation of GPP at this site. At study sites BOB and KOG however, models averaged LAI is very close to field measurements” – Did you mean KOG overestimate, while BOB and ANK are close to field measurements?

Lines 618-625: check formatting

Line 646: “but also explains why this underestimation occurs” – I would suggest a bit of rephrasing as you only explained some extent of the underestimation.

Open Access This Peer Review File is licensed under a Creative Commons Attribution 4.0 International License, which permits use, sharing, adaptation, distribution and reproduction in any medium or format, as long as you give appropriate credit to the original author(s) and the source, provide a link to the Creative Commons license, and indicate if changes were made.
In cases where reviewers are anonymous, credit should be given to 'Anonymous Referee' and the source.
The images or other third party material in this Peer Review File are included in the article’s Creative Commons license, unless indicated otherwise in a credit line to the material. If material is not included in the article’s Creative Commons

license and your intended use is not permitted by statutory regulation or exceeds the permitted use, you will need to obtain permission directly from the copyright holder.
To view a copy of this license, visit https://creativecommons.org/licenses/by/4.0/

Response to reviewers' comments

Please note that your original comments are in green, and our response is in black. Quoting of texts in the revised manuscript is in grey. A light green colour is chosen for visual impairment consideration.

Reviewer 1

The manuscript titled "Why models underestimate West African tropical forest productivity" by Huanyuan Zhang-Zheng and colleagues evaluates how well TRENDY models, MODIS, and FLUX- COM reproduce annual mean GPP when compared against biometric GPP measurements from three sites. Based on the comparison, the authors conclude that the aforementioned products un- derestimate GPP for the three sites. The study then explores three potential sources that drive the model-data difference: (i) incorrect light-use efficiency (LUE) or photosynthetic capacity (max- imum carbonxylation rate Vcmax and maximum electron transport rate Jmax), (ii) incorrect frac- tional absorbed photosynthetic radiation (fAPAR), (iii) inappropriate assignment of plant func- tional types, and (iv) inaccurate climate variables. The results suggests that most of the data-model difference is due to fAPAR and photosynthetic traits. This conclusion is based on comparing simu- lations where the so called P-model is run (i) with satellite-based fAPAR while allowing the model to simulate LUE using optimality theory, and (ii) measured fAPAR and measured LUE that is based on Vcmax and Jmax. The values of the latter two parameters are derived from light-saturated photosynthesis (Asat) and light-and-CO2 saturated photosysnthesis (Amax) field site measurements. The study addresses an important issue in carbon cycle modeling. Any disagreement between TRENDY model output and reference data like MODIS-GPP can be due to limitations of the models, the reference data and, which is most likely, a combination of both. This study tries to identify the sources of model-data discrepancy by integrating two types of models (TRENDY, P- model), global earth observations (MODIS, FLUXCOM), and in-situ measurements (biometric GPP, plant traits). I found the manuscript very interesting, but there is still room for improvement. Based on my review, I recommend that the manuscript may be considered for publication in Nature Communications after major revisions. Please find my comments below.

Response: Thank you for your affirmation and the nice summary.

General Comments

Your study is fascinating, but I would caution from overselling it. Your introduction section emphasizes how unusually high the biometric GPP values are compared to measurements in the Amazon (greatly surpassing values measured in Amazonia). Reading the corresponding

reference shows that the difference is about 20%. Given measurement errors as well as spatial and temporal variability of GPP, I am not sure with how much confident this statement can be made. Personally, I would drop this part and focus on the model-data mismatch for the West African sites, instead.

Response: Thank you for your suggestion, in this revision, we toned down the language to avoid overselling results. For example, following your suggestions, we have deleted the sentence in the second paragraph of introduction.

Also, I would stress that your results are valid for your sights, and that those sites are not necessarily representative for all of West Africa.

Response: Thank you for your suggestion, we are now highlighting the limitation of spatial cover in Conclusion section.

I also don't see why the model-data mismatch shows a lack of physiological understanding underpinning the high productivity. Your manuscript shows that local parameter values reduce most of the model-data discrepancy, without the need of developing a new theory.

Response: You have a great point here, the story did end up as 'adequate' theory but poor representation of traits, we have rephrased it as:

As models are designed based on current ecological theory, such a large discrepancy of multiple sites signals a lack of physiological understanding or poor parameterisation of forest physiology.

You show that TRENDY models, MODIS, and FLUXCOM underestimate GPP when evaluated against biometric GPP. However, in one of the tree sites (ANK) you also have eddy covariance measurements, which are considerably lower (28 MgC ha−1 yr−1 on average) compared to biometric measurements (40 MgC ha−1 yr−1). You are quick to dismiss the validity of the covariance data for a number of reasons that you list. That approach is not convincing. I suggest you either acknowledge that your biometric measurements are considerably larger compared to eddy covariance measurements, and that the differences between biometric GPP and MODIS GPP could be due to both, overestimation of the biometric val- ues and underestimation of MODIS, or, you provide a more detailed analysis that convinces the reader why the eddy covariance data are underestimating the true values.

Response: Thank you, this is a super important point, please see response in the section below.

To fully eliminate the role of classifications, I suggest you use tree-only GPP from TRENDY for the comparison against biometric GPP.

Response: Very good point, now we are using tree-only GPP in Figure 2 for those reported GPP by PFT. We did not remove models without PFT because it is still interesting (and fair) to show these models. We now have very little words dedicated to PFT and PFT relevant figures are in supplementary only. However, we still keep the hypotheses of PFT and climate

variables. Both of them ended up rejected but these are key inputs in models so it is more reasonable to hypothesise them.

Detailed Comments

L1 tropical forest productivity is a vague term. I suggest you write gross primary productivity, instead.

Response: We followed your suggestion, however, reviewer #3 suggested to compare NPP across biometric, TRENDY and MODIS. To address the suggestion with this revision, we are very pleased to add figures and texts showing data-model comparison for NPP. Given that now the manuscript also presents similar pattern on NPP, it becomes less necessary to highlight ‘gross’, the title thus becomes:

Why do models underestimate West African tropical forest **primary** productivity?

L58 Typo (highest Forests)

Response: Thanks

L61 Revise grammar

Response: we have done so, now:

We found that biometric GPP measurements are on average 56.3% higher than multiple global GPP products at the study sites.

L97 You write that The first field quantification of GPP in African forests [...] reported possibly the highest GPP value recorded in intact forests - greatly surpassing values measured in Amazonia and cite your previous paper as a source (Zhang-Zheng, H. et al. Contrasting carbon cycle along tropical forest aridity gradients in W Africa and Amazonia, 2023). In your previous paper you show that the highest GPP value in Amazonia is estimated with about 42 MgC ha^{-1} yr^{-1}, while the largest GPP value in West Africa is estimated with about 49 MgC ha^{-1} yr^{-1}, a difference of about 20%. I think you want to make the case that the differences are larger for sites with comparable rainfall estimates. However, it is not clear from Figure 2 whether the rainfall amounts are comparable, as the horizontal axis lists stations in a qualitative, rather than quantitative, wet/dry order. It may very well be that GPP is larger in West Africa compared to the Amazon, but I think you need to do a better job in convincing the reader that this is indeed the case. GPP is subject to spatial and temporal variability. What convinces you that the differences in West African and Amazonian GPP assessed in your previous paper are not due to limited spatial and temporal sampling? In particular given that the stem biomass of the sites you are comparing are very similar. I don’t intend to critique your previous paper, but the conclusions that you draw from your previous paper are the premise of your present manuscript, which makes them hard to ignore. I suggest you provide a more nuanced and quantitative description of the differences in West African and Amazonian GPP, or simply drop this comparison.

Response: We totally agreed with your views that more explanations are needed to support such a statement, but as you said, the comparison with Amazonian GPP is not very pertinent to the research aim of this manuscript. We have followed your suggestion and dropped this comparison.

L101 You write that It has not yet been explained [...] why this area has surprisingly high GPP. As models are designed based on current ecological theory, such a large discrepancy of multiple sites signals a lack of physiological understanding underpinning the high productivity.

I think you are overstating the problem. According to your manuscript, the mismatch is driven by values of fAPAR, Vmax and Jmax. No new physiological understanding is therefore required or developed in this manuscript.

Response: This is a good point here, the story did end up as 'adequate' theory but poor representation of traits, we have rephrased it as:

As models are designed based on current ecological theory, such a large discrepancy of multiple sites signals a lack of physiological understanding or poor parameterisation of forest physiology.

L117 The list also includes MODIS-GPP, not just vegetation models. Rewrite.

Response: Thanks, we have rephrased it as:

Most vegetation models and global GPP products calculate GPP using key inputs including:

L124 Models typically simulate LAI and then express fAPAR as a function of LAI and canopy extinction coefficient. The key input would be the canopy extinction coefficient.

Response: Sorry for omitting canopy extinction coefficient, we have rephrased following your suggestion.

L140 Your study has 3 objectives and 4 hypotheses. You use different models and data sets to address your four objectives, and objective three will be tested using 4 different hypotheses. I suggest you enhance the clarity of your approach by providing a figure or table that illustrates the connection between objectives, hypotheses, data sets, and models.

Response: Yes you got the study structure perfectly! We have refined the text at the end of the introduction to enhance clarity. We have also reworked figure 2 to show the connection between objectives, hypotheses, data sets, and models.

L150 Please provide at least a brief summary of how biometric GPP is measured. I would also outline some of the advantages and disadvantages when compared to other approaches, such as the eddy covariance method.

Response: We have provided more description of biometric GPP. We have added the advantage and disadvantage in discussion at the point where we compare biometric GPP to flux tower at study site ANK. See first and second paragraph of discussion.

L155 Revise grammar

Response: We have done so.

L166 You have not yet defined V_{cmax} and J_{max}

Response: Sorry, we happened to define them in later texts, we have corrected this.

L195 Using the term models for vegetation models, P-models, FLUXCOM and MODIS is confusing. I suggest you use a terminology that distinguishes among them. I agree that MODIS products contain a modeling component, but I would still refer to MODIS as a satellite-based product rather than a model. You could use the term observation-based reference data set for MODIS and FLUXCOM.

Response: Thank you for your suggestion, you are right that different terms should be used to distinguish TRENDY, Fluxcom and MODIS. However, “data set” may cause confusion with Biometric GPP. You mentioned “GPP products” above which we have adopted to refer to FLUXCOM and MODIS. Now we say:

FLUXCOM and MODIS, by contrast, are observation-based GPP products that do not depend on the FvCB model.

L227 Revise grammar

Response: We have done so.

L230 You write that Pmodel calculates a LUE from Jmax before calculating GPP. Why are you mentioning this? Either delete or elaborate.

Response: we have deleted this sentence.

L243 Replace *plants functional types* with *plant functional types*

Response: we have done so.

L243 Replace *climate variables* with *biased climate data*, or something along those lines

Response: thanks we have done so.

L245 Add the article *the* before Pmodel, here and below

Response: thanks we have corrected the article for Pmodel throughout.

L257 Why do you expect the differences to be slight? Rather than just referring to Figure S3, tell the reader how much of a difference you expect and why.

Response: thanks, now we have managed to retrieve MERRA-2 PPFD from GMAO, which should be the PPFD used by MODIS (if not MERRA-1). So we don't need to 'expect the difference' here but we can show the reader the actual difference. We have updated this paragraph and point to the new figures.

L262 Move this up to L247 where you discuss the topic first.

Response: This line is now deleted, because we have managed to download PPFD data from GMAO.

L274 Perhaps better to write *misclassification of land cover* rather than *confusion of land cover classification*

Response: good suggestions we have done so.

277 Replace *figure* with *Figure*

Response: thank you we have done so.

L290 It would be nice if you could briefly iterate Objective 3 and Hypothesis
4

Response: thanks, we have done so.

L290 Did you already define c_i/c_a?

Response: Thanks for your reminder, this term is now defined at line 215.

L296 ERA-Interim was replaced by ERA5 in 2016. Why not use ERA5, instead?

Response: Good suggestion, it turns out that, you are right, ERA5-land did better than ERA-interim. Probably because it has much better resolution, ERA5-land can distinguish differences between the sites well. ERA5-Land also out-performs MERRA-2 (belongs to GAMO, used by MODIS), see new Figure S3.

L300 Compared to what other data products? Also, I would replace the term *best* with language more common in statistics, such as *most accurate*, or *least biased*, or *performs best across a range of statistical metrics*, depending on how the analysis was carried out.

Response: we have now rephrased following your suggestions, the sentence now reads:

Previous data-model comparisons of climate variables for tropical forest sites47 found ERA-interim outperform Climate Forecast System Reanalysis (CFSR), MERRA2 and the Japanese 55-year Reanalysis (JRA55) for Africa.

L321 Please choose a different way to start this subsection. Also, use present-tense if the flux tower still exists.

Response: we removed this sentence and went straight to explain FLUXCOM. The tower is still there (now with Phenocam on) but the eddy covariance device died in 2014. We have moved the discussion associated with the flux tower to 'Discussion'.

L326 The S2 simulation keeps the land cover state fixed to its pre-industrial state of 1700. Please mention this. I imagine that the fraction of crops in the grid cells is therefore much smaller compared to the current state. That is something you may want to mention when explaining Figure S4 where you compare forest-only and whole-gridcell GPP.

Response: Thanks for your suggestion, now in Method, we say:

For TRENDY, we analysed the model outputs in version 9 35 under the S2 protocol, in which climate and CO_2 change while the land cover state is fixed to its pre-industrial state of 1700.

Now in discussion, we say:

TRENDY S2 keeps the land cover state fixed to its pre-industrial state of 1700 and thus the proportion of C4 grass (Table S1) is temporarily invariant and could be smaller than a land cover dynamic simulation.

L332 Replace *pixel* with *gridcell*

Response: we have changed this throughout the manuscript.

L372 Typo

Response: thanks for your detailed check, we have replaced FIGRUE with FIGURE.

L386 Replace *DGVMs models'* with *DGVMs'*

Response: we have done so.

L414 Well, the in-situ fAPAR measurements are fairly constant across months, they don't really have a peak, except perhaps for KOG. I would write that *field fAPAR remains fairly constant during the year.* and then deleted the next sentence.

Response: we have done so.

L421 Add that you are referring to Figure 4a.

Response: we have done so.

L439 I don't find this section very convincing. First, your argument that GPP in forests and adjacent croplands should be equal is based on a single study that evaluates NPP, rather than GPP. I would be careful with extrapolating their conclusions to GPP and to take it for

granted that their conclusions also apply to your sites. What if the adjacent cocoa plants have not yet reached maturity? What if management practices differ for your site? Also, there are considerable differences between TRENDY GPP computed for the whole grid cell versus tree PFTs only (Figure S4, e.g. ORCHIDEE-CNP). There is of course a very easy solution to this. You could base your study on GPP computed from forest-only PFTs rather than grid-cell level PFTs, which ensures you are comparing apples to apples.

Response: Thank you so much for providing the 'easy solution', we have done so. Now the Figure 2 has been changed, with new captions as shown below. We still include models that do not report GPP by PFT.

'For TRENDY models that reported GPP per plant functional types, we display forests-only GPP'

We have rewritten this paragraph to reflect this change:

At BOB the entire forest fragment is smaller than a single grid cell in a DGVM but is hundreds of times larger than a MODIS gridcell (scales illustrated in Figure S 1). MODIS GPP estimates that the surrounding area (a composite of savanna and cocoa farms) has only half the productivity of the forests. Therefore, the mean of the whole map (19.6 MgC/ha/year) appears to be smaller than that of the forests (23 MgC/ha/year). This is not the case for TRENDY, which simulated a comparable value between forests-only and whole grid cell GPP (Figure S4) (i.e. similar simulated GPP between forests and C4 grass). There are no field studies to our knowledge comparing cocoa farm GPP to adjacent forests GPP.

For all the study sites, most TRENDY models correctly simulate the grid cell as a composite of 'forest' or 'C4 grass', although the fraction of forests varies considerably among models (Table S1). Given that the 'forest-only' GPP of TRENDY and MODIS are much lower than biometric GPP (Figure 2; S4; S1) we conclude that the GPP data-model discrepancy between TRENDY and biometric GPP cannot be explained by scaling or PFT issues (rejecting hypothesis 3). Besides, please note that TRENDY S2 keeps the land cover state fixed to its pre-industrial state of 1700 and thus the proportion of C4 grass (Table S1) is temporarily invariant and could be smaller than a land cover dynamic simulation (e.g. MODIS replies on satellite imageries that incorporates real-time land cover change).

L457 I suggest you briefly iterate the hypotheses at the beginning of each section.

Response: yes we have done so.

L463 No need for bold font

Response: we have done so.

L490 You write that *the biometric GPP is an average across multiple plots spanning several years, and its high productivity is supported by another field study using similar biometric method of GPP quantification* 47. Reference 47 refers to the study by Morel et al (2019), which reports on NPP, primarily in coca plants. I did not find any mentioning of GPP in Morel et al (2019). Why does their study support your GPP measurements?

Response: Deep apologies, we have corrected this mistake, it now reads:

The biometric GPP is an average across multiple plots spanning several years. To our knowledge, there are no other biometric GPP measurements in the study region, but the high productivity is supported by another field study reporting biometric NPP 49;

L505 I would mention this already in the introduction section.

Response: now this paper is mentioned in the first paragraph of introduction: For instance, many previous studies found particularly large GPP data-model discrepancy for tropical forests between flux towers and models (including satellite GPP products) 16–19

L520 This is an important point, but it is not entirely true and requires a bit more discussion. Traditionally, the maximum carboxylation rate vcmax is a PFT-specific constant. So if the true vcmax differs among sites due to nutrient availability, then models cannot reproduce those patterns. However, some models simulate vcmax as a function of nutrient availability, such as nitrogen, in which case they could, at least in theory, reproduce those patterns. At the same time, at least in my experience, prescribed vcmax values generally lead to more realistic GPP values as simulating the N cycle has its own set of challenges.

Response: This explanation is much better, we have incorporated your text as:

This substantial spatial variability of V_{cmax} has not been incorporated in most TRENDY models 29,62 which could lead to underestimation of GPP at such regions. Traditionally, V_{cmax} is a PFT-specific constant. If the true V_{cmax} differs among sites due to nutrient availability, then models can only embody the dynamics with varying PFT fractions. Some models do simulate V_{cmax} as a function of leaf nitrogen, in which case they could, at least in theory, reproduce those patterns. Nonetheless, leaf nitrogen may be simulated also with PFT-specific parameters and the N cycle has its own set of challenges. Thus further modelling study is needed to carefully consider the high V_{cmax} at this region.

L527 Please mention that v_{cmax} model parameters differ to measurements *compared to the mea- surements at your sites*. The values may still be appropriate at a global scale, as in-situ v_{cmax} measurement values differ considerably among sites.

L532 Well, the large v_{cmax} parameter value range is consistent with the wide range present in the TRY data base.

Combined response: great points, the above two points have been added, it now reads:

Further, although Vcmax model parameters may differ from measurements at the study sites, the values may still be appropriate at a larger scale, as in-situ Vcmax measurement values differ considerably among sites. The wide range of Vcmax actually reflects substantial variation in global traits databases, where further data-model benchmarking study is suggested.

L551 Please replace *dramatically* with more neutral language.

Response: We have replaced ‘dramatically’ with greatly.

L486 Do eddy co- variance measurement sites in the region report values comparable to your biometric GPP values? After reading further I now see that eddy covariance data estimate GPP in ANK with about 28 rather than 40 MgC ha−1 yr−1, which does not support your high GPP values.

L552 Further above (L486) you make the case that the difference between biometric GPP and values from MODIS and FLUXCOM are due to an underestimation of MODIS/FLUXCOM rather than an overestimation by the biometric GPP. The eddy covariance measurements that you cite here however indicate that the true GPP values could be lower than your measure- ments. It is very important that you consider all available evidence without prejudices, so please add this part to the discussion in L486. To iterate, the biometric measurements in ANK, for which you have eddy covariance data, are 40 MgC ha−1 yr−1. The corresponding value from the eddy covariance measurement site has a mean value of 28 MgC ha−1 yr−1. This is the same value as FLUXCOM (naturally, since the site is likely used as an input for FLUXCOM), and many TRENDY models are not far off. It is not convincing to sim- ply dismiss this eddy covariance measurement site. I suggest you either acknowledge that your biometric measurements are considerably larger compared to eddy covariance mea- surements, and that the differences between biometric GPP and MODIS GPP could be due to both, overestimation of the biometric values and underestimation of MODIS, or, you pro- vide a more detailed analysis about comparing the eddy covariance measurement site and your biometric values. For instance, reduce the comparison to the same years or months of measurement.

Response: Thank you, this is a very important point. We tried both of your suggestions. (1) We acknowledge that it is possible that biometric GPP overestimates GPP at site ANK, especially given the ‘unresolved discrepancy’ for site ANK in Figure 4,. (2) We had a close look at the eddy-covariance tower, and the data seem to be very patchy. We now provide one more figure (see below) about the eddy-covariance tower in the supplementary. The eddy-covariance data reported more complete data in 2011 (36 MgC/ha/year) than 2012 and 2014. Thus the 36 MgC/ha/year is more trustworthy. Overall, the true GPP appears to be more likely to situate around 36 MgC/ha/year. We are now acknowledging that the biometric GPP at site ANK may overestimate GPP and thus the ‘unresolved discrepancy’ could be due to bias in the biometric GPP.

Now reads:

(4) There is only one flux tower for West African forests (GH-ank at the Ankasa site), which reports three years of GPP varying substantially 19. The tower reported 2011 annual GPP at 36.06 MgC/ha/year, but the tower only operated for half-year in 2012 and 2014 with estimated annual GPP at 22.02 and 26.1 respectively (FLUXNET201565, variable GPP_NT_CUT_REF) (Figure S 8). In comparison, the biometric GPP is 40.11 and Pmodel_PfL is 31.85. Therefore, the ‘true’ GPP of site ANK appears to be more likely to situate around 36 MgC/ha/year with biometric GPP overestimating, and the ‘unresolved data-model discrepancy’ at site ANK (Figure 3) is probably due to bias in the biometric GPP. Many tropical flux towers face logical or technical issues 67,68, and challenging subcanopy CO2 storage estimation 69,70. For

example, the road leading to site ANK is seasonally inundated (Photos in Supplementary). During FLUXCOM extrapolation, the machine learning algorithm would not receive information about BOB's higher photosynthetic capacity and would simply predict BOB less productive than ANK (opposite the observed GPP pattern) due to the lower precipitation received at BOB (Table S3).

L562 Spell-out GLASS

Response: a detailed look at GLASS documentary reveals that this is also a satellite based product without incorporating field measurements (https://www.sciencedirect.com/science/article/pii/S0034425722000992). Thus it is not appropriate to be used as an example here. We have removed this sentence.

L572 Typo

Response: sorry, we have changed HOULD to SHOULD.

L575 Well, but this is perfectly fine because a model gridcell contains fractional PFT coverage, so the comparison with a resampled product is fair.

Response: yes true, this was a trivial suggestion, now we say:

Additionally, West African forests are highly fragmented 74 (Figure S 1), so future studies across scales are suggested to check plant functional types assignment (Figure S 4) 49.

L583 You mean they vary considerably *among sites*? Also, there is a typo (like). Here is also a contradiction. If GPP varies considerably among sites, then those values are not necessarily representative for all of West Africa. How then can you conclude that almost all TRENDY models underestimate West African GPP? I would use more cautious language.

Response: we have deleted this sentence. The spatial variation of biometric GPP does not imply anything about models. To improve, we have extended our investigation of TRENDY following Reviewer 3's comments. We are now providing data-model comparison for leaf area index (Figure S7). We have also expanded Table S2 to link Vcmax values with TRENDY models. The new results reinforce the conclusion and suggest that the causes of data-model discrepancy may vary among TRENDY models (because some models overestimate while others underestimate Vcmax and LAI values) (Figure S7, Table S2).

L586 Well, in the nutrient parameterizations I am familiar with, vcmax is expressed as a function of nutrient availability. So the discussion on nutrients is relevant in this context.

Response: thanks, the original sentence was not meant to say that nutrients discussion is trivial, but further study is needed to better represent Vcmax or incorporate nitrogen limitation, now the sentence becomes:

Further, we identified Vcmax as a key factor. Numerous models calculate Vcmax from leaf nitrogen but nitrogen coupled models do not contrast with carbon-only models at the study

site (Figure 2), suggesting that the nitrogen-GPP relationship still has room for improvement in future modelling studies.

L578 Please use consistent notation (Pmodel or P-model)

Response: We have corrected this throughout.

L587 Typo (mode')

Response: Thanks we have corrected this.

Figures

Figure 1 The figure gives the impression that there are hardly any fAPAR data at the locations of the sites as these loctions are all strongly affected by cloud cover. Is that so? Also, I appreciate panel (b) as it is intuitively easy to locate your study area, but I don't see any additional information in panel (a). Perhaps zoom in even closer if you want to show more details, or delete panel (a).

Response: Yes, exactly, there are hardly any fAPAR data at the locations of the sites, during the rain season (August September), but there are patchy data in the dry season (see Figure 4). Now we have zoomed in closer for panel (b) and zoomed out to see most of the Africa continent for (a).

Figure 2 CLASSIC and JSBACH are missing in panel (a), how come? To my understanding, the TRENDY GPP data shown here consist of the gridcell value. Please replace this with the GPP associated with tree PFTs since your measurement sites only contain forest and no crops or grasslands. Also, I assume that the GPP values of insitu data, TRENDY models, MODIS data and FLUXCOM data are all based on the same period, right? Please add the corresponding period in the figure caption or the figure itself. Finally, I would add error bars that show the inter-annual variability.

L284 Why do you do the comparison to the grid cell average? Why not compare the forest-only GPP from models against the GPP from field measurements?

Combined response:

For missing CLASSIC and JSBACH in panel (a): nice spot on, we had a detailed look at these two models. It turns out that, study site ANK is about 28 km from the shore. However, in these two models, ANK's coordinate falls on a pixel of sea, thus the GPP of this pixel could not be reported.

For comparison with grid cell average versus forest-only: Ah a very good point, there are less models in figure S4 (forest-only) than Figure 2 (grid cell average), because some models did not report GPP per PFT, or the PFT modelling approach was not used in those models. It does not sound reasonable to omit these non-PFT models in Figure 2. Thus, we have revised figure 2 to display 'forest-only' GPP for models that reported GPP per PFT. We still display whole pixel GPP for other models and other GPP sources.

For the time period, this is a great point, our FLUXCOM and TRENDY were 10 years average in the last version. Now we have modified the calculation to ensure fair comparison with biometric GPP. Now all variables (from GPP to climate) are all shown as a mean annual GPP across 2011 to 2016.

For the error bar, we have added maximum and minimum mean annual GPP during the study period.

Figure 3 Show how each experiment relates to the different hypotheses

Response: This is now incorporated in the new figure 2.

Figure 4 I would include Pmodel_null here as a reference and to explain the difference between MODIS and Pmodel_P in site KOG, which, to my understanding, is caused by using site- level PPFD.

L424 I suggest you include *Pmodel_null* in Figure 3 to assess the role of PPFD. This way the reader is not forced to jump to Figure S3.

Response: Thank you again for your suggestion. We just put Pmodel_null in Figure 3 but it is a bit difficult to colour the difference bar. We really appreciate that you have a perfect understanding of the manuscript but the convoluted Figure 3 may confuse future readers especially those without modelling background. Now we have a full image (including Pmodel_null) as Figure S6. As you anticipated, PPFD explains a small portion of data-model discrepancy in KOG, but not in BOB and ANK. However, given the new figure, PPFD is not a key factor behind the data-model discrepancy, we thus leave this figure as Figure S6. Please do let us know if you prefer figure S6 as the main text figure. Thank you again for writing a high-quality and in-depth review. Your suggestions are very reasonable and helpful.

Overall response: thank you again for your detailed comments and constructive suggestions.

Reviewer 2

Key results

In response to several indications of high uncertainty in estimates of forest productivity, specifically for West Africa, the authors explored possible reasons for GPP data-model discrepancy. Addressing three objectives, the authors compared biometric GPP to various models and satellite-based products, interrogated the consistency of in situ high productivity to leaf photosynthetic traits and field observations usually simulated by models, and substituted model parameters with field observations to evaluate the data-model discrepancies. This was done at three sites with different forest structure and along a rainfall gradient in Ghana. Results indicated that GPP models generally underestimate the GPP when compared to the field measured data. They also identified that fAPAR and LUE from satellite

products are mostly responsible for the data-model discrepancies and increasing field observations to inform models may be required to reduce uncertainty in model estimates.

The study has wide significance to the global modelling community, especially for carbon cycles, and highlights the need for increased field observations in forest areas in Africa that is highly recognised. This may even be true beyond forests and the results may extend to other ecosystems, thus pointing out that increasing model accuracy may require investigations like this to be rolled out more extensively.

Response: Thank you so much for your affirmation.

Validity

The data interpretation and conclusions are valid and the authors covered the important aspects of the topic. They also point out the limitation of their results.

Significance

The results of the study validates what has been implied by many studies that used GPP models. It also narrows down some of the possible causes of model discrepancy. Although the study was conducted at a local scale it certainly has important implications for various global and regional models.

Data and methodology

The methodological approach is logical and comprehensive and the data appears to be of good quality. A description of the equipment used for field measurements may be useful.

Analytical approach

The analytical approaches are valid and sufficient to test the hypotheses.

Suggested improvements

1. In line 478 the authors mention “possible bias in field observations” – perhaps they could be more explicit about what such bias would entail.

Response: yes, now we have rephrased this sentence and make it more clear. At the revision, we also discuss the flux tower at site ANK, and now we acknowledged that the ‘unresolved discrepancy’ at site ANK is more likely to associate with bias in biometric GPP. Now the sentence reads:

but leave a modest, though greatly reduced, unresolved data-model discrepancy still persisting at ANK, which may be caused by inaccurate traits measurements, photosynthesis model assumption (e.g. the big leaf assumption) or possible bias in field measurements of the biometric GPP (e.g. the upscaling of leaf and stem respiration; the estimation of roots exudation) 50.

2. As far as I can tell from the manuscript, the authors compared MODIS fAPAR data averaged over 2001-2020 to field data averaged over 2012-2017. This does not make sense as it introduces environmental variability that is not accounted for by the field data collection period. Could the authors address this or clarify. Perhaps being more clear about the observation periods of the different data sources in the main methods section could also be useful.

Response: Apologies for this mistake, the field data span from January 2011 to the end of 2016. Now we have modified all models and satellite variables to an annual mean across 2011 to 2016. We have changed the main text and figures captions accordingly.

3. Typo line 708: “uses” should be “used”

Response: we have corrected this.

4. Typo line 709: “had” should be “have”

Response: we have corrected this.

5. Typo line 726: “above –anopy” should be “above-canopy”

Response: Sorry for the typo, we have changed it.

6. Lines 950-954: the season description is repeated twice

Response: Sorry we have deleted the repetition.

7. Lines 965-966: I was wondering what the logging status of BOB-03 and BOB-04 were?

Response: Apologies for omitting BOB03 and BOB04. Now we have double checked the logging history and changed this sentence:

BOB-01 is an intact forest (baseline conditions plots); BOB-02 to 04 were lightly logged (2-3 stems/ha extracted) in 1959. BOB-05 and BOB-06 were logged in 2001 (2-3 stems/ha extracted).

Clarity and context

The manuscript is clearly written and it is easy to follow the methodology and approaches. The study also provided sufficient and relevant background and created good context that highlights the importance of the study.

References

The references are appropriate and relevant.

Overall response: Thank you again for your affirmation and support.

Reviewer 3

Main comment

The study by Zhang-Zhen et al. seeks to explain why models currently underestimate West African tropical forest (gross) productivity, which is a crucial question for the global carbon cycle. Especially given the importance of tropical forests and the fact that African (rain)forests have been long neglected in terms of observations (and hence data constraints for models). This study is based on previous (and amazing!) findings of very large GPP in multiple forest sites in Ghana, and builds on a dataset that was recently published in the very same journal.

The study starts by comparing field-based observations of GPP with multiple model (types): DGVMs with Trendy v9, and remote-sensing (RS) estimates.

Response: Thank you for your support.

I was surprised to see that the RS products exactly correspond to what was used in the previous study and hence by the overlap with an existing manuscript. The authors then dive into the reasons why the models and the observations might diverge. They designed a series of experiments to explain the mismatch between observations and simulations. They show very nicely how underestimated fAPAR biases the estimates of GPP as well as the importance of locally measured field traits like Vcmax.

Response: Thank you for your support. Indeed, there is a little bit of overlap with our previously published paper, on FLUXCOM and MODIS (MOD17A3HGF product only) (https://www.nature.com/articles/s41467-024-47202-x).

In this study, we investigated MODIS further and looked at MODIS fAPAR and GPP with and without gap-filling. Following your suggestions, in this revision, we now make data-model comparisons for NPP and LAI. We also investigated TRENDY and Pmodel which were not involved in our previous paper. Thus the data-model comparison part of this paper is much wider and more thorough than the previous one, especially with the amendments that you suggested. This study also investigates the possible causes of GPP discrepancy which was not done in the previous paper.

However, there is almost no attempt to explain why DGVM might underestimate GPP. The authors looked at the distribution of traits in the models but they could not trace back the values that were actually used in the simulations (they are indeed not reported but a simple

email to the different model contributors should have been sufficient to get those values). Also, Trendy is forced with crujra (if I remember well) but there was no attempt to check biases and uncertainties in those drivers compared to locally available ones nor with simulated LAI. I believe adding this piece somehow would greatly improve the quality of the study. In short, I think the authors managed to achieve objective 1 (but some overlap with a previous study, which might be problematic in editor's eye), objective 2 (the biggest finding of this study) but only partially objective 3 which is the core (and the title) of the paper. They managed to explain the discrepancy between MODIS and observations with the underestimated fAPAR (which is per se not mindblowing as everybody knows the cloudiness in the tropics should make users cautious) but did not explain the mismatch between Trendy and observations (nor tried to do so), which was very disappointing.

L522: To me, if you are not 100% confident which Vcmax values were used in the actual simulations, that discussion makes no sense..

Response: Apologies for the disappointment and thank you for the solutions that you have provided. Now we have made a data-model comparison across MERRA-2 (used by MODIS), ERA5-Land (high resolution), and CRUJRA (used by TRENDY DGVMs), following your suggestions (Figure S3). This comparison further backs the conclusion that climate variables are unlikely to explain GPP data-model discrepancy.

Thank you for your suggestion on LAI; the new figures on LAI provide key information (Figure S7). At study site KOG, most models overestimate LAI, suggesting that LAI would not explain the underestimation of GPP. There may be a large discrepancy in photosynthetic capacity. At study site BOB and KOG, models averaged LAI are very close to field measurements, with some models overestimating and others underestimating LAI, suggesting that the key parameters accounting for GPP underestimation at BOB and ANK should vary depending on model. New lines in Discussion are added to explain this.

For traits (Vcmax), we have contacted several modellers (including TRENDY coordinators Prof Sitch). We were told that unfortunately, Vcmax was not written out during TRENDY v9 simulation. They may write out more variables (including Vcmax and leaf nitrogen content) in future TRENDY simulation to enable more model diagnostics. Regarding reproducing Vcmax for DGVMs, (Rogers., 2014) and (Rogers., 2017) have made this effort. Given that, many parameters behind leaf nitrogen content and photosynthetic traits are still PFT-specific, Vcmax values in (Rogers., 2014) still represent the best guess of the Vcmax behind TRENDY v9 run.

Some model documentaries provide equations for calculating Vcmax from leaf nitrogen content. For example, JULES (cite) and LPJ-GUESS (cite). They did report vegetation nitrogen content but too much assumption and parameters are needed to convert vegetation nitrogen content to leaf nitrogen content. We feared that going down this route we risk mis-representing Vcmax and would not represent a Vcmax better than (Rogers., 2014) , who has incorporated many personal communications with modellers. We therefore

(1) Rephrase objectives 3 as 'We attempt to account for the data-model discrepancy of MODIS and Pmodel (Table 2) and explore key parameters in DGVMs.' We toned down the language here to recognized that the investigation into TRENDY is just exploratory not accomplished

(2) Improve Table S1 to link photosynthesis submodels in A.Rogers with their parental TRENDY model. We also added data-model comparison for LAI. As LAI and Vcmax are two key variables behind DGVMs GPP, this new set of results suggest that the cause of data-model discrepancy likely varies among DGVMs.
(3) Acknowledge the limitation of our investigation into TRENDY. We are not trendy modellers and we believe that one of the TRENDY modellers could do this Vcmax data-model comparison better. We thus welcome modellers to contact us and collaborate to investigate further. The field measured data are open shared through SpringerNature ‘figshare’ platform. We also shared field-measured leaf nitrogen and leaf phosphorus and soil nutrient in Table S3, should modellers be interested in checking Vcmax and leaf nitrogen values.

Finally, I found the manuscript sometimes hard to read and the figures of rather poor quality. I think its structure could be significantly improved, the figures enhanced, and the text very much polished (some sections, especially in the supplementary, need to be completely rewritten in my opinion).

Response: Apologies again for the disappointment. During this revision, we have corrected many typos and revised the large body of the main text. We have updated almost every figure according to reviewers’ suggestions. We will also provide high resolution figures adhering to the journal’s policy.

To enhance clarity of the study, we have designed a new table (Table 1) to link study objectives, hypotheses with corresponding datasets.

Thank you for your suggestions. At many places we refer to a term or figure repeatedly or with the wrong order. We have corrected accordingly. For supplementary, we have rewritten a large part of it and improved the formatting, following your specific suggestions below.

Other minor comments

I think you could make the abstract more appealing by adding some of the numbers you found: e.g. L61 “higher” but how much?, L64: underestimate (by how much?), etc. Those numbers would give the significance of the discrepancies.

Response: great suggestions! We have added some numbers.

We found the in situ GPP measurements are on average 56.3% higher than multiple global GPP products at the study sites.

Satellites systematically underestimate fAPAR (33.9% on average at study sites) due to cloud contamination issues.

Wouldn’t it be interesting to also look at NPP as all those models that should also report those values (it is unlikely that the models would capture NPP while being so wrong about GPP but you never know: they might be right for the wrong reasons, which would reinforce your findings!)

Response: this is a very good point, we have added NPP, and as you have anticipated, models underestimate NPP as well. We mention this figure in the introduction.

Since GPP is the source of carbon and energy for terrestrial ecosystems in Dynamic global vegetation models (DGVMs), the data-model discrepancy in GPP propagates into NPP (Figure S5) and possibly other downstream variables.

L52: works → work

Response: we have done this.

L86: the seasonality is also a bit question in tropical forests. And while it is a great aspect of your field data, you don't try to benefit from it, why? Wouldn't it be interesting to check how wrong the models are in terms of dynamics? Would also reinforce your point about clouds and fAPAR for MODIS, I suppose.

Response: Yes a very good point, we now highlight the seasonal pattern of fAPAR in results:

A further investigation into MODIS fAPAR (Figure 5) shows that during the rainy season, MODIS fAPAR decreases along with increasing cloud cover (up to 90% of pixels), whereas field fAPAR remains fairly constant during the year. Filtering fAPAR values according to cloudiness or gap-filling of bad data points would remove most of the values from the rainy season, leading to an underestimated mean annual fAPAR.

However, we don't have seasonal biometric GPP for now. We are trying to compile such but we haven't finished. There are some complexities for example, stem NPP is an annual measurement. We need to bring information from the dendrometer band. We are open to future collaboration to pursue a data-model comparison on seasonal patterns.

L87-88: This sentence reads weird, I suggest rephrasing

Response: Now rephrased as:

Multiple global-scale forest GPP studies have indicated that tropical forests have larger uncertainty compared to other biomes

L98: too much detail for the intro

Response: We have deleted this explanation.

L104: Or a mismatch in the drivers of photosynthesis?

Response: Yes you are right, now we say:

As models are designed based on current ecological theory 23, such a large discrepancy of multiple sites signals a lack of physiological understanding or poor parameterisation of forest physiology.

L134: first reference to a figure is to Figure 3… The order of the figures should be corrected!

Response: Sorry, now at this sentence, we refer to the new table made in this revision (Table 1). Now the first figure mentioned is figure 1 in the first paragraph of Method.

L168: reference to an experiment that was not yet described (PfL)

Response: Sorry, to avoid this, now the paper says:

We used these Vcmax and Jmax in one of the Pmodel experiments for Objective 3.

L184: this is not my reading from Zhang and Ye and certainly not the most up-to-date list of GPP products … The authors need a better reason to select those specifically..

Response: Apologies for this confusion, Zhang and Ye did not rank models by popularity, we are citing Zhang and Ye to point readers to different methods and groups of GPP products. The 'widely used' is from the number of citations as to our knowledge, there are no assessment of the popularity of GPP products. We have rephrased this sentence as:

The above choices are widely used models and GPP products that applied distinctly different approaches to calculate GPP [16](Table 2).

L185: Trendy version? Why use version v9 while now v12 is available?

Response: We did this analysis in 2022 when v12 is not available. Yes you are right, it would be ideal to use the latest TRENDY, but, to do so, one would need to request data, download and rerun processings (GPP, NPP and LAI) which will take a long time. It is not likely that TRENDY v12 has resolved the large data-model discrepancy presented in Figure 2 (other figures do not involve TRENDY) because most of the participant models of TRENDY v9 are still in v12 and no fundamental change was made to these models. Thus we fear that the improvement from v9 to v12 may not be worth the effort in this revision.

L208: rpmodel already mentioned earlier.

Response: We have removed the mention here to avoid repetition.

L228: Asat AND Amax, remove "see equations…" already mentioned earlier

Response: we have done this.

L232: weird phrasing… Do you need to strengthen confidence in the field estimates? I suppose this was discussed at length in the previous paper but here it comes out of the blue

Response: We have deleted 'strengthening confidence in the field estimates'. Yes this is discussed more in-depth in the discussion section of this paper so it is redundant here.

L240: remove "also see introduction"

Response: We have done this.

L240 and following: this is repeated from the intro

L243-244: rephrase

Response: Sorry, we have removed this repetition (from 240 to 244).

L257: slight → small

Response: Now this sentence has been removed (the paragraph is rewritten following Reviewer 1's suggestion).

L285: Which Trendy scenario was used? → It is given but way too late → reorganize the methods!

The structure of the methods could be better. It is currently messy and hard to follow. Trendy and MODIS for instance are referred to many times before it is explained what they are exactly.

Response: Following your suggestion, we improve the structure of Method and now explain TRENDY MODIS and FLUXCOM before the experiment's design. We have also made Table 2 which summarised these GPP products.

L299: Does it make sense for Trendy? I suppose you should check the actual Trendy drivers..

L301: "see results" is useless

Response: Thank you for your suggestion, you are right, TRENDY used CRU-JRA instead of ERA-interim. With this revision, we have done an extra body of work with climate variables. We compare across field measurements, CRU-JRA, MERRA-2 (used by MODIS) and ERA5-Land (high resolution, and recommended by previous data-model comparison) (Figure S3).

We have rewritten this paragraph to reflect the above change. Now following your suggestions and revision, the conclusion that climate variables do not explain GPP data-model discrepancy is much stronger.

L312: Why talking about Trendy in the MODIS subsection? Plus it was already explained..

Response: Sorry, we have removed this sentence.

L321: shouldn't the fluxtower data be used for objective 1?

L551-L552: why not report the fluxtower data with the other estimates of GPP?

L552-553: this (values of the fluxtower) belongs to method

Response: We have retrieved more details of the fluxtower data during this revision. The tower reported 2011 annual GPP at 36.06 MgC/ha/year, but the tower only operated for half-year in 2012 and 2014 with estimated annual GPP at 22.02 and 26.1 respectively. Objective 1 compares biometric GPP to various models and GPP products thus it does not involve the fluxtower data. Comparing the fluxtower data to biometric GPP would entail more careful discussions. We have now done so with 14 lines of text in Discussion (4.2)

L334: Did you average over 20 years? Why not take the monthly values and also evaluate the seasonality/interannual variability?

Response: Yes the last version averaged over 20 years. Thank you for your comment, we have now corrected this and reported a mean over 2011 to 2016 to align with field measurements. We have also visualised interannual variability with an error bar in Figure 2.

~L380: Comparison with RS was already reported … What about the model ensemble mean?

Response: Thanks, now we have dedicated more words to model ensemble means.

L392: Do you mean the mean discrepancy across sites?

Response: Apologies for the confusion, we meant to say

The discrepancy between biometric and MODIS GPP is about 20 MgC/ha/year regardless of sites

L399: ‘short of biometric GPP’ is a weird formulation

Response: now rephrased as: but remains lower than biometric GPP

L414: the “peak” is rather flat (at least with the axis chosen for Figure 5), either quantify the increase in the data or reformulate

Response: Yes you are right, we have reformulated this statement:

A further investigation into MODIS fAPAR (Figure 5) shows that during the rainy season, MODIS fAPAR decreases along with increasing cloud cover (up to 90% of pixels), whereas field fAPAR remains fairly constant during the year.

L418: It would be good to add fAPAR with cloud filtering/gap-filtering as well in Figure 5. Your datasets suggest rather constant fAPAR over time (and almost across sites), which is nice and interesting!

Response: Very good point, we have done this. Now the figures show that Both MODIS-no-cloud and MODIS-with-cloud underestimate fAPAR.

L444: but you mainly investigate GPP here… Couldn’t they have similar NPP but very different GPP?

Response: Yes you are right, it is not well-founded to infer GPP from NPP. Now we have deleted this sentence. Following your suggestions, we added a figure on NPP. There are also large data-model discrepancies on NPP. However, we did not investigate farmland NPP which is out of the scope of this paper.

L447: and (not or)

Response: we have corrected this.

L458: but ERA was not used for Trendy…

Response: Thank you for your suggestions above, now we are also comparing field measured climate variables with CRUJRA (used by Trendy) so now we could safely make this statement.

L476: probably better to avoid those qualitative statements (“a modest, though greatly reduced”) and replace them by more objective values.

Response: we have done this, now:

Through this analysis we are able to fully account for the data-model discrepancy at KOG and BOB, but leave part of the data-model discrepancy unresolved (20.5% of biometric GPP) at ANK, which may be caused by

L506-508: If you could quantify this for your region of interest (Figure 1) or even the tropics or refer to studies that do would strengthen your message!

Response: thank you for pointing this out, we realised that it was not appropriate to say West African is more cloud contaminated given the following citations. We are now citing these papers and corrected the phrasing as:

Many tropical rainforests have high persistent cloud cover compared to other forests and therefore may be disproportionately affected by cloud contamination.

Citations: Fensholt, R., & Proud, S. R. (2012). Evaluation of Earth Observation based global long term vegetation trends - Comparing GIMMS and MODIS global NDVI time series. Remote Sensing of Environment, 119, 131–147. https://doi.org/10.1016/j.rse. 2011. 12.015

Sudmanns, M., Tiede, D., Augustin, H., & Lang, S. (2020). Assessing global Sentinel2 coverage dynamics and data availability for operational Earth observation (EO) applications using the EO-Compass. International Journal of Digital Earth, 13(7), 768–784. https://doi.org/10.1080/17538947.2019.1572799

L572: should?

Response: Sorry for this typo.

L585: likely?

Response: we have done so.

Figure 1: rather than a specific day → average frequency of cloud for the studied period (or the rainy season)? Switch subplots a and b (from more global to more local). Why restrict to that specific area? Wouldn't it be nice to see the full "cloud issue" over the tropics?

Response: Thanks for your suggestion, we have done so, and provided a new figure. The figure now zooms out to see most of the African continents but not the whole tropic because the paper focuses on West Africa, and global quantification of cloud contamination has been done in the literature, e.g. (Fensholt & Proud 2012) (Sudmanns et al., 2020).

Figure 2: The dotted purple line is not described, I think? I suggest moving the individual DGVM/RS to supplementary and only keep C-DGVM (mean and distribution), CN DGVMs, All DGVMs, All RS products, P-model (null and _Pfl). The order of the DGVMs changes between subplots which makes the comparison difficult…

Response: Sorry for the change of the order of the DGVMs, it was due to a model simulating ANK on the ocean. Following your suggestion, we picked the most nearby land pixel for ANK. Now all the subplots have the same order of models. The captions are modified to describe the dotted purple line. We appreciate that grouping DGVMs would make the figure neater. However, in this revision, following reviewer 1, we add errors bar to visualise maximum and minimum mean annual GPP. Now this figure shows both interannual range and inter-model variation. Grouping models will lose the interannual range information. We also believe that showing individual models in the main text could attract more readers who are interested in specific models. Thus we are apologising here for not grouping them.

Figure 3: don’t refer to figure 4 in the caption of Figure 3. Wouldn’t it be interesting to test the other scenarios: like measured LUE and fAPAR with ERA-Interim for instance? Just to get the full picture?

Response: We have removed the referring to figure 4. We really appreciate your great understanding of this complicated figure. We fear that adding too many Pmodel experiments may confuse future readers, especially those who are not familiar with DGVMs. Nonetheless, now we add one more figure (Figure S6) to compare Pmodel_null with Pmodel_P which illustrates the effect of GMAO PPFD versus field-measured PPFD, which shows that using GMAO PPFD leading to overestimation of GPP at ANK and BOB but not KOG. In this revision, we also managed to retrieve and add GMAO PPFD (used by MODIS) which makes the investigation more complete.

L711-713: Does this calculation make sense?

Response: we have tried our best to search the literature and consult experts but we could not find better information on combining diffuse and direct fAPAR. Looking at Figure 5, 80% (percentage of cloudy days) is probably a bit too high. However, the diffuse fapar reported by CANEYE software is less than 4% higher than direct fapar, so changing the 80% will not exert observable effect on the calculation of GPP.

L726: canopy → canopy

Response: Apologies again for typo, we have corrected this.

L727: not clear if the outliers are detected at the community or species level → More details: how many were removed?

Response: It is detected at leaf level. We just double-check this calculation. Although we have applied an outlier filter, all values are within the 1.5 IQR so the filter does not remove any value. We had this filter in the first place because back then there were some typos from the field sheet which had been cleaned and corrected. We have removed this sentence and corresponding codes.

Section starting at line 734 should be rewritten and reformatted

Response: Apologies, we have substantially improved the formatting, language and equations of supplementary material during this revision.

L748: remove “we will use this later”

Response: we have done this.

Figure S1: rather than reporting the mean, a density distribution would be more informative

Response: We are very sorry but could you please explain more about ‘density distribution’? apologies that we could not get this suggestion.

Figure S3: what do the error-bars represent? Why aren’t they shown for all variables? Where are the “supplementary notes” that we need to interpret Figure S3?

Response: Apologies for this mistake. We have removed error bars for climate variables (previously they were standard errors which did not provide useful information). ci/ca (leaf

internal to external CO_2 ratio) still has error bars which represent standard error of field measurements (this is a plant trait). We have provided rich text in Method and results to explain Figure S3 (data model comparison for climate variables). We have also provided supplementary note ‘ To reproduce Figure S3’ . Following your suggestion, we have added MERRA-2 and CRUJRA.

L884: That makes no sense, then use the closest gridcell that is not ocean

Response: Apologies, we have done so. Now site ANK has data from all the models - which also solved the problem that ‘ANK had less models displayed than BOB and the x-axis did not align perfectly in the last version.

L885: Why? Did you check if total GPP was the sum of the PFT GPP? If so you can report it!

Response: We should have explained the figure better. The forest-only GPP is not a proportion of whole-pixel GPP, but a potential GPP if the whole pixel was presumed totally forested. Total GPP is indeed the sum of the PFT GPP for LPJ-GUESS. However, the readme file of LPJ-GUESS asked not to calculate ‘forest-only’ GPP if the pixel was presumed to be all forests. Probably because LPJ-GUESS has inter-PFT interaction so such a presumption could not be made?

L901: please don’t use a screenshot for a table … Report instead some summary stats for the different models, which should simplify Table S1 greatly.

Response: Thank you for your suggestion. We have grouped PFT into forests and non-forests. We have replaced this big table with a summary stat table. The journal allows uploading ‘supplementary data’ to attach a big table. We have thus converted the original big table into Supplementary data (instead of a table in supplementary information).

Table S2: how do you define “severely”? Before you said it was 15 models, here you report less and perhaps the values not used in the simulations! This makes no sense. Please report the values in the simulations by contacting the persons in charge of those! Please report variability in the field-measured values (CWM and deviations!).

Response: thank you for your suggestions, We have removed the word ‘severely’ and define the color as ‘by more than 20%’. Here we had less models because these are not DGVMs but photosynthesis modules incorporated by DGVMs, we have added one more column to link it with DGVMs. We have added report variability in the field-measured values.

Overall response: Thank you again for your excellent suggestions and support.

Other improvements:

We improved the appearance of Figure 5 for colour blind consideration.

Remaining reviewer comments

Your comments:	Our response:
R1 - I am satisfied with how the authors have addressed my comments. However, I have one remaining minor suggestion: In line 65, the text states that satellites underestimate fAPAR. Please replace 'satellites' with 'remote-sensing products,' 'satellite sensors,' or a similar term.<br><br>R2 - General comment: Thank for addressing the comments from the reviewers. In my opinion you improved the paper substantially.<br><br>Minor comments:<br>lines 308-320: check formatting<br>lines 322-324: “LPJ-GUESS was excluded as its documentation suggested against this calculation.” – change “suggested” to “advised” – a reason for this would also be helpful to see. How is it different from the other models in this regard?<br><br>lines 408-410: keep the decimals reported for different values consistent<br><br>lines 588-590: “At study site KOG, most models overestimate LAI (Figure S7), suggesting that LAI would not explain the underestimation of GPP at this site. At study sites BOB and KOG however, models averaged LAI is very close to field measurements” – Did you mean KOG overestimate, while BOB and ANK are close to field measurements?<br><br>Lines 618-625: check formatting<br><br>Line 646: “but also explains why this underestimation occurs” – I would suggest a bit of rephrasing as you only explained some extent of the underestimation.	R1 – Thank you, we have done so.<br>R2 – Thanks<br>lines 308-320: Sorry for the wrong font, we have corrected this.<br>lines 322-324: Thanks, we have added the reason as stated in the model manual; it is because PFTs can overlap in LPJ-GUESS (which simulates age-cohorts of different heights, and understorey vegetaion) […]. We have rephased the original paragraph a bit to make it concise.<br><br>lines 408-410:Thanks, now we keep one decimal place consistently.<br>lines 588-590: Sorry for this typos. Yes you are right, we have changed KOG to ANK<br>Lines 618-625: Apologies, we have corrected font size.<br>Line 646: Yes, we agreed that the investigation into the cause of GPP underestimation was not completed although we have identified fAPAR and Vcmax as key factors. We have rephrased it as “but also explores why this underestimation occurs”. We have modified the following sentence accordingly.

MS Title: Why models underestimate West African tropical forest productivity

Authors: Huanyuan Zhang-Zheng et al

Journal: Nature Communications

The manuscript titled "Why models underestimate West African tropical forest productivity" by Huanyuan Zhang-Zheng and colleagues evaluates how well TRENDY models, MODIS, and FLUXCOM reproduce annual mean GPP when compared against biometric GPP measurements from three sites. Based on the comparison, the authors conclude that the aforementioned products underestimate GPP for the three sites. The study then explores three potential sources that drive the model-data difference: (i) incorrect light-use efficiency (LUE) or photosynthetic capacity (maximum carbonxylation rate V_{cmax} and maximum electron transport rate J_{max}), (ii) incorrect fractional absorbed photosynthetic radiation (fAPAR), (iii) inappropriate assignment of plant functional types, and (iv) inaccurate climate variables. The results suggests that most of the data-model difference is due to fAPAR and photosynthetic traits. This conclusion is based on comparing simulations where the so called P-model is run (i) with satellite-based fAPAR while allowing the model to simulate LUE using optimality theory, and (ii) measured fAPAR and measured LUE that is based on V_{cmax} and J_{max}. The values of the latter two parameters are derived from light-saturated photosynthesis (A_{sat}) and light-and-CO_2 saturated photosysnthesis (A_{max}) field site measurements. The study addresses an important issue in carbon cycle modeling. Any disagreement between TRENDY model output and reference data like MODIS-GPP can be due to limitations of the models, the reference data and, which is most likely, a combination of both. This study tries to identify the sources of model-data discrepancy by integrating two types of models (TRENDY, P-model), global earth observations (MODIS, FLUXCOM), and in-situ measurements (biometric GPP, plant traits). I found the manuscript very interesting, but there is still room for improvement. Based on my review, I recommend that the manuscript may be considered for publication in Nature Communications after major revisions. Please find my comments below.

General Comments

- Your study is fascinating, but I would caution from overselling it. Your introduction section emphasizes how unusually high the biometric GPP values are compared to measurements in the Amazon (*greatly surpassing values measured in Amazonia*). Reading the corresponding reference shows that the difference is about 20%. Given measurement errors as well as spatial and temporal variability of GPP, I am not sure with how much confident this statement can be made. Personally, I would drop this part and focus on the model-data mismatch for the West African sites, instead. Also, I would stress that your results are valid for your sights, and that those sites are not necessarily representative for all of West Africa. I also don't see why the model-data mismatch shows *a lack of physiological understanding underpinning the high productivity*. Your manuscript shows that local parameter values reduce most of the model-data discrepancy, without the need of developing a new theory.

- You show that TRENDY models, MODIS, and FLUXCOM underestimate GPP when evaluated against biometric GPP. However, in one of the tree sites (ANK) you also have eddy covariance measurements, which are considerably lower (28 MgC ha^{-1} yr^{-1} on average) compared to biometric measurements (40 MgC ha^{-1} yr^{-1}). You are quick to dismiss the validity of the covariance data for a number of reasons that you list. That approach is not convincing. I suggest you either acknowledge that your biometric measurements are considerably larger compared to eddy covariance measurements, and that the differences between biometric GPP and MODIS GPP could be due to both, overestimation of the biometric values and underestimation of MODIS, or, you provide a more detailed analysis that convinces the reader why the eddy covariance data are underestimating the true values.
- To fully eliminate the role of classifications, I suggest you use tree-only GPP from TRENDY for the comparison against biometric GPP.

Detailed Comments

L1 *tropical forest productivity* is a vague term. I suggest you write *gross primary productivity*, instead.

L58 Typo (highest forests)

L61 Revise grammar

L97 You write that *The first field quantification of GPP in African forests [...] reported possibly the highest GPP value recorded in intact forests - greatly surpassing values measured in Amazonia* and cite your previous paper as a source (Zhang-Zheng, H. et al. Contrasting carbon cycle along tropical forest aridity gradients in W Africa and Amazonia, 2023). In your previous paper you show that the highest GPP value in Amazonia is estimated with about 42 MgC ha^{-1} yr^{-1}, while the largest GPP value in West Africa is estimated with about 49 MgC ha^{-1} yr^{-1}, a difference of about 20%. I think you want to make the case that the differences are larger for sites with comparable rainfall estimates. However, it is not clear from Figure 2 whether the rainfall amounts are comparable, as the horizontal axis lists stations in a qualitative, rather than quantitative, wet/dry order. It may very well be that GPP is larger in West Africa compared to the Amazon, but I think you need to do a better job in convincing the reader that this is indeed the case. GPP is subject to spatial and temporal variability. What convinces you that the differences in West African and Amazonian GPP assessed in your previous paper are not due to limited spatial and temporal sampling? In particular given that the stem biomass of the sites you are comparing are very similar. I don't intend to critique your previous paper, but the conclusions that you draw from your previous paper are the premise of your present manuscript, which makes them hard to ignore. I suggest you provide a more nuanced and quantitative description of the differences in West African and Amazonian GPP, or simply drop this comparison.

L101 You write that *It has not yet been explained [...] why this area has surprisingly high GPP. As models are designed based on current ecological theory, such a large discrepancy of multiple sites signals a lack of physiological understanding underpinning the high productivity.*

I think you are overstating the problem. According to your manuscript, the mismatch is driven by values of fAPAR, V_{max} and J_{max}. No new physiological understanding is therefore required or developed in this manuscript.

L117 The list also includes MODIS-GPP, not just vegetation models. Rewrite.

L124 Models typically simulate LAI and then express fAPAR as a function of LAI and canopy extinction coefficient. The key input would be the canopy extinction coefficient.

L140 Your study has 3 objectives and 4 hypotheses. You use different models and data sets to address your four objectives, and objective three will be tested using 4 different hypotheses. I suggest you enhance the clarity of your approach by providing a figure or table that illustrates the connection between objectives, hypotheses, data sets, and models.

L150 Please provide at least a brief summary of how biometric GPP is measured. I would also outline some of the advantages and disadvantages when compared to other approaches, such as the eddy covariance method.

L155 Revise grammar

L166 You have not yet defined V_{cmax} and J_{max}

L195 Using the term *models* for vegetation models, P-models, FLUXCOM and MODIS is confusing. I suggest you use a terminology that distinguishes among them. I agree that MODIS products contain a modeling component, but I would still refer to MODIS as a satellite-based product rather than a model. You could use the term *observation-based reference data set* for MODIS and FLUXCOM.

L227 Revise grammar

L230 You write that *Pmodel calculates a LUE from Jmax before calculating GPP*. Why are you mentioning this? Either delete or elaborate.

L243 Replace *plants functional types* with *plant functional types*

L243 Replace *climate variables* with *biased climate data*, or something along those lines

L245 Add the article *the* before Pmodel, here and below

L257 Why do you expect the differences to be slight? Rather than just referring to Figure S3, tell the reader how much of a difference you expect and why.

L262 Move this up to L247 where you discuss the topic first.

L274 Perhaps better to write *misclassification of land cover* rather than *confusion of land cover classification*

277 Replace *figure* with *Figure*

L284 Why do you do the comparison to the grid cell average? Why not compare the forest-only GPP from models against the GPP from field measurements?

L290 It would be nice if you could briefly iterate Objective 3 and Hypothesis 4

L290 Did you already define c_i/c_a?

L296 ERA-Interim was replaced by ERA5 in 2016. Why not use ERA5, instead?

L300 Compared to what other data products? Also, I would replace the term *best* with language more common in statistics, such as *most accurate*, or *least biased*, or *performs best across a range of statistical metrics*, depending on how the analysis was carried out.

L321 Please choose a different way to start this subsection. Also, use present-tense if the flux tower still exists.

L326 The S2 simulation keeps the land cover state fixed to its pre-industrial state of 1700. Please mention this. I imagine that the fraction of crops in the grid cells is therefore much smaller compared to the current state. That is something you may want to mention when explaining Figure S4 where you compare forest-only and whole-gridcell GPP.

L332 Replace *pixel* with *gridcell*

L372 Typo

L386 Replace *DGVMs models'* with *DGVMs'*

L414 Well, the in-situ fAPAR measurements are fairly constant across months, they don't really have a peak, except perhaps for KOG. I would write that *field fAPAR remains fairly constant during the year.* and then deleted the next sentence.

L421 Add that you are referring to Figure 4a.

L424 I suggest you include *Pmodel_null* in Figure 4 to assess the role of PPFD. This way the reader is not forced to jump to Figure S3.

L439 I don't find this section very convincing. First, your argument that GPP in forests and adjacent croplands should be equal is based on a single study that evaluates NPP, rather than GPP. I would be careful with extrapolating their conclusions to GPP and to take it for granted that their conclusions also apply to your sites. What if the adjacent cocoa plants have not yet reached maturity? What if management practices differ for your site? Also, there are considerable differences between TRENDY GPP computed for the whole grid cell versus tree PFTs only (Figure S4, e.g. ORCHIDEE-CNP). There is of course a very easy solution to this. You could base your study on GPP computed from forest-only PFTs rather than grid-cell level PFTs, which ensures you are comparing apples to apples.

L457 I suggest you briefly iterate the hypotheses at the beginning of each section.

L463 No need for bold font

L490 You write that *the biometric GPP is an average across multiple plots spanning several years, and its high productivity is supported by another field study using similar biometric method of GPP quantification* [47]. Reference 47 refers to the study by Morel et al (2019), which reports on NPP, primarily in coca plants. I did not find any mentioning of GPP in Morel et al (2019). Why does their study support your GPP measurements? Do eddy covariance measurement sites in the region report values comparable to your biometric GPP values? After reading further I now see that eddy covariance data estimate GPP in ANK with about 28 rather than 40 MgC ha^{-1} yr^{-1}, which does not support your high GPP values.

L505 I would mention this already in the introduction section.

L520 This is an important point, but it is not entirely true and requires a bit more discussion. Traditionally, the maximum carboxylation rate vcmax is a PFT-specific constant. So if the true vcmax differs among sites due to nutrient availability, then models cannot reproduce those patterns. However, some models simulate vcmax as a function of nutrient availability, such as nitrogen, in which case they could, at least in theory, reproduce those patterns. At the same time, at least in my experience, prescribed vcmax values generally lead to more realistic GPP values as simulating the N cycle has its own set of challenges.

L527 Please mention that v_{cmax} model parameters differ to measurements *compared to the measurements at your sites*. The values may still be appropriate at a global scale, as in-situ v_{cmax} measurement values differ considerably among sites.

L532 Well, the large v_{cmax} parameter value range is consistent with the wide range present in the TRY data base.

L551 Please replace *dramatically* with more neutral language.

L552 Further above (L486) you make the case that the difference between biometric GPP and values from MODIS and FLUXCOM are due to an underestimation of MODIS/FLUXCOM rather than an overestimation by the biometric GPP. The eddy covariance measurements that you cite here however indicate that the true GPP values could be lower than your measurements. It is very important that you consider all available evidence without prejudices, so please add this part to the discussion in L486. To iterate, the biometric measurements in ANK, for which you have eddy covariance data, are 40 MgC ha^{-1} yr^{-1}. The corresponding value from the eddy covariance measurement site has a mean value of 28 MgC ha^{-1} yr^{-1}. This is the same value as FLUXCOM (naturally, since the site is likely used as an input for FLUXCOM), and many TRENDY models are not far off. It is not convincing to simply dismiss this eddy covariance measurement site. I suggest you either acknowledge that your biometric measurements are considerably larger compared to eddy covariance measurements, and that the differences between biometric GPP and MODIS GPP could be due to both, overestimation of the biometric values and underestimation of MODIS, or, you provide a more detailed analysis about comparing the eddy covariance measurement site and your biometric values. For instance, reduce the comparison to the same years or months of measurement.

L562 Spell-out GLASS

L572 Typo

L575 Well, but this is perfectly fine because a model gridcell contains fractional PFT coverage, so the comparison with a resampled product is fair.

L583 You mean they vary considerably *among sites*? Also, there is a typo (like). Here is also a contradiction. If GPP varies considerably among sites, then those values are not necessarily representative for all of West Africa. How then can you conclude that almost all TRENDY models underestimate West African GPP? I would use more cautious language.

L586 Well, in the nutrient parameterizations I am familiar with, vcmax is expressed as a function of nutrient availability. So the discussion on nutrients is relevant in this context.

L578 Please use consistent notation (Pmodel or P-model)

L587 Typo (mode')

Figures

Figure 1 The figure gives the impression that there are hardly any fAPAR data at the locations of the sites as these loctions are all strongly affected by cloud cover. Is that so? Also, I appreciate panel (b) as it is intuitively easy to locate your study area, but I don't see any additional information in panel (a). Perhaps zoom in even closer if you want to show more details, or delete panel (a).

Figure 2 CLASSIC and JSBACH are missing in panel (a), how come? To my understanding, the TRENDY GPP data shown here consist of the gridcell value. Please replace this with the GPP associated with tree PFTs since your measurement sites only contain forest and no crops or grasslands. Also, I assume that the GPP values of insitu data, TRENDY models, MODIS data and FLUXCOM data are all based on the same period, right? Please add the corresponding period in the figure caption or the figure itself. Finally, I would add error bars that show the inter-annual variability.

Figure 3 Show how each experiment relates to the different hypotheses

Figure 4 I would include Pmodel_null here as a reference and to explain the difference between MODIS and Pmodel_P in site KOG, which, to my understanding, is caused by using site-level PPFD.
